# Pathway complexity in fibre assembly: from liquid crystals to hyper-helical gelmorphs†

**DOI:** 10.1039/d3sc03841f

**Published:** 2023-09-26

**Authors:** Rafael Contreras-Montoya, James P. Smith, Stephen C. Boothroyd, Juan A. Aguilar, Marzieh Mirzamani, Martin A. Screen, Dmitry S. Yufit, Mark Robertson, Lilin He, Shuo Qian, Harshita Kumari, Jonathan W. Steed

**Affiliations:** a Department of Chemistry, Durham University Durham DH1 3LE UK jon.steed@durham.ac.uk; b James L. Winkle College of Pharmacy, University of Cincinnati 231 Albert Sabin Way, Medical Science Building 3109C Cincinnati OH 45267-0514 USA; c School of Polymer Science and Engineering, University of Southern Mississippi 118 College Dr. Hattiesburg MS 39406 USA; d Neutron Scattering Division, Oak Ridge National Laboratory 1 Bethel Valley Rd. Oak Ridge TN 37831 USA

## Abstract

Pathway complexity results in unique materials from the same components according to the assembly conditions. Here a chiral acyl-semicarbazide gelator forms three different gels of contrasting fibre morphology (termed ‘gelmorphs’) as well as lyotropic liquid crystalline droplets depending on the assembly pathway. The gels have morphologies that are either hyperhelical (HH-Gel), tape-fibre (TF-Gel) or thin fibril derived from the liquid crystalline phase (LC-Gels) and exhibit very different rheological properties. The gelator exists as three slowly interconverting conformers in solution. All three gels are comprised of an unsymmetrical, intramolecular hydrogen bonded conformer. The kinetics show that formation of the remarkable HH-Gel is cooperative and is postulated to involve association of the growing fibril with a non-gelling conformer. This single molecule dynamic conformational library shows how very different materials with different morphology and hence very contrasting materials properties can arise from pathway complexity as a result of emergent interactions during the assembly process.

## Abstract 2

Pathway complexity results in unique materials from the same components according to the assembly conditions.

## Graphical abstract

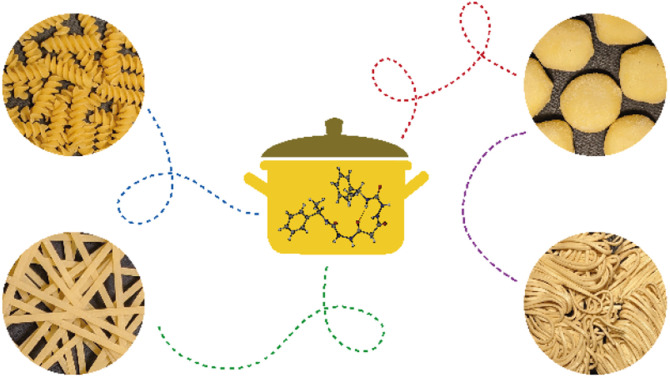

## Introduction

The conditions under which self-assembly occurs in the polymerization of supramolecular systems can give very different outcomes, and hence supramolecular materials with different properties arising from the same molecular building blocks. This concept is referred to as pathway complexity and can lead to equilibrium, metastable, kinetically trapped and dissipative structures depending on conditions such as concentration, pH, time, temperature and the presence or absence of templating species.^1,2^ An important example of such templating is the folding of proteins guided by molecular chaperones. These helper proteins prevent misfolding to give undesirable β-sheet structures such as amyloid fibres.^3^ Pathway complexity has been observed, for example, in zinc chlorin analogues of natural bacteriochlorophyll aggregates,^4^ the alternative formation of J-type or H-type porphyrin assemblies^5^ and in fuel-driven DNA nanostructures.^6^ Pathway complexity can also be influenced by seeding resulting in either linear or superhelical aggregates in *N*-annulated perylenes.^7^ Similarly, cooperative interactions between oligoalanine and non-chiral surfactants give rise to helical ribbons and tubules in the presence of antiparallel β sheets, while twisted ribbons result from parallel β-sheets or random coils.^8^ Triggered or templated transitions to helical or superhelical structures are also currently topical,^9–13^ and helical or superhelical structures can arise in inorganic materials and nanoparticle aggregates.^14,15^ In the context of supramolecular gels, helicity and chiral induction play a significant role in the final materials properties.^16^ For example, the combination of ultrasound, solvent and molecular chirality effects in supramolecular polymers formed from *N*-heterotriangulenes results in both kinetic and thermodynamic gel phases.^17^ There have been a number of reports on pathway complexity in supramolecular organogelators with factors such as chirality playing an important role.^18–22^ We have recently demonstrated that supramolecular gels can fit within the solid form and polymorphism landscape of molecular systems with gels potentially representing a first, metastable step in Ostwald's law of stages. The gel goes on to evolve into metastable and finally stable crystalline solids.^23^ However, there are very few reports of multiple gel states with different morphologies and different materials properties (which we term ‘gelmorphs’) arising from the same gelator.

**Fig. 1:**
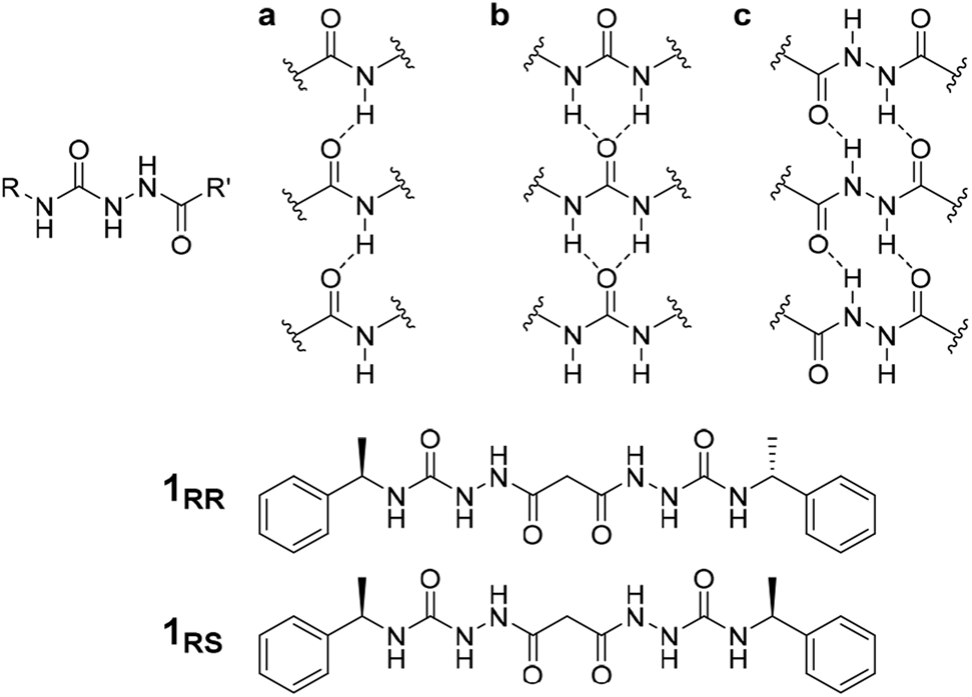
Structure of the acyl-semicarbazide group and target compounds of type 1 along with gel-promoting synthons by acyl-semicarbazides. (a) C(4) motifs between amides. (b) R^1^_2_(6) α-tapes between urea groups. (c) R^2^_2_(10) motifs in hydrazide-based gelators.

The acyl-semicarbazide moiety has the potential to generate high local density of hydrogen bond donors and acceptors. Unlike the more common urea gelators,^24–32^ acyl-semicarbazides have the potential to form at least three hydrogen bonding motifs that may be relevant in a gelation context (Fig. 1).^33–36^ They can behave in a similar way to amide gelators that typically form amide to amide C(4) hydrogen bonding motifs in graph set nomenclature,^37^ urea gelators that form R^1^_2_(6) α-tapes or hydrazide gelators that form R^2^_2_(10) synthons. This hydrogen bonding capacity makes their supramolecular self-assembly behaviour potentially very versatile and could yield responsive and effective low molecular weight gelators (LMWG). While acyl-semicarbazides have previously been utilized in supramolecular applications, including anion binding^38,39^ and self-assembly *via* molecular recognition,^40–42^ the number of reported acyl-semicarbazide gelators is very limited in comparison to other LMWG classes. The Zentel group reported the first example of mono(acyl-semicarbazide) gelators in 2005 and have since reported a selection of structurally related compounds as effective gelators, highlighting the capability of acyl-semicarbazides as versatile organogelators.^34,35,43,44^ Most acyl-semicarbazide-based gelators are bis(acyl-semicarbazide)s, with the majority coming from recent studies by the Palanisamy group.^33,36,45^

In this study, different self-assembly pathways according to the stimulus applied have been observed for bis(acyl-semicarbazide) derivatives, yielding different materials formed from the same gelator including lyotropic liquid crystal droplets, weak transparent gels made of thin fibril bunches, stronger gels comprising fibrous tapes and very stiff opaque gels made of thick, homochiral hyper-helical fibres. The system represents a unique example of pathway complexity giving rise to at least four different assembly modes from the same molecular building block and highlights the importance of the control of microscale structure on bulk materials properties.

## Results and discussion

### Crystallization and gelation behaviour

The homochiral bis(acyl semi-carbazide) gelator 1_RR_, its enantiomer 1_SS_, and the *meso* diastereoisomer 1_RS_ (Fig. 1 and ESI Section 1†) were designed as LMWGs to incorporate both intra- and inter-molecular hydrogen bonding functionality, in conjunction with a central methylene group providing conformational flexibility, phenyl groups which balance solubility and enable intermolecular π–π interactions, and chirality which restricts the degrees of freedom driving the anisotropic self-assembly. The presence of conformationally restricted amide functionalities in conjunction with intramolecular hydrogen bonding means that the compounds effectively exist as a small virtual library of slowly interconverting conformers. Both enantiomers of the *rac* diastereoisomer were readily synthesized through the addition of malonic dihydrazide to either (*R*) or (*S*)-(−)-α-methylbenzyl isocyanate. The *meso* diastereoisomer 1_RS_ was obtained using a stepwise strategy (see ESI Section 1† for synthetic details).

**Fig. 2:**
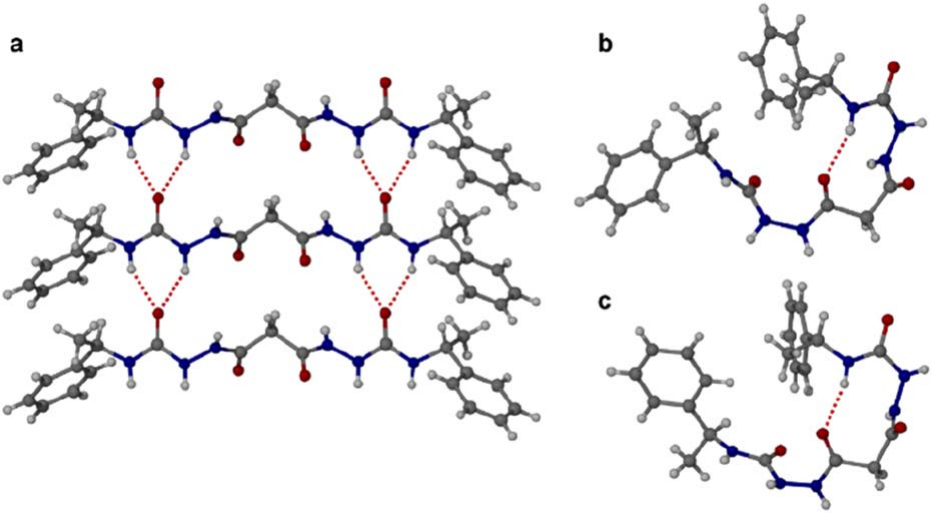
Single crystal X-ray diffraction structures of bis(acyl-semicarbazide) derivatives. (a) Urea α-tape packing in the linear conformer A of 1_RR_ found in all of the enantiopure ethanol solvate, hydrate and racemic acetonitrile solvate needle-shaped crystals. (b) The bent conformation B adopted by the unstable plate-shaped polymorph of the racemic acetonitrile solvate showing the intramolecular hydrogen bond. (c) The analogous bent conformation in the structure of the *meso* diastereomer 1_RS_.

Crystallographic characterization of 1_RR_ revealed remarkable conformational solvatomorphism. Needle shaped crystals of enantiomerically pure 1_RR_ as an ethanol solvate were obtained by slowly cooling a solution in ethanol–water (1 mL : 0.8 mL). An isostructural dihydrate material was obtained in a similar way from 1 : 1 dioxane–water. Crystallization of a racemic mixture of 1_RR_ and 1_SS_ by slow cooling of a solution in acetonitrile resulted in the concomitant formation to two crystal morphologies (needle and plate shaped) of racemic 1_RR/SS_. These two materials proved to be strikingly different polymorphic acetonitrile solvates. All samples were characterized by X-ray crystallography, in the case of the latter three using the I19 instrument at the Diamond Light Source synchrotron. Interestingly, while the needle crystals of 1_RR/SS_ are highly stable, the plates decompose rapidly under the X-ray beam and hence the data is sufficient only to reveal the gross conformational details. The structures of all three types of needle shaped crystals (enantiopure ethanol solvate, dihydrate and racemic acetonitrile solvate) reveal a linear conformation (conformation A) with both urea functionalities in *anti*–*anti* configuration (Fig. 2a). The ethanol solvate and the dihydrate are isomorphous. The linear A molecules stack *via* a typical 6-membered ring urea–urea hydrogen bond synthon, generating the common urea α-tape arrangement (R^1^_2_(6), Fig. 1b). The remaining NH groups interact with the included solvent. In contrast, the molecules in the metastable acetonitrile solvate plates adopt a bent conformation (B) with *anti–anti* and *syn*–*anti* urea configurations (Fig. 2b). The bent molecular shape is facilitated by an intramolecular hydrogen bond forming a 9-membered ring, S(9). The structure also forms unusual R^2^_2_(14) and R^2^_2_(18) motifs. It seems likely that the concomitant polymorphism of 1_RR_ arises from the presence of both conformers in solution undergoing slow exchange such that they behave as different species on the crystal nucleation time scale. In fact, the ^1^H NMR spectrum of 1_RR_ reveals the presence of three conformers in solution in slow exchange (*vide infra*). The instability of the B polymorph seems to arise from the fact that the acetonitrile solvent is loosely bound in a hydrophobic pocket in the structure and does not interact directly with the semicarbazide. In contrast, in the A structures the ethanol, water and acetonitrile solvent molecules directly hydrogen bond to the NH groups that are not tied up in the urea α-tape motif.

Crystallization of the *meso* diastereomer 1_RS_ resulted in plate-like crystals from ethanol using similar conditions to those employed to obtain the A needle-shaped crystals of 1_RR_ from this solvent. The structure revealed that 1_RS_ molecules adopt a bent conformation in the solid state, very similar to the B conformer observed in the plate-like crystals of 1_RR/SS_ from acetonitrile and exhibiting the same intramolecular 9-membered hydrogen bonded ring (Fig. 2c). The centrosymmetric nature of the structure means that it is closer packed than the metastable acetonitrile solvate B structure of 1_RR_ and is unsolvated. As a result, the crystals are stable, and the structure determination is of good precision.

A gelation screen was undertaken with 1_RR_, 1_SS_ and 1_RS_ (ESI Section 3†) using a total of 43 solvents spanning the polarity spectrum. Samples were prepared by dissolving the gelator in hot solvent at 1% w/v, allowing the mixture to cool to room temperature and then sonicating for 1 minute at room temperature. The gels formed are thus sonogels.^46^ This screen revealed that the *meso* form 1_RS_ is a non-gelator in all the solvents tested. This behaviour is consistent with the external morphology of the crystals which is two-dimensional rather than having a fibre-like high aspect ratio. In contrast, the resolved chiral gelators 1_RR_ and 1_SS_ are able to gel a broad range of solvents (21 of 43) at 1% w/v including media as diverse as water and 1,3,4-trichlorobenzene (Table S2†). These enantiomers are therefore ambidextrous gelators as they gel water and organic solvents. Ambidextrous gelators are relatively uncommon and tend to be amphiphiles, since amphiphilicity can promote solubility in both organic solvents and water.^47,48^ The inversion of handedness of just one chiral centre compared to 1_RS_ thus appears to inhibit aggregation in three dimensions, reducing crystallinity and giving rise to high aspect ratio fibres. In addition, use of a racemic mixture of 1_RR_ and 1_SS_ almost completely turns off gelation behaviour and gives rise to precipitates in every solvent with the one exception of nitrobenzene which forms a transparent gel.

**Fig. 3:**
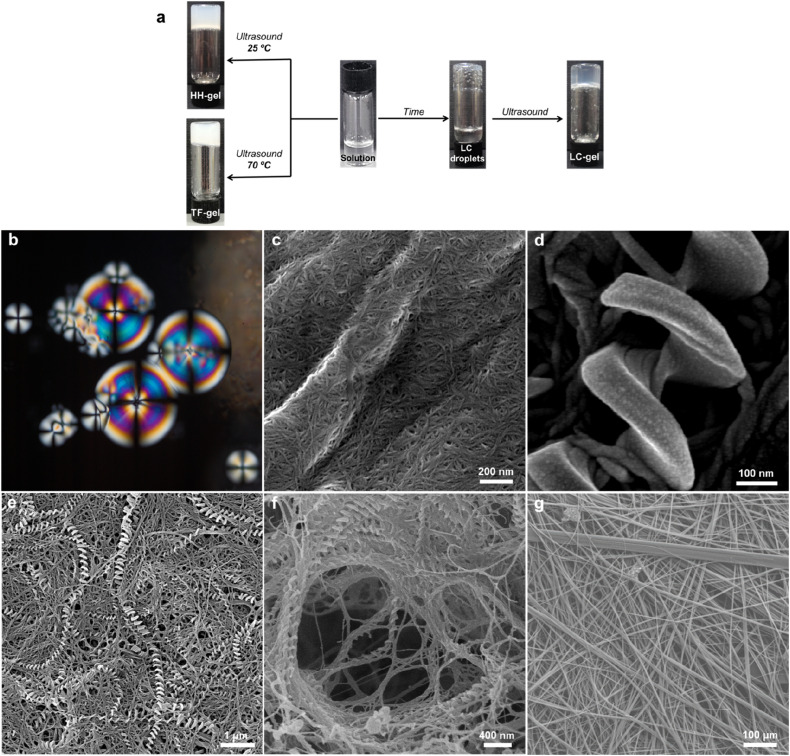
(a) Macroscopic behaviour of the 1_SS_ or 1_RR_ – 1,4-dioxane system as a function of the applied stimulus. (b) Polarized optical micrograph of LC droplets obtained from 1_RR_ in 1,4-dioxane without sonication showing a Maltese-cross pattern. (c) SEM image of an LC-Gel showing thin-fibril bundles. (d and e) SEM images of a detail of (d) a left-handed hyper-helix from a HH-Gel of 1_SS_ dried in air and (e) lower magnification view of the same sample where both hyper-helical and tape fibre morphologies can be observed. (f) SEM image of freeze dried HH-Gel of 1_RR_. (g) Low magnification SEM image of the TF-Gel.

An intriguing phenomenon is observed when a single enantiomer of 1_RR_ or 1_SS_ is dissolved in acetone or 1,4-dioxane at concentrations above 0.5% w/v by heating and subsequent cooling to room temperature without sonication. This process results in phase separation and the formation of spherical liquid crystalline droplets within few days of cooling. These mesophases retain considerable solvent and are thus lyotropic. They exhibit a birefringent Maltese cross that rotates with the polarization axis of light (Fig. 3b). Sonication of this mesophase droplet suspension results in the immediate formation of an optically transparent gel (LC-Gel) within seconds. In contrast, sonication at room temperature of the 1_RR_ solution after cooling but prior to mesophase formation results in gelation within hours to give a robust, opaque gel with a hyper-helical morphology (HH-Gel). A very different gel with a linear tape fibre morphology is obtained upon sonication of a hot solution of 1_RR_ at 70 °C followed by standing at room temperature (TF-Gel).

Analysis of these three different types of air-dried and freeze-dried gel by SEM shows that the xerogel of the LC-Gel comprises thin fibril bunches (Fig. 3c). In contrast the dried HH-Gels obtained after cooling then sonicating a fresh clear solution of 1_RR_ or 1_SS_ in 1,4-dioxane gives large, impressive homochiral hyper-helical fibres (Fig. 3d–f and S11c and d†). The handedness of the fibres depends on the enantiomer used with the RR enantiomer giving right-handed or *P* helices while the SS enantiomer gives left-handed or *M* helices (Fig. S12†). The high temperature TF-Gel has a very different appearance comprising long, straight ribbons of varying thickness Fig. 3g. The ribbons form from fibres of ∼160 nm in width (Fig. S11a†), which then associate into thicker fibrillar bundles, and flatter ribbons of varying width observed between 2–11 μm. Depending on precise handling conditions the non-liquid crystal samples often exhibit a mixture of hyper-helical and tape fibre morphologies (Fig. 3e). Thus, these three types of gel arising from the same gelator represent gelmorphs that arise as a result of pathway complexity in their formation.

### Conformational studies in solution

**Fig. 4:**
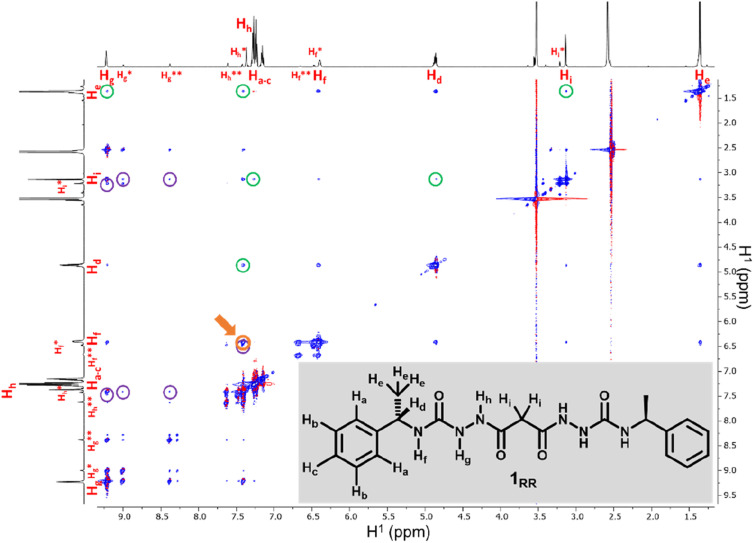
600 MHz NOESY of 1_RR_ in 1,4-dioxane-*d*_8_. Peaks highlighted with purple circles are due to spatial interactions between protons of the major isomer and protons on the second more abundant one labelled with asterisks. Cross-peaks surrounded with green circles are due to proximity effects between hydrogen that are so far apart in that would not be observed as intramolecular interactions. The cross-peak highlighted in orange and pointed with an arrow is due to *H*_h_ being close to *H*_f_.

SEM studies have revealed three xerogel morphologies, while X-ray crystallography shows compounds of type 1, in the solid state, can adopt either extended linear or bent conformations, the latter involving an intramolecular hydrogen bond. To understand the conformation of the species in solution and their dynamic behaviour we used several Nuclear Magnetic Resonance (NMR) techniques to study solutions of 1_RR_ in 1,4-dioxane-*d*_8_ at 298 K. The ^1^H NMR spectrum of 1_RR_ in dioxane (Fig. 4 and S3†) reveals the presence of three species with ratios 78 : 21 : 1. NOESY (Fig. 4) complemented with ROESY (Fig. S5†) studies showed that these species are slowly interconverting, probably due to the restricted rotation of the amide bonds,^49^ a process that leads to conformational isomerism. The conformation change was also apparent in the ^1^H NMR spectra acquired at temperatures ranging from 25 to 85 °C (ESI Section 4†). At 85 °C, the signals from the same kind of protons coalesce, leaving one average conformation. Signals from the three conformers reappear when the temperature is lowered to 25 °C. The major conformer at 25 °C, characterized by a single *H*_g_ signal (9.21 ppm), is symmetric, while the second most abundant conformer is asymmetric because it shows two *H*_g_ signals (9.01 and 8.39 ppm). In both cases, the dihedral angle between NH_g_ and NH_h_ must be closer to 90° than to 0 or to 180° because the measured coupling constant between *H*_g_ and *H*_h_ is less than 2.5 Hz.^50^ This is consistent with the near 90° angles observed in all of the X-ray structures.

**Scheme 1:**
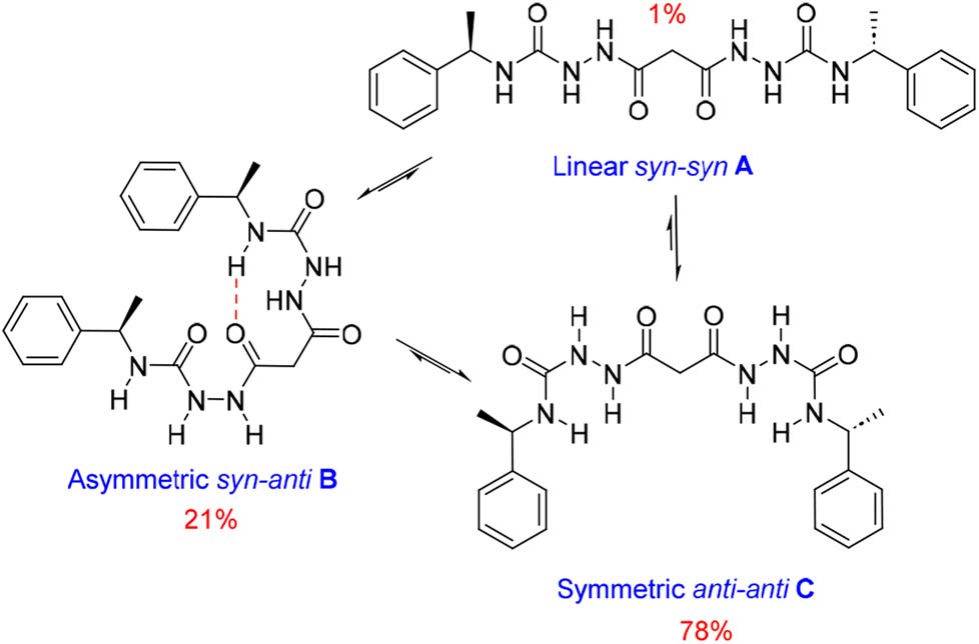
Conformational equilibria of 1_RR_ derived from solution-state NMR and X-ray crystallography.

We also estimated the distances between several protons in the major conformer in solution using the NOESY shown in Fig. 4. Distances analysed by this method should be considered approximations, with over- or under-estimations occurring due to experimental factors, but they are useful when used with complementary data, as in this case. We compared the distances derived from the NOESY with those derived from the two known conformations obtained from SC-XRD (Table S4†). The comparative analysis of distances detailed in ESI Section 4,† indicates that the nitrogen-attached urea hydrogen atoms in the major conformer adopt an *anti*-arrangement on both sides of the molecule (Fig. S6†). Evidence for this comes from the significant cross-peak nOe signal between NH_f_ and NH_h_ implying a separation of around 2.8 Å (highlighted in Fig. 4). In contrast, this distance is 4.3 Å in the linear *syn–syn* conformer observed crystallographically (conformer A in the hydrate and ethanol solvate structures), while on the *anti* side of the crystallographic *syn*–*anti* conformer B the distance is 2.8 Å, consistent with the NOESY-derived distance. The symmetric nature of the major conformer means that it cannot be assigned to conformer B, and the symmetric *anti–anti* conformer C is therefore the most likely major solution form, Scheme 1. The asymmetric structure of the second more abundant conformer in solution is consistent with the intramolecular hydrogen-bonded *syn*–*anti* conformer B and hence the least abundant conformer in solution is likely to arise from the linear *syn–syn* conformer A observed crystallographically. This situation in which it is the least thermodynamically stable species in an equilibrating mixture that is observed to crystallize has been observed previously^51^ and can dominate the observed solid as the solution equilibrium shifts to accommodate the depletion as the species crystallizes.

The NOESY spectrum of 1_RR_ also exhibits several cross-peaks between protons that are expected to be far apart in the molecule (Fig. 4, peaks highlighted with green circles). These may be the result of intermolecular association. To investigate whether molecules self-assemble in solution, we ran a diffusion ordered spectroscopy (DOSY) experiment (ESI Section 4†). This allowed an estimate the molecular mass of the entities present in solution relating the diffusion coefficients derived from the experiment to a molecular mass.^52^ The estimated molecular mass average of the major isomer is 1081 Da, while the mass of a single molecule is 426.5 Da. Similar results are observed for isomer B and hence this indicates that both conformers B and C may dimerize in solution.

An interesting evolution of the ^1^H NMR spectrum over time was observed (Fig. S7†). The NH protons shift to lower frequencies accompanied by broadening of the spectrum. After 5 days, the evolution process stops and is accompanied by the formation of the liquid crystal droplets. This highlights the relatively slow assembly of the aggregates leading to the LC droplets.

### Gel structure and evolution

**Fig. 5:**
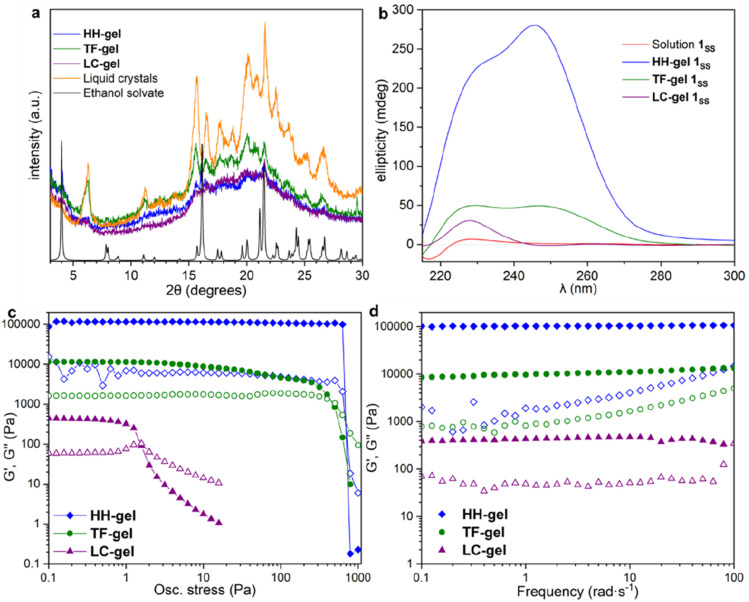
X-ray powder diffraction, circular dichroism and rheology data for HH-, TF- and LC-Gels. (a) The XRPD patterns obtained from freeze dried HH-Gel, LC-Gel, TF-Gel and freeze-dried LC droplets are all the same solid form and do not correspond to the XRPD pattern calculated from the type A ethanol solvate single crystal structure. (b) CD spectra of solutions and gels; all samples studied contained 10 mg mL^−1^ solution of 1_SS_ in 1,4-dioxane. (c and d) Oscillatory rheometry for HH-, TF- and LC-Gels; solid symbols *G*′, hollow symbols *G*′′; (c) stress sweep and (d) frequency sweep.

X-ray Powder Diffraction (XRPD) was used to analyse xerogels obtained by freeze drying gels made of hyper-helical fibres (HH-Gel), linear tape fibres (TF-Gel) and the gel derived from LC droplet suspensions (LC-Gel), together with the dried LC droplets separated from the solution (Fig. 5a). Freeze drying the gel limits the possibility of solid form conversion as a result of the drying process. All of the gel samples are of the same low crystallinity solid form and that form does not correspond to the XRPD patterns calculated from either the linear type A or bent type B MeCN solvate crystal structures (Fig. S17†). In the XRPD pattern derived from the linear type A ethanol solvate crystal structure the intense first peak at 4.0° arises from the (001) reflection which arises from the long 22.1 Å axis and corresponds to the length of one extended conformer of type A. The freeze-dried gel phase patterns all lack this peak and hence do not have unit cells large enough to accommodate a type A conformer. Instead, they all have a peak at 6.3° corresponding to a *d*-spacing of 14.0 Å. The non-solvated bent B-type structure of the RS diastereoisomer has the (100) peak at 6.55 Å (Fig. S17†), *d*-spacing 13.5 Å, which is approximately the length of a bent type B molecule. Hence, the unit cell for all of the xerogels seems to be too small to accommodate either conformer A or C and the gels appear to be based on bent conformer B. This smaller unit cell phase is also observed for xerogels obtained from a variety of other solvents, namely acetonitrile, propan-1-ol and chlorobenzene (Fig. S14†). If, instead of freeze-drying the gels, xerogels are prepared by drying in air, the resulting XRPD patterns reflect mixtures of the freeze-dried phase and a phase isostructural with the type A ethanol and water solvates. It seems likely that the drying process results in conversion over time of the initial xerogel to the thermodynamically stable type A form. This conversion reflects the metastability of the type B acetonitrile solvate compared to the concomitant type A analogue observed during the single crystal analysis. Hence it seems likely that the folded type B conformer is less soluble than type C and forms the basis both for the initial crystalline material and all gels. However, the much more significant intermolecular interactions formed by the A conformer make the type A crystals the thermodynamic sink in the solid state.

### SANS analysis of gel formation

The effect of solvent is often not captured by XRPD or SEM analysis of xerogels. Herein, we utilized small-angle neutron scattering (SANS) to study gel evolution as a function of time. Neutrons interact with nuclei of atoms which renders them sensitive to isotopic exchange to enhance contrast. Hence, we prepared gels in deuterated 1,4-dioxane and collected SANS data at several time points until equilibration. Samples were measured on the GP-SANS beamline of high flux research reactor (HFIR), Oak Ridge National Laboratory (ORNL), USA.^53^ The data were taken at various time intervals according to the speed of gelation, and at different detection distances and wavelengths to cover the *q*-range of 0.0015–0.5 Å^−1^, for a period of 26 h (HH-Gel), 20 h (TF-Gel) and 14 h (LC-Gel) (ESI, Table S5†).

**Fig. 6:**
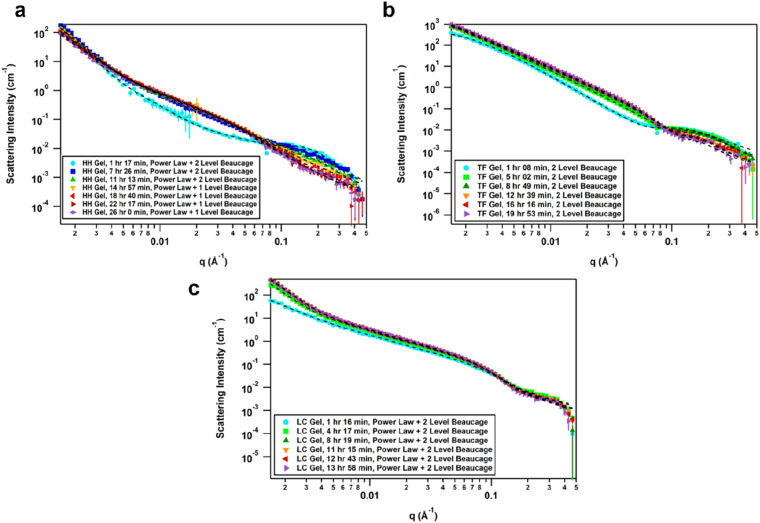
(a) Time-resolved SANS data of HH-Gel, fitted with a summed unsmeared power law + 2 level Unified model for the first three time points and then power law + 1 level for the remaining four time points. (b) Time-resolved SANS data of TF-Gel, fitted with a summed unsmeared 2 Level model. (c) Time-resolved SANS data of LC-Gel, fitted with a summed unsmeared power law + 2 level model.

An overlay of the reduced, background-subtracted data is shown in Fig. 6. A broad peak at *q* ∼ 0.15 Å^−1^ can be seen in the HH-Gel (Fig. 6a) and TF-Gel (Fig. 6b) data, which does not significantly shift as the gels age. The intensity of the peak, however, does gradually decrease over time. This indicates that this signal arises from a primary building block that is crosslinked to form larger structures over time, resulting in the high-*q* peak decreasing in intensity while the low- and mid-*q* scattering intensities increase. This is apparent especially in the HH-Gel scattering data starting approximately at the 15 h mark, where the high-*q* shoulder indicative of the small building block structure disappears. A similar trend can also be seen in the LC-Gel (Fig. 6c). The strong low-*q* scattering present in the HH-Gel and LC-Gel appears to be the signature of a larger length scale that is beyond the resolution of the instrument. In comparison, the TF-Gel shows two main structural regimes probed *via* SANS, but because the larger of the two regimes lacks a Guinier region, it is not fully within the SANS range. This agrees with SEM images that show long fibres, which are much longer than the maximum length scale of about 400 nm visible to SANS (ESI Fig. S13†).

The scattering data best fitted to 1- or 2-level Unified Exponential/Power Law model.^54,55^ An additional power law model was added to the Unified Exponential/Power Law model to account for excess scattering in the low-*q* regions of the HH- and LC-Gel data. The selected fitting parameters shown in Table 1 demonstrate how the HH-Gel, TF-Gel, and LC-Gel form over time, starting when the gelator is sonicated to form the sol phase. The Unified model can be expanded to consist of multiple structural levels to model scattering data over a wide *q*-range, with each structural level consisting of a Guinier exponential form and a power law decay.^54^ As each structural level corresponds to a certain length scale, information about the local, overall, and intermediate structure of the scattering particle can be gained. The radius of gyration of a particle, *R*_g_, obtained from the Guinier region, is the radial distance from the axis of rotation to a hypothetical point that would have the same moment of inertia as the particle, if the mass of the particle were centralized at that point. The magnitude of the power law value can represent the surface smoothness if at higher values (3 for rough surfaces, 4 for smooth surfaces), mass fractal networks (2–3), or polymer–solvent interactions (1.6 for swollen chains, 2 for Gaussian chains/theta condition, or 3 for collapsed coils). The need for a power law model to model the full *q*-range for the HH- and LC-Gels suggests that the low-*q* data only shows the tail of a potential third structural level. For the first 11 h after the HH-Gel formed, the two apparent structural levels plus the tail-end of a third were present. Comparing *R*_g,1_ and *R*_g,2_ in size and how they change over the first 11 h, *R*_g,2_ corresponds to the primary structure or building block of the gel fibre which stays constant at 21 Å (comparable to one molecular length), while *R*_g,1_ is the radius of gyration of the fibre itself. Pow_2_ indicates that the building blocks of the fibre are collapsed chains, while Pow_1_ suggests that the overall fibre is in the theta condition (neither collapsed nor swollen).^55^ After 15 h, the primary building block disappeared in this material, leading to the successful use of a 1-level unified model, suggesting that the hyper-helical structure seen in the SEM (Fig. 3d) is begins to dominate at this stage. With further aging, the fibre becomes smaller (*R*_g,1_) and undergoes significant branching as indicated by Pow_1_; at the same time, the overarching gel structure becomes increasingly dense due to the branching of the fibres until the gel network is fully formed ([−]power). After aging for approximately 22 h, no further change is observed, indicating that the gel fibre and overall structure are fully formed. The scaling factor, *A*, increases over time for both the HH-Gel and LC-Gel, demonstrating that the gels become denser as they age. Larger values of *A* for the HH-Gel compared to that of the LC-Gel also indicate that the HH-Gel is denser. The larger value of *A* for the HH-Gel is consistent with a denser network and stronger gel than that of the LC-Gel. This observation is consistent with the gels' rheology (*vide infra*).

**Table 1:** Select fitting parameters of power law + 2 Level Unified model fitted to each time point for each gel. The gel age is the time after sonication at which the data was collected. The HH-Gel only required a 1 Level model for the last four time points, so the second level parameters were not used. Likewise, the TF-Gel did not require a power law term to be fitted, so those parameters were not used. The numbers in parentheses represent the uncertainty in the last significant figure of each value. Full fitting parameters can be found in the ESI

Gel	Age	Coefficient, *A*	(−) Power	*R*_g,1_ (Å)	Pow_1_	*R*_g,2_ (Å)	Pow_2_
HH-Gel	1 h 17 min	1.0(3) × 10^−9^	3.96(6)	450(30)	2.41(8)	21.4(3)	3.0(1)
	7 h 26 min	2.9(7) × 10^−9^	3.84(4)	190(7)	1.95(5)	21.8(7)	2.7(1)
	11 h 13 min	8(2) × 10^−9^	3.64(4)	166(5)	2.00(4)	20(1)	3.0(3)
	14 h 57 min	1.9(4) × 10^−8^	3.50(3)	146(3)	2.15(1)	—	—
	18 h 40 min	1.4(2) × 10^−7^	3.15(3)	125(3)	2.32(2)	—	—
	22 h 17 min	2.8(5) × 10^−7^	3.02(3)	119(3)	2.45(2)	—	—
	26 h 0 min	2.7(5) × 10^−7^	3.01(3)	124(3)	2.46(2)	—	—
TF-Gel	1 h 08 min	—	—	860(10)	3.146(5)	26.2(4)	2.20(6)
	5 h 02 min	—	—	1270(20)	2.720(2)	21.0(2)	3.3(1)
	8 h 49 min	—	—	1150(10)	2.579(2)	19.8(2)	4.0(2)
	12 h 39 min	—	—	1170(10)	2.542(2)	20.9(3)	4.0(3)
	16 h 16 min	—	—	1180(10)	2.546(2)	20.9(3)	4.0(3)
	19 h 53 min	—	—	1190(10)	2.557(2)	21.4(4)	4.0(4)
LC-Gel	1 h 16 min	2.8(8) × 10^−5^	2.24(5)	128(4)	1.89(9)	21.3(6)	2.5(1)
	4 h 17 min	8(1) × 10^−9^	3.74(3)	205(2)	1.71(2)	22.4(3)	2.57(8)
	8 h 19 min	5.7(6) × 10^−9^	3.88(2)	198(2)	1.71(2)	22.5(3)	2.04(7)
	11 h 15 min	6.8(7) × 10^−9^	3.85(2)	188(2)	1.74(2)	22.9(2)	2.04(7)
	12 h 43 min	6.4(7) × 10^−9^	3.86(2)	189(2)	1.73(2)	23.0(3)	2.15(8)
	13 h 58 min	1.1(1) × 10^−9^	3.76(2)	184(2)	1.72(3)	22.7(4)	2.1(1)

The TF-Gel maintains two apparent structural levels for the entire aging time. Like the HH-Gel, *R*_g,1_ and Pow_1_ reflect the gel fibre while *R*_g,2_ and Pow_2_ reflect the persistence length of the primary structure. The persistence length of the fibre straightens and smooths over time to form a smooth fibre surface, where Pow_2_ = 4, while shortening until it maintains a *R*_g,2_ of 21 Å. The *R*_g,1_ of the overall fibre continues to gradually increase over the full observation time but is too long to be fully resolved by SANS. Pow_1_ indicates that the fibre has a rough interface at the beginning, but then branches begin to form network structures indicative of mass fractal dimensions. The TF-Gel forms faster than the HH-Gel, as all significant changes are complete by the 12 h mark. Taken together, SANS suggests that the TF-Gel structure consists of long, straight fibres with plentiful branching and cross-linking to form a dense gel structure, consistent with the SEM data. With regards to the LC-Gel, the building block *R*_g,2_ lengthens slightly to approximately 22.8 Å in conjunction with Pow_2_ decreasing to 2.1 to indicate it is in the theta condition. The fibres lengthen to about 190 Å and are swollen, as shown by Pow_1_ decreasing to 1.7. The low-*q* scattering decay increased to 3.85, indicating that the overall gel structure went from a collapsed mass fractal to a fairly smooth interface within 8 h. The low-*q* scattering intensity also increased over time, correlating with an increasing amount of gel structure. Lastly, the LC-Gel fully formed the fastest of the three gels, having finished forming after approximately 8 h. These parameters suggest that the LC-Gel is a relatively sparse gel structure with little branching or cross-linking of its short, swollen fibres. In summary, the SANS analysis indicates that the HH-Gel has a dense structure given its degree of branching and cross-linking. Coupled with the helical fibres observed in the SEM figures, it is likely to be a strong gel. The TF-Gel also has significant branching and cross-linking with long fibres, likely making it a strong gel that does not take as long to mature as the HH-Gel. The LC-Gel is likely to be the weakest of the three, given its low degree of branching and swollen fibres.

### Gel evolution by CD spectroscopy

To understand the aggregation of 1_RR_ and 1_SS_ in 1,4-dioxane, circular dichroism spectroscopy (CD) was also employed and is a useful tool in studying the evolution of mesoscale chiral structures.^56^ The CD spectrum of a freshly prepared solution of 1_SS_ shows a bisignated band with a negative maximum at 217 nm and positive at 230 nm which indicates a right-handed helical structure,^57,58^ followed by a wide and less intense cotton positive band centred between 260–265 nm (Fig. 5b). The maximum at 230 nm is associated with the transition n–π* of the amide carbonyl groups.^59^

The CD ellipticity of HH-Gel is much more intense than that of the gelator solution (Fig. 5b). Also, the spectral profile differs considerably between gel and solution, which is an indicator of a conformational change as a result of the self-assembly process. CD spectra were recorded in a thin 1 mm cuvette as a result of the high optical density and scattering of the gels. It is notoriously difficult to eliminate linear dichroism (LD) effects in the CD spectra of aligned media^60^ and the shape of the 1 mm cuvette does not allow for averaging by rotating it by 90°. Hence it is highly likely that the recorded spectra include both LD and CD contributions. Nonetheless they give information about the evolution of the gels over time and the shape change in the ellipticity graph suggests helical hierarchy. As the gel forms the 217 nm Cotton negative band observed in solution disappears, but the ellipticity of the one centred at 230 nm increases from 6 to 227 mdeg. Also, a new and more intense positive band (280 mdeg) appears centred at 246 nm. The substantial ellipticity increase at 230 nm can be explained by aggregation of monomers into gel fibres in a way that dipoles of carbonyl groups are one-dimensionally aligned. However, the new 246 nm band cannot be due to an antenna effect from the one dimensional self-assembly, so it has to be related with new left-handed helical features that appear as a result of the supramolecular organization, consistent with the observed hyper-helical morphology of the gel (Fig. 3d and e). In contrast, formation of the LC-Gel results in a five-fold increase in the intensity of the 230 nm band, but the band at 246 nm does not appear. Thus, the LC-Gel is likely to be self-associated in the same way as the HH-Gel as indicated by XRPD, but no helical features appear as a result of the supramolecular organization, an observation that is consistent with the SEM images which indicate linear fibrils (Fig. 3d). Interestingly, the bisignate band found in solution disappears after supramolecular polymerization to yield either the HH-Gel or LC-Gel, which implies that the right-handed helical features of 1_SS_ molecules in 1,4-dioxane solution disappear because of the self-assembly.

A gel prepared by sonicating a solution of 1_SS_ at 70 °C for 60 s exhibits predominantly linear tape (TF-Gel) mixed with a few hyper-helical fibres (HH-Gel) (Fig. 3g and S11a†). In the CD spectra of this gel (Fig. 5b), both 230 and 246 nm bands are present, but the 246 nm band associated with the helical features is significantly less prominent. The morphological differences between gels obtained sonicating at 25 or 70 °C are a clear indicator of how crucial the transformation provoked by ultrasound is to drive the evolution of the system.

### Gel rheological properties

The different fibre structures of the HH-, TF- and LC-Gels affords the ability to tune the mechanical properties of the gel formed by 1_RR_ based upon the assembly pathway. Fig. 5c shows oscillatory stress sweep data for the three gels. The LC-Gel is the weakest, with a storage modulus (*G*′) of ∼400 Pa. The TF-Gel modulus is over an order of magnitude higher, while the HH-Gel displays a remarkable elasticity, with a *G*′ of ∼105 Pa. The yield stress further highlights the difference in properties of the three gels. This was determined as the end of the linear viscoelastic region, shown by the dashed line, and is 800, 4 and 0.6 Pa for the HH-, TF- and LC-Gels respectively. The lower modulus and yield stress show the LC-Gel is much weaker than the TF- and HH-Gels, while the HH-Gel is very strong.

Frequency sweep data for the different gels is shown in Fig. 5d. All three systems show a relative independence to frequency in the storage modulus, with *G*′ an order of magnitude greater than the loss modulus (*G*′′), characteristic of gels. The results confirm the solid-like behaviour of the gels on the time scale of the measurement. Such plateau behaviour with frequency is a result of cross-links within the gel. These can be formed from physical entanglements between fibres, constraining relaxation, or by branch-point defects in the fibre structure acting as a network junction point. The high modulus of the HH-Gel suggests this system contains the most cross-links, while the LC-Gel the least.

To estimate the size of the network structure a generalized Maxwell model^61^ was fitted to the frequency sweep data^62^ in RepTate.^63^ From this the polymeric cross-link density, *ρ*_*x*_, is determined from Flory's theory by eqn (1):^64^1
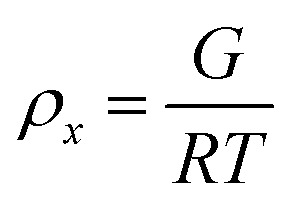
where *R* is the ideal gas constant, *G* the elastic shear modulus and *T* the temperature. This allows calculation of the average network mesh size, *ξ*, from eqn (2):2
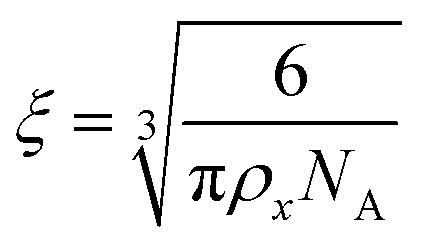
where *N*_A_ is the Avogadro number. The results of the fitting are shown in Table 2 and give a network mesh size varying from ∼20 to 4 nm depending on the gel structure. Such mesh sizes have been observed across different types of self-assembling and polymeric gels of varying cross-link density.^62,65,66^

**Table 2:** Maxwell mode fitting parameters determined from frequency sweep rheometry of 1_RR_ gels using RepTate,^63^ along with cross-link density and network mesh size^a^

Parameter	HH-gel	TF-gel	LC-gel
*G*_1_ (Pa)	103 360	5399	410
*G*_2_ (Pa)	479	1028	40
*G*_3_ (Pa)	5672	1367	561
*G*_4_ (Pa)	58 681	2201	—
*G*_1_ (Pa)	—	13 368	—
*τ*_1_ (s)	335	118	44.0
*τ*_2_ (s)	10.0	8.93	0.321
*τ*_3_ (s)	0.299	0.674	0.00234
*τ*_4_ (s)	0.00895	0.0509	—
*τ*_5_ (s)	—	0.00348	—
*ρ*_*x*_ (mol m^−3^)	71.4	9.92	0.43
*ξ* (nm)	3.5	6.8	19.5

a Where *G*_*i*_ is the modulus of the mode and *τ*_*i*_ the relaxation time of the mode – given by the inverse frequency.

The fitting gives the largest network mesh size, and lowest entanglement density, for the LC-Gel. The smallest network mesh size, and highest entanglement density, arise from the HH-Gel. The LC-Gel is composed of much thinner and shorter fibril bunches (as observed by SEM, Fig. 3d), than the TF- and HH-Gels. This means the fibrils will not only be less stiff than the thicker TF and HH fibres, but also less entangled. This is highlighted by the lower cross-link density and network mesh size predicted by the Maxwell modelling of the frequency sweep data and makes the LC-Gel the weakest of the three systems. This observation ties in with the SANS data in which the Pow_1_ coefficient of 1.7 (*vide supra*) shows a swollen, less entangled gel. The stronger TF-Gel is comprised of much thicker, longer fibrils, and ribbons formed from bundles of the fibrils. The network strength is enhanced because of the increased stiffness of the fibrils and ribbons and the enhanced cross-links.^67^ These form through a combination of branch-points in the ribbons, and physical entanglements because of the length of the fibrils and ribbons (Fig. 3f). The unique structure of the HH fibres produces the most elastic and strong gel, with the highest cross-link density and tightest network mesh size. Given the pathway complexity of the system, gels sonicated before the formation of liquid crystals can form a mix of both the TF and HH fibres. This can give rise to variability in the gel properties, with *G*′ and yield stress varying across two orders of magnitude depending on the exact nature of the gel. Importantly however, the strongest gels contain a high proportion of HH fibres, as observed by SEM. These results show that through control of the pathway complexity of 1_RR_, the fibre and network structure of the resulting gel can be tuned to control properties.

### Circular dichroism analysis of supramolecular polymerization

**Fig. 7:**
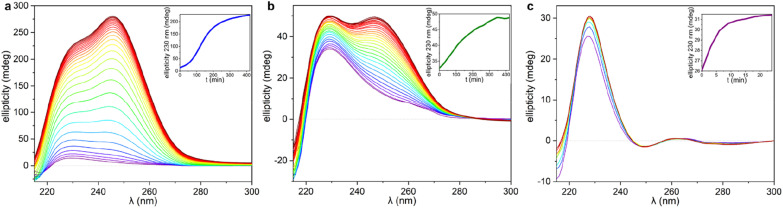
CD spectra recorded during the gelation process. The titration has been plotted following a rainbow pattern with purple as the starting point, and dark red the final stage. The CD spectra of the HH-Gel and TF-Gel were recorded every 5 minutes and plotted every 15 minutes, and LC-Gel CD spectra were recorded and plotted every 3 minutes. In the inserts, the ellipticity at 230 nm as function of time as ‘gelation kinetic profile’ for each gelation are shown. (a) HH-Gel, (b) TF-Gel, (c) LC-Gel.

The significant differences between the three supramolecular polymer fibre morphologies (HH-Gel, TF-Gel and LC-Gel) are indicative of important differences between the self-assembly pathways. The energy barrier for conformational interconversion results in a mixture of species in solution and is likely to be linked to the requirement for a stimulus in the form of ultrasonication to bring about gelation. As described previously for other sonogels,^46^ the fact that the gelation of the HH-Gel and TF-Gel is triggered by ultrasound, might mean that the extra energy provided increases the rate of equilibration between the various conformers and their aggregates effectively increasing the supply of gelling conformer B which is then removed from solution as the gel forms. The sensitivity to temperature during ultrasonication implies that the system exhibits pathway complexity with slower conformational interconversion resulting in a different gel morphology. To examine this issue further, we examined the CD spectra at the early stages of supramolecular polymerization (Fig. S15b†) and monitored their evolution with time (Fig. 7).

As can be seen in Fig. 7 and S15b,† just after applying ultrasound, the ellipticity of 230 nm band associated with the 1D arrangement, is higher than in solution for both sonication temperatures (25 and 70 °C). This behaviour shows that a supramolecular polymerization happens as a result of the ultrasonication. When the stimulus is applied at 70 °C (TF-Gel) the ellipticity at early stages is approximately double than when sonication is performed at room temperature (HH-Gel). The latter indicates that the population of molecules able to self-assemble increases with temperature. This behaviour reinforces the hypothesis that ultrasound cause a conformational change that triggers gelation.

The evolution of the CD profile over time (Fig. 7a and b) shows that the HH-Gel and TF-Gel follow different supramolecular polymerization pathways although the ellipticity of the whole spectrum increases in both cases. The ellipticity of the band related to the 1D supramolecular polymerization at 230 nm was plotted as function of time (inserts of Fig. 7a and b). The evolution of the HH-Gel follows a sigmoidal profile and results in a factor of five greater ellipticity change than in the TF-Gel which exhibits a linear kinetic profile. The considerably greater growth of this band in the HH-Gel relative to the TF-Gel can be related to a more extensive 1D polymerization. The shape of the kinetic profile is also informative of the gelation mechanism.^2,33^ The sigmoidal kinetic profile for the formation the HH-Gel (insert Fig. 6a) shows that it proceeds through an initial slow nucleation stage, followed by a rapid and constant growth phase, and termination step. This gradual evolution of the hyperhelical features is consistent with the SANS data, albeit that it appears to occur on a longer timescale in the SANS measurements. On the other hand, no nucleation stage can be distinguished for the TF-Gel which implying that the higher temperature gives rise to a significant population of molecules of the correct conformation to undergo gelation. The fact that gelation continues spontaneously once it is started by ultrasonication, implies that solution and gel are two kinetically trapped states. When the nucleation energy barrier is overcome, the gelation continues until all molecules in solution above the solubility limit are incorporated to the growing fibres.^68^

The impressive phenomenological differences observed between the gelation processes of LC-Gel and both the HH-Gel and TF-Gel, are also evident in the CD profile of LC-Gel formation over time (Fig. 7c). Plotting the ellipticity of the 230 nm band (supramolecular polymerization related) as function of time, it can be observed that while the HH-Gel and TF-Gel take around 7 hours to complete gelation, the LC-Gel reaches a plateau after less than 10 minutes. The LC-Gel kinetic profile resembles a linear polymerization mechanism without any traces of a nucleation phase.^69^ The fast gelation of the LC-Gel (which is noticeable when the vial is still in the ultrasonic bath) resembles the freezing of over-cooled liquids when a perturbation is applied. The LC droplets seem to be made of partially ordered sections of linear supramolecular polymers of type B conformers self-associated requiring only minimal stimulus to undergo cross-supramolecular crosslinking or entanglement to give a full gel network. These LC droplets thus represents a metastable ‘pre-gel’ state.^70^ When ultrasound is applied, these linear fibrils break and entangle to yield the observed non-helical transparent gel almost instantly.

### Gelation mechanism

Bringing all these observations together allows us to propose a possible mechanism for the pathway complexity in this system which gives rise to three different ‘gelmorphs’. The weak LC-Gels arise from formation of linear fibrils in solution over time to give lyotropic LC droplets which are then sheared and weakly entangled by ultrasound. The very slow growth of the droplets is related to the relatively low population of the gelling conformer B in solution under ambient conditions where C predominates. The stronger TF-Gels are formed when sonicated at elevated temperature (70 °C) where the conformational equilibrium between the three conformers is rapid allowing continuous conversion to the gelling conformer B and its polymerization to give more a cross-linked, defect rich fibre network again with low helicity. The hyper-helical HH-Gel forms at room temperature where conformers A, B and C are in slow equilibrium and DOSY data indicate that at least conformers B and C are aggregated into dimers or short oligomers. Ultrasound is likely to disrupt these oligomers as well as increasing the rate of conformational equilibrium and allow repopulation of conformer B in solution as fibrils begin to nucleate. Even under these conditions, however, nucleation is slow because of the low concentration of conformer B and fibre growth is also likely to be arrested by transient binding of the ‘wrong’ conformer to the end of the growing chain. The presence of the dominant conformer C also provides a mechanism for the formation of the unusual hyper-helical morphology with growth proceeding in a helical manner as a result of association in which the mismatched conformer C binds transiently to one end of the unsymmetrical B conformer. This binding will have a bias toward one face of the growing fibril as a result of the unsymmetrical nature of conformer B and the chirality of the system and hence result in the observed chiral helicity. At higher temperature this binding becomes far less significant resulting in the observed linear tape fibre (TF-Gel) morphology.

## Conclusions

The self-assembly of a small dynamic conformational library results in the emergence of three distinct gelmorphs with very different morphology and rheological properties as a result of pathway complexity in the gels' formation. Gelation of the system is inhibited as a result of the low solution concentration of the folded gelling conformer B. This result contrasts to a recent report on peptide hydrogels in which an extended conformer is responsible for hydrogelation.^71^ In that case the folded conformer hides the hydrophobic sidechain from the hydrophilic solvent. In undisturbed conditions in organic media fibrils based on isomer B form slowly and do not cross-link giving rise to liquid crystalline droplets. Sonication converts these pre-formed fibrils into a weak gel. Application of ultrasound without delay at low temperature allows gives rise to striking hyper-helical gels that are mechanically much stronger. We attribute this complex morphology to transient association of non-gelling conformers during fibre assembly. At higher temperature supramolecular polymerization does not have a nucleation delay with free conversion between conformers and no association of alternate conformers to the end of the growing fibril, resulting in straight tape fibre gels of intermediate strength. This system represents an example of pathway complexity in which complex interactions between slowly equilibrating solution conformers in a supersaturated, non-equilibrium system gives rise to very different, emergent bulk materials properties and serves as a simple test bed for emergent fibrillar assembly phenomena such as amyloid formation.

## Data availability

Underlying data for this work is available at https://doi.org/10.15128/r2sf268517p.

## Author contributions

JWS and JPS conceived the project, JWS supervised the project and obtained funding, JPS first prepared the gelator 1_RR/SS_, MAS and RCM prepared 1_RS_, RCM carried out gel preparation and CD work, SB carried out rheology and XRPD, DSY carried out single crystal crystallography, RCM and JA carried out NMR spectroscopic work, HK and MM carried out the SANS work and contributed to the overall analysis, MR LH and SQ prepared samples and carried out technical work for the SANS measurements, all authors contributed to writing the paper.

## Conflicts of interest

There are no conflicts to declare.

## Supplementary Material

SC-014-D3SC03841F-s001

SC-014-D3SC03841F-s002

## References

[cit1] Sorrenti A., Leira-Iglesias J., Markvoort A. J., de Greef T. F. A., Hermans T. M. (2017). Chem. Soc. Rev..

[cit2] Korevaar P. A., George S. J., Markvoort A. J., Smulders M. M. J., Hilbers P. A. J., Schenning A. P. H. J., De Greef T. F. A., Meijer E. W. (2012). Nature.

[cit3] Hartl F. U., Bracher A., Hayer-Hartl M. (2011). Nature.

[cit4] Ogi S., Grzeszkiewicz C., Würthner F. (2018). Chem. Sci..

[cit5] Robayo-Molina I., Molina-Osorio A. F., Guinane L., Tofail S. A. M., Scanlon M. D. (2021). J. Am. Chem. Soc..

[cit6] Deng J., Walther A. (2020). J. Am. Chem. Soc..

[cit7] Greciano E. E., Matarranz B., Sánchez L. (2018). Angew. Chem., Int. Ed..

[cit8] Kiagus-Armad R., Brizard A., Tang C., Blatchly R., Desbat B., Oda R. (2011). Chem. Eur. J..

[cit9] Li F., Li X. H., Wang Y., Zhang X. (2019). Angew. Chem., Int. Ed..

[cit10] Hendrikse S. I. S., Su L., Hogervorst T. P., Lafleur R. P. M., Lou X., van der Marel G. A., Codee J. D. C., Meijer E. W. (2019). J. Am. Chem. Soc..

[cit11] Scalabre A., Gutierrez-Vilchez A. M., Sastre-Santos A., Fernandez-Lazaro F., Bassani D. M., Oda R. (2020). J. Phys. Chem. C.

[cit12] Khanra P., Singh A. K., Roy L., Das A. (2023). J. Am. Chem. Soc..

[cit13] Borsdorf L., Herkert L., Bäumer N., Rubert L., Soberats B., Korevaar P. A., Bourque C., Gatsogiannis C., Fernández G. (2023). J. Am. Chem. Soc..

[cit14] Morito H., Yamane H. (2010). Angew. Chem., Int. Ed..

[cit15] Singh G., Chan H., Baskin A., Gelman E., Repnin N., Král P., Klajn R. (2014). Science.

[cit16] Brizard A. , Oda R. and Huc I., in Low Molecular Mass Gelators: Design, Self-Assembly, Function, ed. F. Fages, 2005, vol. 256, pp. 167–218

[cit17] Valera J. S., Sánchez-Naya R., Ramírez F. J., Zafra J. L., Gómez R., Casado J., Sánchez L. (2017). Chem. Eur. J..

[cit18] Wehner M., Rohr M. I. S., Buhler M., Stepanenko V., Wagner W., Wurthner F. (2019). J. Am. Chem. Soc..

[cit19] Wagner W., Wehner M., Stepanenko V., Ogi S., Wurthner F. (2017). Angew. Chem., Int. Ed..

[cit20] Ogi S., Stepanenko V., Theirs J., Wurthner F. (2016). J. Am. Chem.
Soc..

[cit21] Ogi S., Stepanenko V., Sugiyasu K., Takeuchi M., Wurthner F. (2015). J. Am. Chem. Soc..

[cit22] Huang X., Raghavan S. R., Terech P., Weiss R. G. (2006). J. Am. Chem. Soc..

[cit23] Andrews J. L., Pearson E., Yufit D. S., Steed J. W., Edkins K. (2018). Cryst. Growth Des..

[cit24] Isare B., Pensec S., Raynal M., Bouteiller L. (2016). C. R. Chim..

[cit25] Fages F. , Vogtle F. and Zinic M., in Low Molecular Mass Gelators: Design, Self-Assembly, Function, ed. F. Fages, 2005, vol. 256, pp. 77–131 10.1007/b107172 22160337

[cit26] Yamanaka M. (2013). J. Inclusion Phenom. Macrocyclic Chem..

[cit27] Wang S., Wu B., Duan J. F., Fang J. L., Chen D. Z. (2014). Prog. Chem..

[cit28] Yokoya M., Kimura S., Yamanaka M. (2021). Chem.–Eur. J..

[cit29] Steed J. W. (2010). Chem. Soc. Rev..

[cit30] Ressouche E., Pensec S., Isare B., Ducouret G., Bouteiller L. (2016). ACS Macro Lett..

[cit31] Isare B., Pensec S., Raynal M., Bouteiller L. (2016). C. R. Chim..

[cit32] Isare B., Pembouong G., Boue F., Bouteiller L. (2012). Langmuir.

[cit33] Baddi S., Madugula S. S., Sarma D. S., Soujanya Y., Palanisamy A. (2016). Langmuir.

[cit34] Deindorfer P., Geiger T., Schollmeyer D., Ye M. H., Zentel R. (2006). J. Mater. Chem..

[cit35] Meziane R., Brehmer M., Maschke U., Zentel R. (2008). Soft Matter.

[cit36] Sravan B., Kamalakar K., Karuna M. S. L., Palanisamy A. (2014). J. Sol-Gel Sci. Technol..

[cit37] Bernstein J., Davis R. E., Shimoni L., Chang N.-L. (1995). Angew Chem. Int. Ed. Engl..

[cit38] Evans L. S., Gale P. A., Light M. E., Quesada R. (2006). New J. Chem..

[cit39] Quinlan E., Matthews S. E., Gunnlaugsson T. (2007). J. Org. Chem..

[cit40] Chu W.-J., Yang Y., Chen C.-F. (2010). Org. Lett..

[cit41] Chu W.-J., Chen J., Chen C.-F., Yang Y., Shuai Z. (2012). J. Org. Chem..

[cit42] Chu W.-J., Chen C.-F. (2012). Tetrahedron.

[cit43] Deindörfer P., Davis R., Zentel R. (2007). Soft Matter.

[cit44] Deindörfer P., Eremin A., Stannarius R., Davis R., Zentel R. (2006). Soft Matter.

[cit45] Himabindu M., Palanisamy A. (2017). Gels.

[cit46] Cravotto G., Cintas P. (2009). Chem. Soc. Rev..

[cit47] Bachl J., Sampedro D., Mayr J., Díaz Díaz D. (2017). Phys. Chem. Chem. Phys..

[cit48] Du X., Zhou J., Shi J., Xu B. (2015). Chem. Rev..

[cit49] Zhang J., Germann M. W. (2011). Biopolymers.

[cit50] Minch M. J. (1994). Concepts Magn. Reson..

[cit51] Gould R. O., Jones C. L., Stephenson T. A., Tocher D. A. (1984). J. Organomet. Chem..

[cit52] Evans R., Dal Poggetto G., Nilsson M., Morris G. A. (2018). Anal. Chem..

[cit53] Heller W. T., Cuneo M., Debeer-Schmitt L., Do C., He L., Heroux L., Littrell K., Pingali S. V., Qian S., Stanley C., Urban V. S., Wu B., Bras W. (2018). J. Appl. Crystallogr..

[cit54] Beaucage G. (1995). J. Appl. Crystallogr..

[cit55] Beaucage G. (1996). J. Appl. Crystallogr..

[cit56] Shen Z., Wang T., Shi L., Tang Z., Liu M. (2015). Chem. Sci..

[cit57] Berova N., Bari L. D., Pescitelli G. (2007). Chem. Soc. Rev..

[cit58] Aparicio F., Matesanz E., Sánchez L. (2012). Chem. Commun..

[cit59] Jeong Y., Hanabusa K., Masunaga H., Akiba I., Miyoshi K., Sakurai S., Sakurai K. (2005). Langmuir.

[cit60] Hirschmann M., Merten C., Thiele C. M. (2021). Soft Matter.

[cit61] Mezger T. G. , The Rheology Handbook, William Andrew Publishing, Norwich, NY, USA, 4th edn, 2012

[cit62] Abrami M., D'Agostino I., Milcovich G., Fiorentino S., Farra R., Asaro F., Lapasin R., Grassi G., Grassi M. (2014). Soft Matter.

[cit63] Boudara V. A. H., Read D. J., Ramírez J. (2020). J. Rheol..

[cit64] Flory P. J. , Principles of Polymer Chemistry, Cornell University Press, Ithaca, USA, 1953

[cit65] Saiani A., Mohammed A., Frielinghaus H., Collins R., Hodson N., Kielty C. M., Sherratt M. J., Miller A. F. (2009). Soft Matter.

[cit66] Lust S. T., Hoogland D., Norman M. D. A., Kerins C., Omar J., Jowett G. M., Yu T. T. L., Yan Z., Xu J. Z., Marciano D., da Silva R. M. P., Dreiss C. A., Lamata P., Shipley R. J., Gentleman E. (2021). ACS Biomater. Sci. Eng..

[cit67] MacKintosh F. C., Käs J., Janmey P. A. (1995). Phys. Rev. Lett..

[cit68] Panja S., Adams D. J. (2021). Chem. Soc. Rev..

[cit69] De Greef T. F. A., Smulders M. M. J., Wolffs M., Schenning A. P. H. J., Sijbesma R. P., Meijer E. W. (2009). Chem. Rev..

[cit70] Piepenbrock M.-O. M., Clarke N., Steed J. W. (2010). Soft Matter.

[cit71] Monti M., Scarel E., Hassanali A., Stener M., Marchesan S. (2023). Chem. Commun..

