# Supplementary material for: Pathway complexity in fibre assembly: from liquid crystals to hyper-helical gelmorphs
Source: Chem Sci. 2023 Sep 26;14(41):11389–401. doi: 10.1039/d3sc03841f (PMC10599479; doi:10.1039/d3sc03841f)
Supplement: SC-014-D3SC03841F-s001 [file SC-014-D3SC03841F-s001.pdf]

Electronic Supplementary Material (ESI) for Chemical Science.
This journal is © The Royal Society of Chemistry 2023

Supporting Information for

Pathway complexity in fiber assembly: from liquid crystals to hyper-helical gels

*Rafael Contreras-Montoya[a], James P. Smith[a], Stephen C. Boothroyd[a], Juan A. Aguilar, Marzieh Mirzamani[b], Martin A. Screen[a], Dmitry S. Yufit[a], Mark Robertson[c], Lilin He[d], Shuo Qian,[d] Harshita Kumari[b], and Jonathan W. Steed[a]**

a) Department of Chemistry, Durham University, Durham DH1 3LE, UK.

b) James L. Winkle College of Pharmacy, University of Cincinnati, 231 Albert Sabin Way, Medical Science Building 3109C, Cincinnati, OH 45267-0514, USA.

c) School of Polymer Science and Engineering, University of Southern Mississippi, 118 College Dr., Hattiesburg, MS, 39406, USA

d) Neutron Scattering Division, Oak Ridge National Laboratory, 1 Bethel Valley Rd., Oak Ridge, TN 37831, USA.

Table of Contents

1. Synthesis……………………………………………………………………………… 3

2. General procedures……………………………………………………………………… 5

3. Gelation screening………………………………………………………………………… 9

4. NMR studies……………………………………………………………………………….. 11

5. Additional figures………………………………………………………………………. 18

6. SANS study………………………………………………………………………………….. 26

Supplementary Section 1. Synthesis

Compound $\mathbf{1_{SS}}$ and $\mathbf{1_{RR}}$

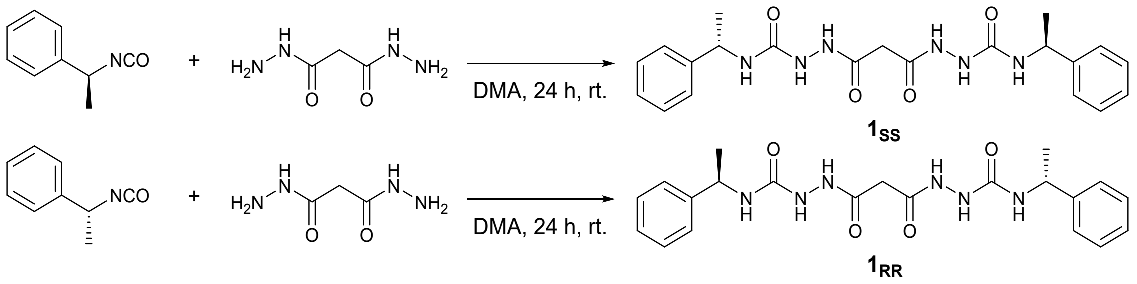

To a of solution of malonic dihydrazide (0.25 g, 1.9 mmol) in dimethylacetamide (DMA) (2 mL), 2.1 equivalents of (*S*)-(-)-α-methylbenzyl isocyanate or (*R*)-(+)-α-methylbenzyl isocyanate (for the synthesis of $\mathbf{1_{SS}}$ or $\mathbf{1_{RR}}$ respectively) were added (0.565 mL, 4.0 mmol) and the mixture was stirred at room temperature for 24 hours. Water was added (100 mL) until a heavy white precipitate appeared. The precipitate was filtered and washed with three portions of water (3 x 50 mL). The solid product was then purified via flash column over silica gel using a mixture 95:5 acetonitrile:water as mobile phase. The purified product was then recovered and redissolved in the minimum amount of ethanol (20 mL). Water was added until the solution turned translucent (250 mL approx.) and then left to precipitate slowly overnight. After vacuum filtration, the final product was obtained as a white powder (0.690 g, 1.6 mmol, 85%). ^{1}H NMR (599.42 MHz, 298.15 K, DMSO-d_6) δ 9.81 (s, 2H), 7.89 (s, 2H), 7.34 – 7.25 (m, 8H), 7.21 – 7.17 (m, 2H), 6.86 (d, J = 8.1 Hz, 2H), 4.77 (p, J = 7.2 Hz, 2H), 3.14 (s, 2H), 1.33 (d, J = 7.0 Hz, 6H). ^{13}C{^{1}H} NMR (150.72 MHz, DMSO-d_6, *298.15 K*) δ 166.66, 157.02, 142.27, 128.11, 126.42, 125.81, 48.58, 40.02, 23.04. **m/z (ES$^+$-MS)** 449.19 [M+Na], 427.21 [M+H]. Anal. calc. for $C_{21}H_{26}N_6O_4$ (%): C, 59.14; H, 6.15; N, 19.71. Found (%): C, 58.89; H, 5.93; N, 19.90.

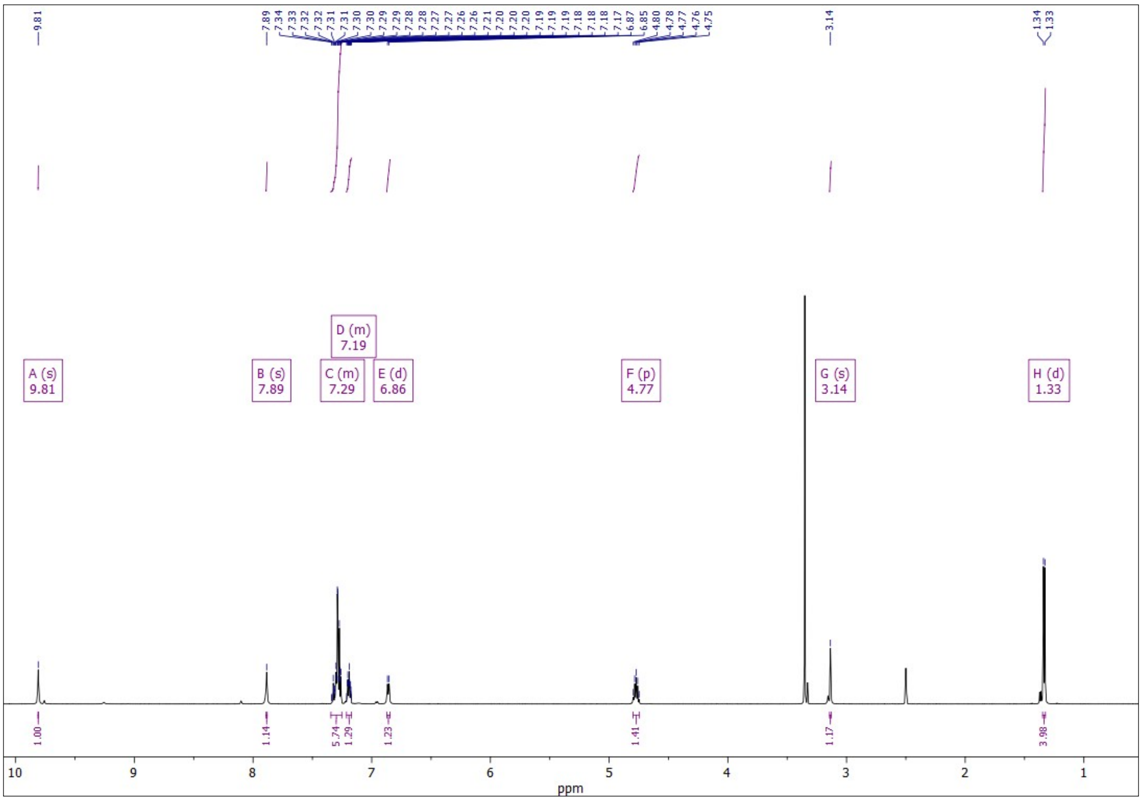

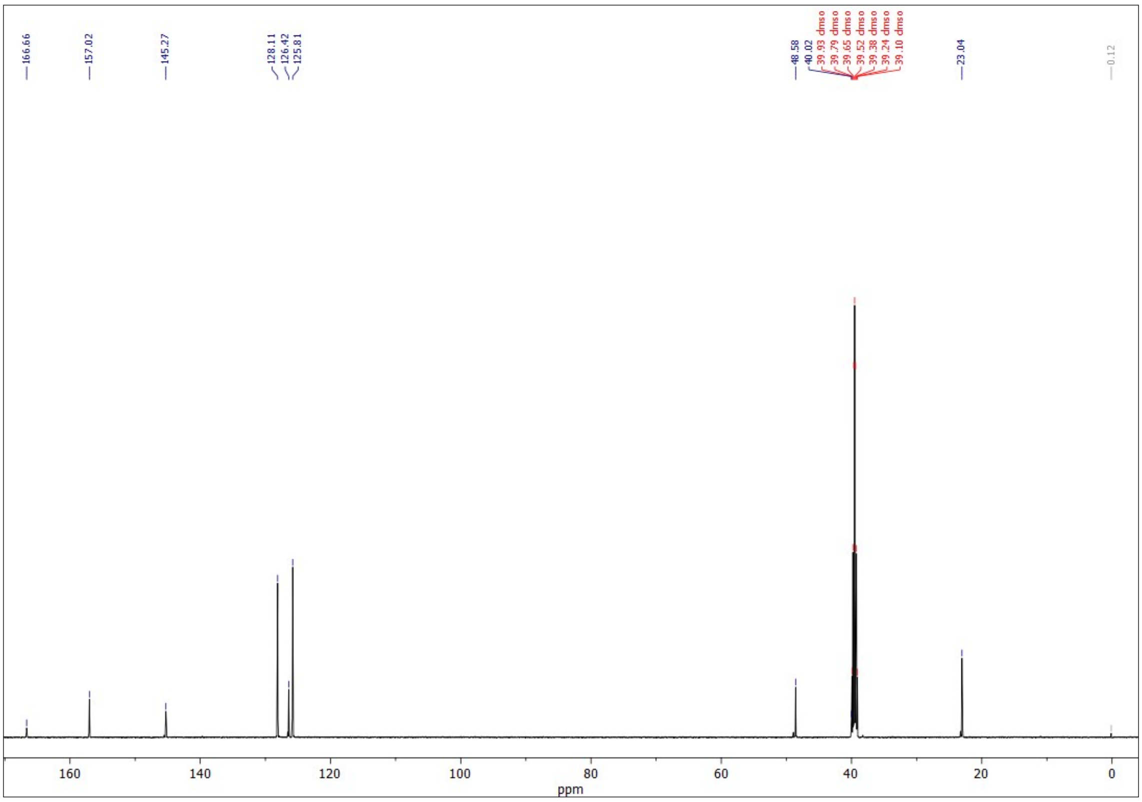

Compound $\boldsymbol{1_{RS}}$

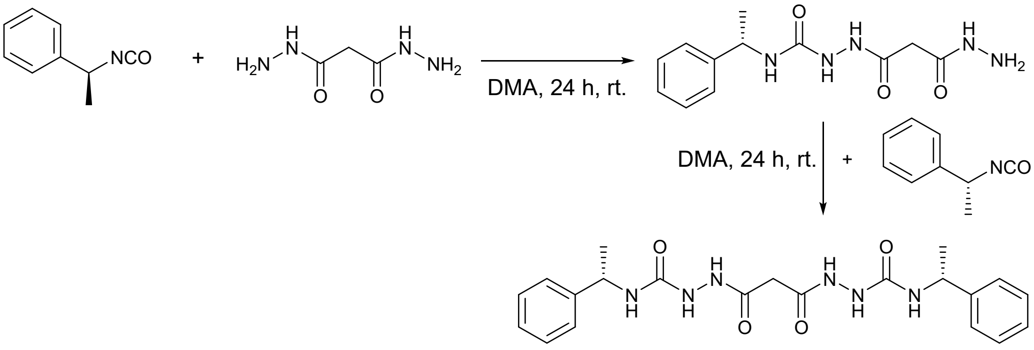

$\mathbf{1_{RS}}$

To a of solution of malonic dihydrazide (0.25 g, 1.9 mmol) in dimethylacetamide (DMA) (2 mL), 1 equivalent of (*S*)-(-)-α-methylbenzyl isocyanate was added (0.267 mL, 1.9 mmol) and the mixture was stirred at room temperature for 24 hours. The solvent was removed *in vacuo* and the residue was purified via flash column over silica gel using a mixtures of acetonitrile:water as mobile phase (from 100% acetonitrile to 95:5). The purified intermediate was dissolved in dimethylacetamide (10 mL) and (*R*)-(+)-α-methylbenzyl isocyanate (0.267 mL, 1.9 mmol) was added to the solution at room temperature, before stirring for 24 hours. Water (200 mL) was added to precipitate the product, which was isolated by vacuum filtration and washed with water (300 mL). The precipitate was purified by column chromatography, eluting dichloromethane:methanol mixtures (from 100% dichloromethane to 95:5) . The purified compound was dissolved in diethyl ether and the solvent was removed *in vacuo* to obtain 2,2'-malonylbis(N-((R,S)-1-phenylethyl)hydrazine-1-carboxamide) as a white powder (0.45 mmol, 12% yield). ^{1}H NMR (400 MHz, DMSO-d_6) δ 9.83 (d, J = 2.0 Hz, 2H, NH-NH-CO-CH_2), 7.93 – 7.88 (m, 2H, NH-CO-NH-NH), 7.35 – 7.23 (m, 8H, Ar-H), 7.26 – 7.15 (m, 2H, Ar-H), 6.88 (d, J = 8.2 Hz, 2H, CH-NH-CO-NH), 4.77 (dq, J = 7.2, 8.2 Hz, 2H, Ph-CH-NH), 3.14 (s, 2H, CO-CH_2-CO), 1.33 (d, J = 7.2 Hz, 6H, CH-CH_3). ^{13}C{^{1}H} NMR (100.6 MHz, DMSO-d_6) δ 167.16, 157.49, 145.74, 128.69,

128.58, 126.88, 126.27, 49.02, 23.53. m/z (ESI-MS) 449.49 [M+Na], 427.48 [M+H], calculated exact mass 426.20 g mol^{-1}.

Supplementary Section 2: General procedures

Crystallization.

Structure of $\mathbf{1_{RR}}$: 5 mg of $\mathbf{1_{RR}}$ was dissolved in 1 mL ethanol. Water was added in 50 µL aliquots until the solution turned translucent (0.8 mL total). The solution was then heated at 75 ºC until turned transparent, before being cooled slowly from 75 ºC to 50 ºC over 10 days. This yielded crystalline needles which were identified to be an ethanol solvate of $\mathbf{1_{RR}}$.

*Structure of $\mathbf{1_{RR}}$-$\mathbf{1_{SS}}$ racemic mixture acetonitrile solvate (stable conformer **A** and metastable conformer **B**):* 1.5 mg of 1:1 $\mathbf{1_{SS}}$ and $\mathbf{1_{RR}}$ was dissolved in 2 mL with heat and slowly cooled from 80 ºC to 50 ºC over 5 days during which time a concomitant mixture of needle and plate crystals formed. Crystals were stored at room temperature in the mother liquor thereafter. The needle crystals were a stable acetonitrile solvate of $\mathbf{1_{RR}}$-$\mathbf{1_{SS}}$ racemic mixture in conformation A while the plates were unstable and decomposed in the synchrotron beam. The structure obtained is an acetonitrile solvate of $\mathbf{1_{RR}}$-$\mathbf{1_{SS}}$ racemic mixture in conformation **B**.

Structure of $\mathbf{1_{RR}}$ dihydrate: $\mathbf{1_{RR}}$ (10 mg) was dissolved in 3 mL of a 1:1 1,4-dioxane/water mixture using heat. The solution allowed to cool to ambient temperature and left undisturbed for 5 days, after which crystalline needles had formed.

Structure of $\mathbf{1_{RR}}$ ethanolate: $\mathbf{1_{RR}}$ (5 mg) was dissolved in 1 mL EtOH and 3 × 0.5 mL aliquots of water (1.5 mL total) was added to the vial. The resulting translucent solution was then heated until transparent and allowed to cool to room temperature. The vial was left undisturbed for 3 months after which time small crystalline needles had formed.

Structure of $\mathbf{1_{RS}}$: $\mathbf{1_{RS}}$ (5 mg) was dissolved in 1 mL EtOH and 3 × 0.5 mL aliquots of water (1.5 mL total) was added to the vial. The resulting translucent solution was then heated until transparent and allowed to cool to room temperature. The vial was left undisturbed for three days after which time plate shaped crystals had formed.

X-ray diffraction.

The X-ray single crystal data for compounds $\mathbf{1_{RS}}$, and $\mathbf{1_{RR}}$·EtOH were collected at temperature 120.0(2)K using MoKα radiation (λ = 0.71073Å) on a Bruker D8Venture (Photon100 CMOS detector, IµS-microsource, focusing mirrors) 3-circle diffractometer equipped with a Cryostream (Oxford Cryosystems) open-flow nitrogen cryostat. Single crystal data for compounds $\mathbf{1_{RR}}$·2H_2O, $\mathbf{1_{RR}}$·2MeCN (Form A) and $\mathbf{1_{RR}}$.2MeCN (Form B) were collected at 100.0(2)K at I-19 beamline (Dectris Pilatus 2M pixel-array photon-counting detector, undulator, graphite monochromator, λ = 0.6889 Å) of the Diamond Light Source, Oxfordshire UK, and processed using Bruker APEXIII software. The weakly diffracting tiny crystals of $\mathbf{1_{RR}}$.2MeCN Form B decayed rapidly in the synchrotron beam and the dataset used was obtained by combining reflections from short preliminary runs for only two available crystals. The data is of low quality and low completeness, but the molecular structure and conformation is unambiguous.

All structures were solved by direct methods and refined by full-matrix least squares on F^2 for all data using Olex2[1] and SHELXTL.[2] All non-hydrogen atoms were refined in anisotropic approximation, hydrogen atoms were placed in the calculated positions and refined in riding mode. Atoms of ethanol solvent molecule in structure $\mathbf{1_{RR}}$·EtOH were refined with fixed SOF=0.5. Crystal data and parameters of refinement are listed in Table S1. Crystallographic data has been deposited with the Cambridge Crystallographic Data Centre as supplementary publication CCDC 2222820 – 2222824.

Table S1. Crystal data and structure refinement parameters for the studied compounds

Identification code	**1**$_{\mathbf{RR}}$·2H$_2$O	**1**$_{\mathbf{RR}}$·2MeCN (A)	**1**$_{\mathbf{RR}}$·2MeCN (B)	**1**$_{\mathbf{RS}}$	**1**$_{\mathbf{RR}}$·EtOH
Empirical formula	$C_{21}H_{26}N_6O_4$ x 2 H_2O	$C_{25}H_{32}N_8O_4$	$C_{25}H_{32}N_8O_4$	$C_{21}H_{26}N_6O_4$	$C_{22}H_{29}N_6O_{4.5}$
Formula weight	462.51	508.58	508.58	426.48	449.51
Temperature/K	100.0	100.0	100.00	120.0	120.00
Crystal system	monoclinic	monoclinic	monoclinic	monoclinic	monoclinic
Space group	*P*2$_1$	*C*2/*c*	*P*2$_1$/*c*	*P*2$_1$/*c*	*P*2$_1$
a/Å	11.300(3)	38.366(10)	18.482(4)	14.3484(8)	11.278(2)
b/Å	4.5408(11)	4.5756(11)	9.1899(15)	9.2167(5)	4.5289(10)
c/Å	21.909(5)	15.248(4)	17.264(3)	17.5718(9)	22.111(5)
α/°	90	90	90	90	90
β/°	90.897(7)	99.235(7)	108.867(4)	110.049(2)	91.188(5)
γ/°	90	90	90	90	90
Volume/Å^3	1124.1(5)	2642.1(11)	2774.6(9)	2183.0(2)	1129.1(4)
Z	2	4	4	4	2
ρ_{calc}g/cm^3	1.366	1.279	1.217	1.298	1.322
μ/mm^{-1}	0.096	0.085	0.081	0.093	0.095
F(000)	492.0	1080.0	1080.0	904.0	478.0
Radiation	Synchrotron (λ = 0.6889)	Synchrotron (λ = 0.6889)	Synchrotron (λ = 0.6889)	MoKα (λ = 0.71073)	MoKα (λ = 0.71073)
Reflections collected	11700	21296	12165	25558	19132

Independent refl., R_{int}	5176, 0.0846	3862, 0.0808	3634, 0.1357	5792, 0.0573	4432, 0.2458
Data/restraints/parameters	5176/1/307	3862/3/182	3634/54/315	5792/0/306	4432/19/311
Goodness-of-fit on F^2	1.034	1.073	1.285	1.066	0.932
Final R_1 [$I \geq 2\sigma$ (I)]	0.1179	0.0679	0.1509	0.0575	0.0792
Final wR_2 [all data]	0.3656	0.2036	0.4319	0.1256	0.1785
Largest peak/hole/e $Å^{-3}$	0.34/-0.38	0.49/-0.32	0.69/-0.54	0.25/-0.28	0.24/-0.28
Flack parameter	-1.8(10)	n/a	n/a	n/a	-2.4(10)

Materials preparation.

***HH-gel** and **TF-gel**:* typically, 0.5 mL of 1 %wt. solution of $\mathbf{1_{RR}}$ or $\mathbf{1_{SS}}$ in 1,4-dioxane was prepared in a glass vial heating a gelator suspension for 5 minutes at 95 ºC in a heating bath. The solution was then thermostatted at 25 ºC or 70 ºC (for HH-gel or TF-gel respectively) soaking the vial in the turned off ultrasound bath for 5 minutes. Then, ultrasound (ultrasonic cleaner bath VWR USC 300 THD/HF) was applied for 60 seconds at 25 ºC or 70 ºC (for HH-gel or TF-gel respectively), and it was left still to gel at room temperature for at least 12 hours.

LC droplets: the clear solution of $\mathbf{1_{RR}}$ or $\mathbf{1_{SS}}$ prepared as above was left still at room temperature for one week. The LC droplets started to be visible to the naked eye after two days. When the LC droplets were required for further experiment, they were taken by detaching them from the walls of the vials with a cat whisker and cautiously removing the mother liquors.

***LC-gel**:* the LC droplets suspension prepared above was sonicated at 25 ºC for 10 seconds and left still to gel for at least 1 hour.

Circular Dichroism (CD) and absorbance.

The CD and absorbance spectra were simultaneously recorded using a Jasco J-810 spectropolarimeter. Spectra were obtained at 20 ºC from 215 to 300 nm with a 1 nm step, 8 seconds of digital integration time, 5 nm of bandwidth and a scan speed of 50 $nm \cdot min^{-1}$. All spectra obtained were treated subtracting the spectra of pure 1,4-dioxane measured using the same cuvette and conditions, and the. The gelation solutions were prepared in glass vials using the protocols described above and were injected inside 0.1 mm path length quartz cells (Hellma 0.1 mm quartz Suprasils(R)) immediately after sonication. For kinetic experiments, the gelation solutions were introduced in the cuvette already placed in the spectropolarimeter and were left to thermostatize for two minutes before recording spectra every three minutes for LC-gel gelation and every five minutes for the gelation of HH-gel and TF-gel. Except for kinetic experiments, all results are expressed as the average of three accumulations. The baseline of CD spectra was normalized by subtracting the ellipticity measured at 300 nm of the first scan where no absorbance was detected. The HT measured for all the experiments was lower than 400 V at wavelengths higher than 220 nm.

SEM images.

To prepare SEM samples of xerogel dried to the atmosphere, small gel portions were taken with a spatula and put on silicon wafers, left to dry for 1 day and coated with 3 nm of platinum using a Cressington 328 Ultra High-Resolution EM Coating System. The SEM samples of freeze-dried xerogels were prepared freeze-drying the gel, taking a portion with a spatula and put on an adhesive surface, and coated with 10 – 13 nm of gold using a Cressington 108 Auto sputter coater. The images were obtained using a Carl Zeiss Sigma 300 VP FEG SEM microscope, operated at 5 kV using an inlens detector.

Rheology.

Rheometry was conducted on a TA Instruments AR2000 rheometer equipped with a peltier plate, using a 25mm rough top plate geometry. To avoid damage to the formed gel, samples were placed in a well plate on the rheometer immediately after sonication and left to gel overnight at 20 °C, covered with a sealed watch glass to avoid solvent evaporation. Measurements were then conducted at a temperature of 10 °C to limit solvent evaporation. Stress sweeps were conducted to determine the limit of the linear viscoelastic region (LVR) and yield stress, at a frequency of 10 rad s^{-1}. Frequency sweeps were conducted at an appropriate stress within the LVR. The yield stress was given by the end of the linear viscoelastic region, calculated as the point of continuous reduction in G' of over 5% from a 5-point moving average of the preceding points.

A generalized Maxwell model[3] was fitted to the frequency sweep data[4] in RepTate:[5]

$$G'(\omega) = \sum_{1}^{n} G_i \frac{(\omega\tau_i)^2}{1 + (\omega\tau_i)^2} \quad (1)$$

$$G''(\omega) = \sum_{1}^{n} G_i \frac{\omega\tau_i}{1 + (\omega\tau_i)^2} \quad (2)$$

Where n is the number of Maxwell modes used to fit the data, equally distributed across a logarithmic scale between $\omega_{min}$and ω_{max}. G_i is the modulus of the mode, and τ_i the characteristic relaxation time of the mode (given by the inverse of the frequency). The number of modes used was determined by minimising the product of the sum of the squared errors, χ^2, and the number of fitting parameters: $\chi^2 \times (2+n)$. The elastic shear modulus, G, is determined by summing G_i.

Small-Angle Neutron Scattering (SANS).

Each gel sample was prepared as described above, except immediately after the sonication step the sample was pipetted into a titanium demountable cell outfitted with quartz windows and then loaded into the instrument to begin data collection. The elapsed time between the end of the sonication step and the beginning of each scan for each gel is shown in Table S5 in Supplementary Section 6 below. SANS was conducted on the GP-SANS instrument in the High Flux Isotope Reactor at Oak Ridge National Laboratory (Oak Ridge, TN, USA). Three instrument configurations were used to obtain a full q-range of 0.0015 $Å^{-1} < q < 0.81$ $Å^{-1}$, where $q = (4\pi/\lambda)\sin(\theta/2)$ and is the scattering vector, λ is the neutron wavelength, and θ is the scattering angle. 1) a sample-to-detector distance (SDD) of 19 m and λ=12 Å for the low-q data, 2) a SDD of 7 m and λ=4.75 Å for intermediate-q data, and 3) a SDD of 1 m and λ=4.75 Å for the high-q data. The wavelength spread $\Delta\lambda/\lambda$ = 13% for all configurations. To obtain good scattering statistics, the low-q data was collected for 30 min, mid-q data was collected for 20 min, and high-q data collection was 10 min. Jupyter Notebook developed by ORNL was used to reduce the data. All measurements were conducted at room temperature (22°C), and the scattering intensities were calibrated by using porous silica as a secondary standard. The data were corrected for empty cell scattering, sample transmission, sample thickness, and detector sensitivity. The isotropic two-dimensional (2D) scattering patterns were then azimuthally reduced to one-dimensional (1D) intensity, which was then modeled using a combination of Power Law + Unified Exponential/Power Law fit models using the NIST macros developed for Igor Pro (WaveMetrics, Portland, OR, USA).[6-7]

Supplementary Section 3: Gelation screening

Procedure: In small glass vials, 5 mg of solid gelator were weighted and 0.5 mL of the desired solvent were added to reach a 1% (w/v) final concentration. In case the gelator was soluble, the solution was left still for one week observing its evolution. In case the gelator was insoluble at room temperature, heat was applied until the solvent boiled. If the solid could not be dissolved by heating, that solvent was discarded for further experiments, but in case it was soluble, the solution evolution was observed for a week. All solvents where gels appeared after obtaining the solution were discarder for further experiments. If no gels were obtained after one week, the experiments were repeated sonicating the freshly prepared solution. In case gels were obtained (sonogel), no further experiments were performed. In case a precipitate appeared in a solvent after just dissolving, or dissolving and sonicating, that solvent was discarded. In all cases where no gel or precipitate was observed, the experiments were repeated increasing the gelator concentration to

2% (w/v) and if after that no changes were observed, it was considered that the gelator is soluble in that solvent. All results obtained are summarized in Table S2.

Table S2. Full gelation screening of $\mathbf{1_{RR}}$ and $\mathbf{1_{SS}}$ at 1% (w/v) in a range of solvents. IS = insoluble, X = crystallisation, P = precipitation, S = soluble, PS = partially soluble, SH = soluble only when heated, HG = heterogeneous gel, G^O = opaque gel, G^T = translucent gel, G^C = transparent/clear gel. An asterisk indicates where sonication was required for gelation to occur. The symbol † indicates where a result obtained at 2% (w/v).

**Solvent**	$\mathbf{1_{RR}}$ **or** $\mathbf{1_{SS}}$	$\mathbf{1_{RS}}$	**Solvent**	$\mathbf{1_{RR}}$ **or** $\mathbf{1_{SS}}$	$\mathbf{1_{RS}}$
Acetone	G^O*	IS	Ethyl acetate	P	-
Acetonitrile	G^T*	P	Ethylene glycol	S	-
Aniline	G^T*	IS	Mesitylene	HG†	-
Benzyl alcohol	S	PS	Methanol	S	-
1-butanol	G^O*	-	4-methylmorpholine	SH	-
2-butanol	G^O*	P	Morpholine	S	-
2-butanone	P	IS	Nitrobenzene	G^C	P
Chlorobenzene	G^T	-	Nitromethane	G^O*	P
Chloroform	P	-	o-xylene	HG†	-
Cyclohexane	P	-	1-pentanol	G^T*	P
Cyclohexanone	P	-	2-pentanol	G^T*	-
Cyclopentanone	P	-	2-picoline	S	-
1,2-dichlorobenzene	G^T	P	3-picoline	S	-
1,3-dichlorobenzene	G^T	P	4-picoline	S	-
Dichloromethane	P	-	1-propanol	G^T*	P
Diethyl ether	IS	-	2-propanol	G^T*	P
Dimethyl acetamide	S	-	Pyridine	S	S
Dimethylformamide	S	-	Tetrahydrofuran	G^O*	P
Dimethyl sulfoxide	S	-	Toluene	G^T	-
Dimethoxymethane	IS	-	1,3,4-trichlorobenzene	G^T	P
1,4-dioxane	G^O*	P	Water	G^T*	P
Ethanol	S	-			

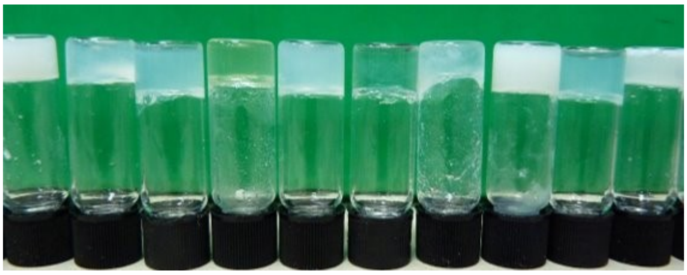

Figure S1. Photograph showing a selection of the 1% (w/v) gels formed by dissolving $\mathbf{1_{SS}}$ in different solvents, (left to right) 2-butanone, 1-pentanol, cyclohexanone, 1,3-dichlorobenzene, chlorobenzene, 1,4-dioxane, acetonitrile, acetone, ethanol and methanol.

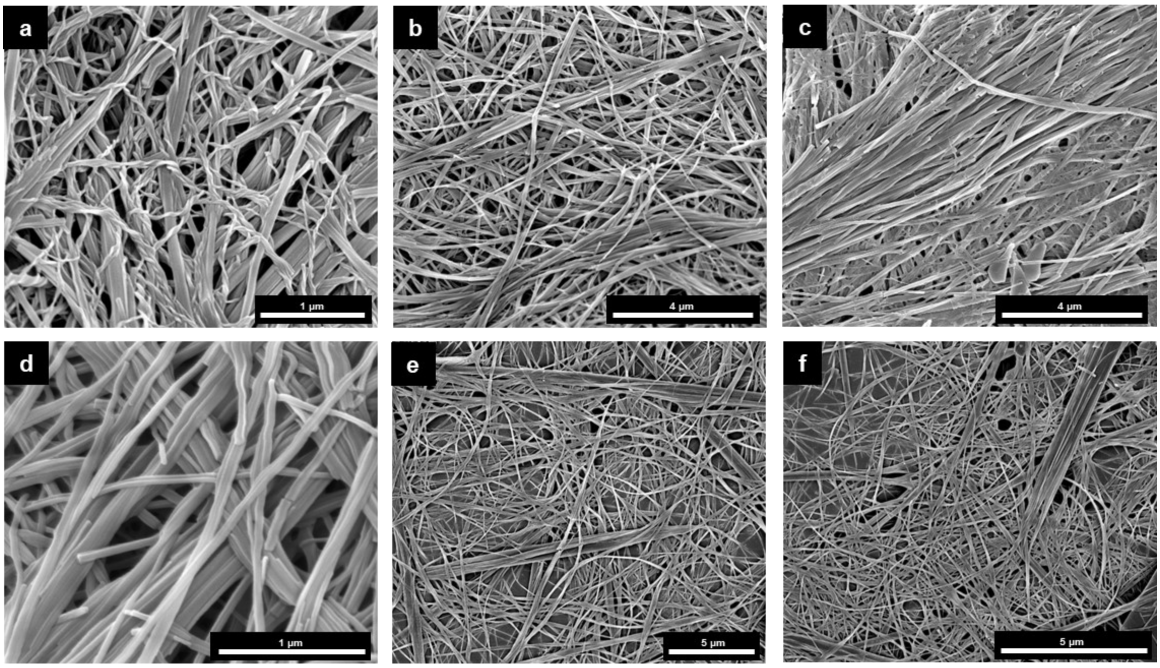

Figure S2. SEM pictures of a selection of 1% (w/v) gels formed by dissolving 1_{SS} in different solvents dried to air. (a) Acetone. (b) 1-butanol. (c) 1,2-dichlorobenzene. (d) Tetrahydrofuran. (e) 1-propanol. (f) Nitrobenzene.

Supplementary Section 4: NMR studies

All Nuclear Magnetic Resonance (NMR) spectra were recorded, unless otherwise stated, at 25 ^{0}C, on a Varian VNMRS-600 spectrometer equipped with an Agilent OneNMR Probe able to deliver a maximum pulsed field gradient of 62 G cm^{-1}, with operating frequencies of 599.42 MHz for **^{1}H**, and 150.72 MHz for **^{13}C**. Variable temperature ^{1}H-NMR spectra were recorded on a Varian DD2-500 spectrometer operating at of 499.53 MHz for **^{1}H**.

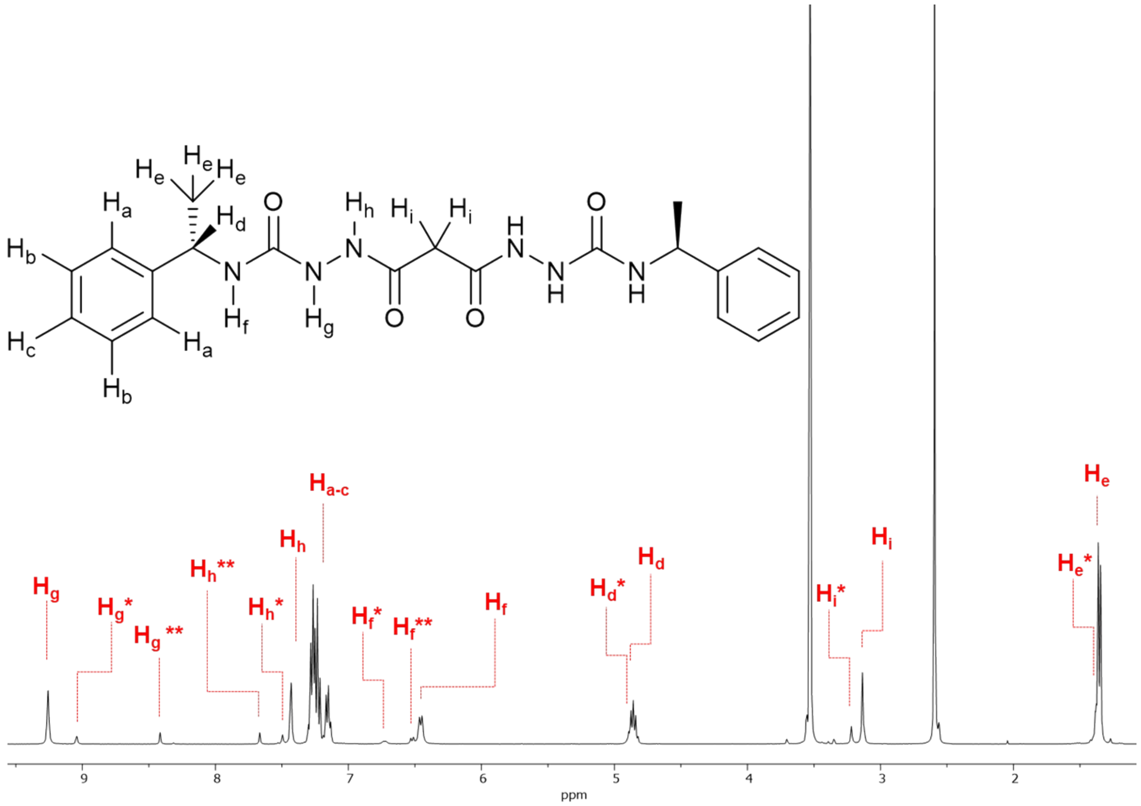

Figure S3. 600 MHz ^{1}H spectrum of 1_{RR} in 1,4-dioxane-d_8 at 25 ºC.

The spectrum of $\mathbf{1_{RR}}$ in 1,4-dioxane-d_8 (Figure S1) shows two sets of signals belonging to several conformers with unequal populations (Table S3). The more abundant minor conformer is labelled with * and ** and the major one without asterisks.

Table S3. ^{1}H peak list in alphabetical order:

Major conformer:	*Minor conformers:*
A: 7.27 ppm	
B: 7.23 ppm	
C: 7.15 ppm	
D: 4.86 ppm	*D**: 4.84 ppm
E: 1.36 ppm	*E**: 1.38 ppm
F: 6.41 ppm	*F**: 6.68 ppm; *F***: 6.47 ppm
G: 9.21 ppm	*G**: 9.01 ppm; *G***: 8.39 ppm
H: 7.41 ppm	*H**: 7.47 ppm; *H***: 7.64 ppm
I: 3.13 ppm	*I**: 3.22 ppm

Variable Temperature NMR (VT-NMR).

To prove that the presence of extra signals is due to the presence of interconverting conformers, we conducted a variable temperature study expecting the minor and major conformers' signals to merge at high temperatures, as this is what we observed (Figure S4a). The process is reversible, as can be seen comparing the first ^{1}H spectrum acquired at 25 ºC with another spectrum acquired after the 85 ºC sample was let to cool down to 25 ºC (Figure S4b).

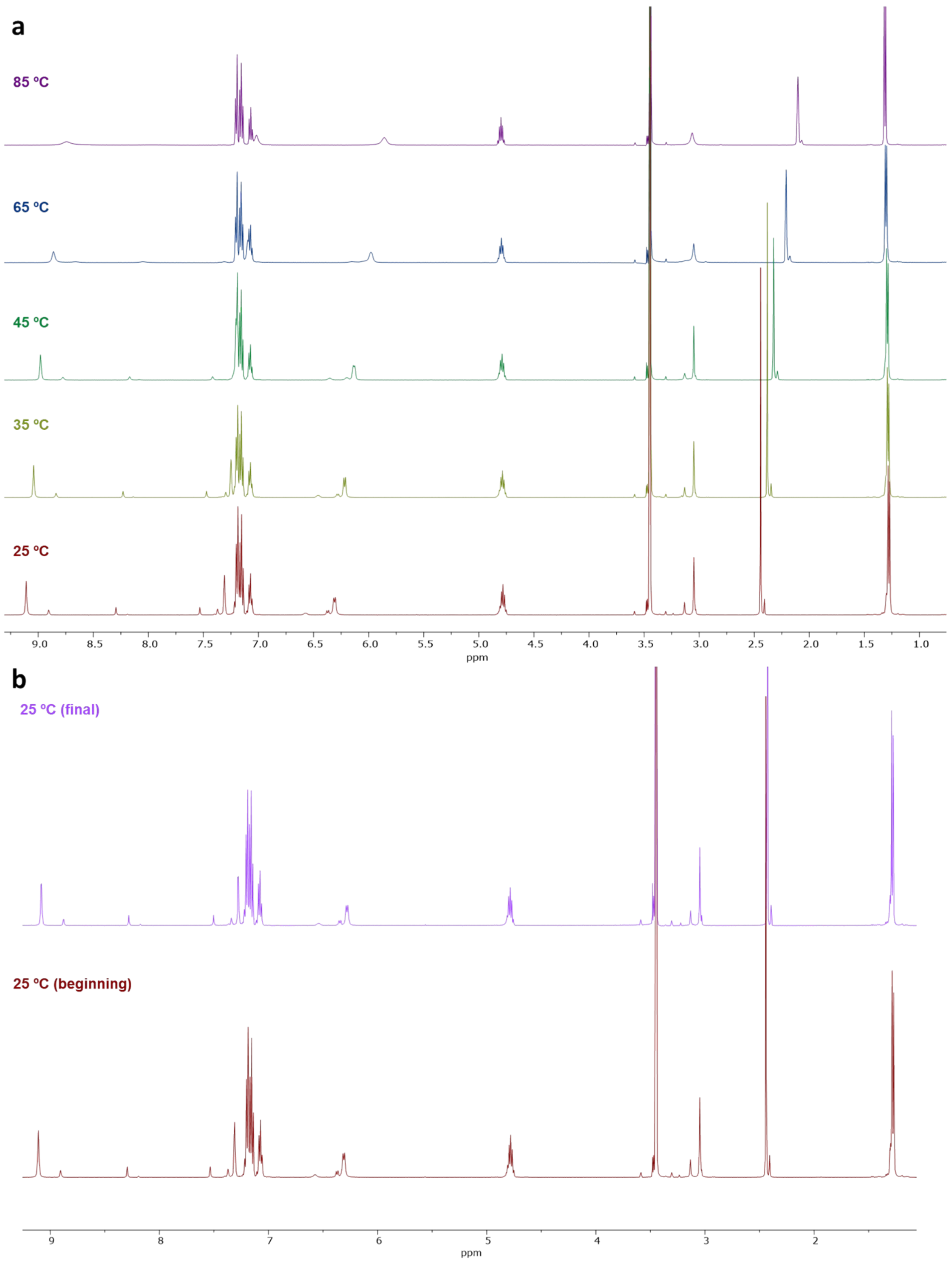

Figure S4. Variable temperature NMR. (a) ^{1}H NMR spectra acquired at temperatures ranging from 25 to 85 ºC. Increasing the temperature increases the frequency of the interconversion between conformers such that only an average conformation is seen at 85 ºC. The process is reversible, as proven by comparing the spectrum acquired at 25 ºC before heating up the sample and after cooling it down to the same temperature (b).

EASY-ROESY

While the variable temperature suggest that the minor and major conformers interconvert, this is not a strict proof of conformer interconversion. An EXSY run in the form of an NOESY or a

ROESY can indeed prove that the minor and major sets of signals interconvert, and it can identify the pairs or interconverting signals. Conformational and proximity effects cannot be separated using a NOESY (see below) when the correlation time is smaller than the Larmor frequency, as it was the case since the nOe cross-peaks showed the same phase as the diagonal (Main text Figure 4); however, these effects can be separated using a ROESY (an EASY-ROESY in this case). In a ROESY, cross-peaks due to exchange (chemical or conformational) have the same phase as diagonal peaks; cross-peaks due to TOCSY effects (due to scalar couplings) have the same phase as the diagonal. Here contributions due to scalar couplings were identified using a COSY as a reference (this cannot be done using TOCSY as a reference). The ROESY spectra (Figure S5) was employed to discern which cross-peaks in NOESY were due to proximity.

EASY-ROESY experimental conditions: four transients, 128 steady state transients, and 2,048 complex data points covering 6 kHz were used in both dimensions. Five hundred and twelve complex points were acquired to sample the indirect dimension. The mixing time was 200 ms. The tilt angle was 50.01. The repetition time was 1.68 s, of which 0.68 s comprised the acquisition time.

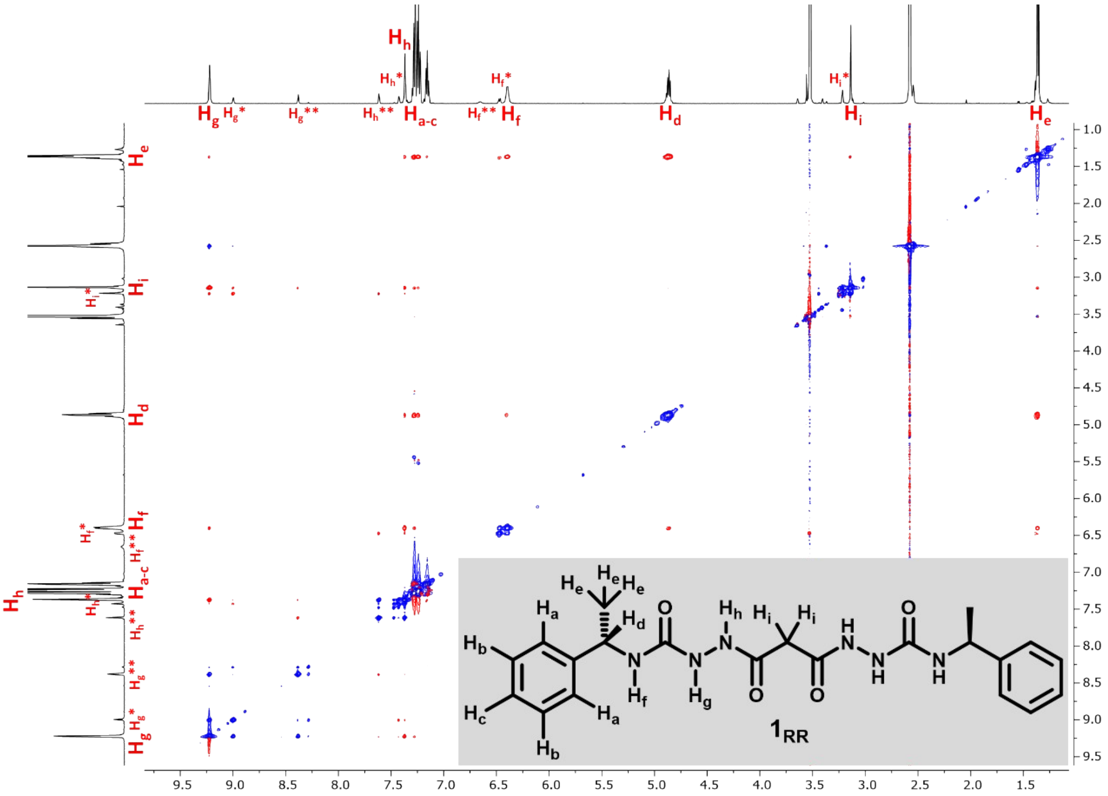

Figure S5. 600 MHz EASY-ROESY[8] of 1_{RR} in 1,4-dioxane-d_8. Cross-peaks due to spatial proximity (rOe, red peaks) have the opposite sign than the diagonal (blue peaks). Cross-peaks resulting from exchange or TOCSY have the same sign than the diagonal (blue peaks).

Estimating inter-proton distances using NMR

Once the exchange contribution was identified and discarded, we used proximity peaks (nOe) in the NOESY spectrum to roughly estimate inter-proton distances (Main text Figure 4). The integral of a nOe cross-peak is proportional to $1/d^{-6}$ where d is the distance between two protons. The nOe cross-peak integral between two peak whose distance is known can be used to estimate the proportionality constant.

We used the cross peak between H_b-H_c for this purpose (the distance is estimated to be 2.340 Å). In passing we note that this nOe cross-peak coincides with an anti-phase COSY peak, as is usual in these cases; however, the integral of the COSY cross peak should be close to zero, as the signal

has an anti-phase character. Nonetheless, distance estimations obtained using NOESY peaks should be used in addition to other evidence, as NMR detects contributions (or the lack of) from the ensemble of conformations in solution, especially when molecules are not rigid.

NOESY Experimental conditions: Four transients, 32 steady state transients, and 2,610 complex data points covering 6.1 kHz were used in both dimensions. Five hundred and twelve complex points were acquired to sample the indirect dimension. The mixing time was 300 ms. The repetition time was 3.4 s, of which 0.4 s comprised the acquisition time. COSY cross-peaks were attenuated using the Keeler-Pell zero-quantum filter.

The main nOe cross-peaks are detailed in Table S4. Every cross-peak has been compared with the two different conformations observed from the single crystal X-ray diffraction (SC-XRD) structures (Figure S4). In conformation **A**, both halves of the molecule are equivalent, but this does not happen in the bent conformation **B**. To make the analysis simpler, an arbitrary representation of the molecule has been chosen to assign labels to the hydrogen atoms. In this representation, two halves with different labels can be distinguished: ‘left’ and ‘right’ sides (see Figure S4).

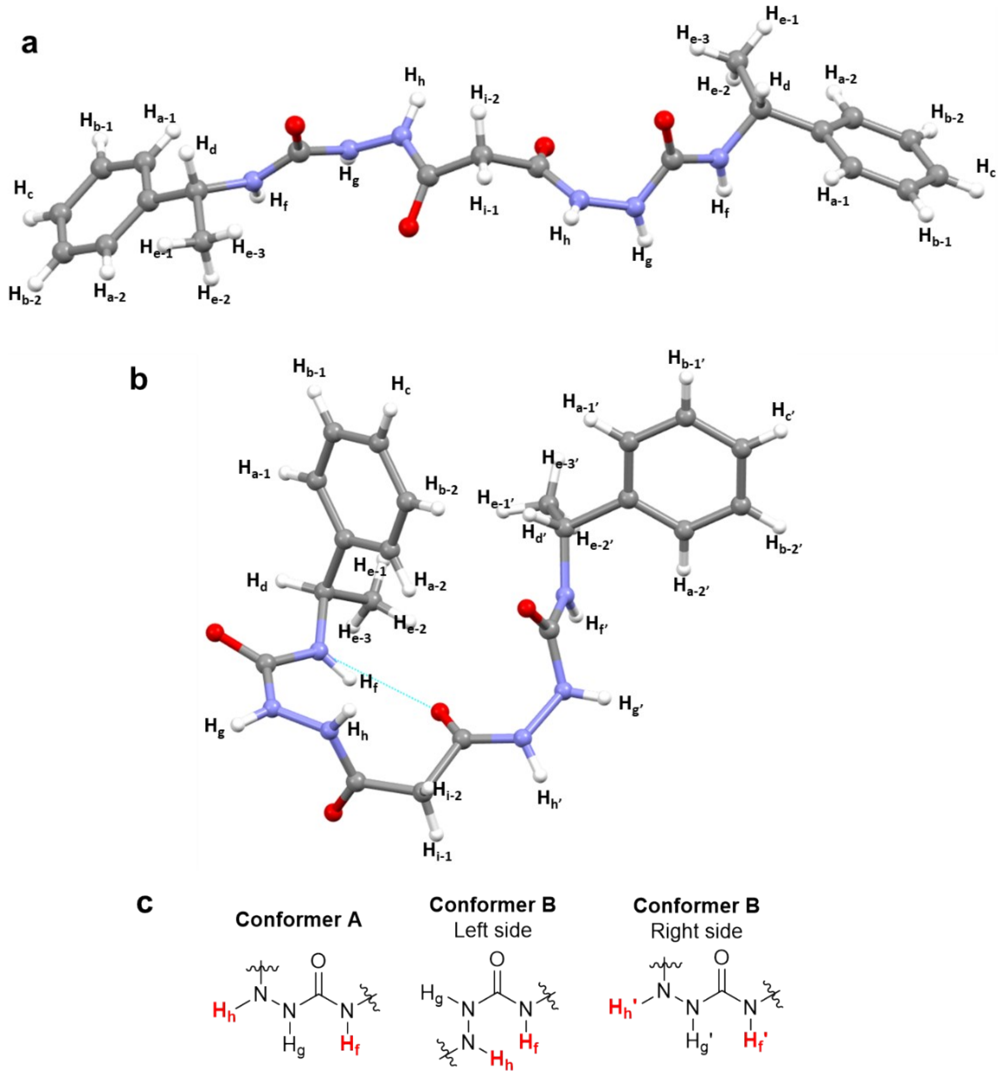

Figure S6. (a) Labelled representation of the linear conformation **A** of $\mathbf{1_{RR}}$ obtained from the diffraction of needle-shape ethanol solvate crystals. (b) labelled representation of the bent conformation **B** obtained from the diffraction of plate-shape acetonitrile solvate racemic crystals. In the representation, an arbitrary distinction between both sides of the methylene group (‘left’ and ‘right’ sides) has been done using different labels for the hydrogen atoms. (c) Schematic representation of the different possible configurations of a

part of the molecule for the **B** (left and right sides) and the **A** conformations. Protons H_h and H_f have been highlighted in red.

Table S4. List of nOe cross-peaks with their estimated distances (see text) between protons in Å. A colour code has been used to indicate whether the estimated distance could have been produced by a particular conformer: light green - likely; turquoise - 'not impossible'; red - 'unlikely'; light blue - uninformative or trivial cross-peaks.

nOe*	Estimated from integrals	linear-SC	bent-SC (left side)	bent-SC (right side)
H_a-H_e (weak)	4.5	trivial	trivial	trivial
H_a-H_i (weak)	4.5	Too far	Too far	Too far
H_d-H_e	3.2	trivial	trivial	trivial
H_d-H_f	3.3	2.7 (anti)	2.8 (anti)	2.7(anti)
H_d-H_g (weak)	4.2	4.3 (anti)	4.5 (syn)	4.2 (anti)
H_d-H_h (weak)	3.7	5.4 (syn)	5.0 (anti)	5.7 (anti)
H_d-H_i (weak)	4.5	Too far	Too far	Too far
H_e-H_f	3.3	3.0 (syn-anti)	2.3, 2.8 (syn)	2.8, 2.8 (syn)
H_e-H_g (weak)	4.5	4.7 (anti)	5.5 (anti)	4.8 (syn)
H_e-H_h (weak)	4.2	5.4 (anti)	4.8 (syn)	6.5 (anti)
H_e-H_i (weak)	4.2	Too far	Too far	Too far
H_f-H_g	3.6	2.2 (syn)	3.3 (anti)	2.6 (syn)
H_f-H_h	2.8	4.3 (anti)	2.8 (syn)	4.3 (syn)
H_f-H_i (weak)	4.2	Too far	4.1, 4.1 (opposite)	Too far
H_g-H_h	2.9	2.5 (syn)	2.5 (syn)	2.2 (syn)
H_g-H_i	2.8	4.5, 5.0 (anti)	4.8, 4.4 (syn)	4.7, 4.6 (syn)
H_h-H_i	3.7	2.3 (syn)	2.4, 3.4 (syn)	3.5, 2.7 (syn)

*The distances only include distances between protons in the more abundant conformer.

- There are several cross-peaks considered 'non-informative' because they are likely to be observed in all conformation, for example correlations: H_a-H_e, H_d-H_e, H_d-H_f, H_e-H_f, H_f-H_g, H_g-H_h and H_h-H_i. These nOe cross-peaks have been discarded for further analysis.
- There are several unexpected weak nOe cross-peaks (H_a-H_i, H_d-H_h, H_d-H_i, H_e-H_g, H_e-H_h and H_e-H_i); they are unexpected because they would have to be generated by hydrogens spatially far apart (if they were in the same molecule). It is likely that these nOe are produced by interactions between different molecules. If the linear conformation **A** arrangement observed in the crystal is considered, the shorter distances between these protons would be 2.3 Å (H_e-H_g), 5.7 Å (H_d-H_h), 4.1 Å (H_e-H_h) and 'too far' (H_d-H_i, H_e-H_i and H_a-H_i). Except for H_e-H_g, all these nOe cannot be observed in that arrangement, so, it can be concluded that the molecules are assembled in solution and that this is not like the conformer **A**.
- If the H_f-H_h cross-peak is generated by an intramolecular interaction, it favours the **B** conformation because it would be observed in the 'left side' but unlikely to be detected in any linear conformation. The high intensity of the H_f-H_h cross-peak may imply that the major conformation in solution is a bent one in a shape similar to the 'left side' of conformer **B** (Figure S5c).
- H_f-H_i cross-peak observed is weak which means that maybe it is of intermolecular nature, but if the bond configuration of 'left side' of the **B** conformer is considered, this interaction is likely to be observed in certain bent conformations. If an intermolecular interaction in the **A** conformation is considered, the shortest distance would be 5.3 Å, which is too long to be observed. As it is impossible to be detected as an intermolecular or intramolecular interaction in **A** conformation, it is concluded that the presence of H_f-H_i cross-peak discard **A** conformation as the major conformation in solution.

Liquid crystal formation as seen by solution-state NMR

Non-sonicated samples of $\mathbf{1}_{\mathbf{RR}}$ dissolved in 1,4-dioxane (deuterated or not) develop liquid-crystal droplets, typically after five days. During this time, the frequency of hydrogen atoms bonded to nitrogen shifts to lower frequencies, while hydrogen atoms attached to carbon shift to higher frequencies except for the methylene hydrogens (Figure S5). These shifts have been descried in self-assembled systems in solution when concentration is increased as resulting from hydrogen atoms bonded to carbon being affected by ring-currents, so that they would be unshielded; then, their signals shift to higher frequencies.[9] Concomitantly, a shielding of hydrogens bonded to nitrogen atoms would be observed.[10] The opposite phenomenon is observed in the 1,4-dioxane – $\mathbf{1}_{\mathbf{RR}}$ system; it seems that gelator molecules disappear from solution as they form liquid crystal droplets.

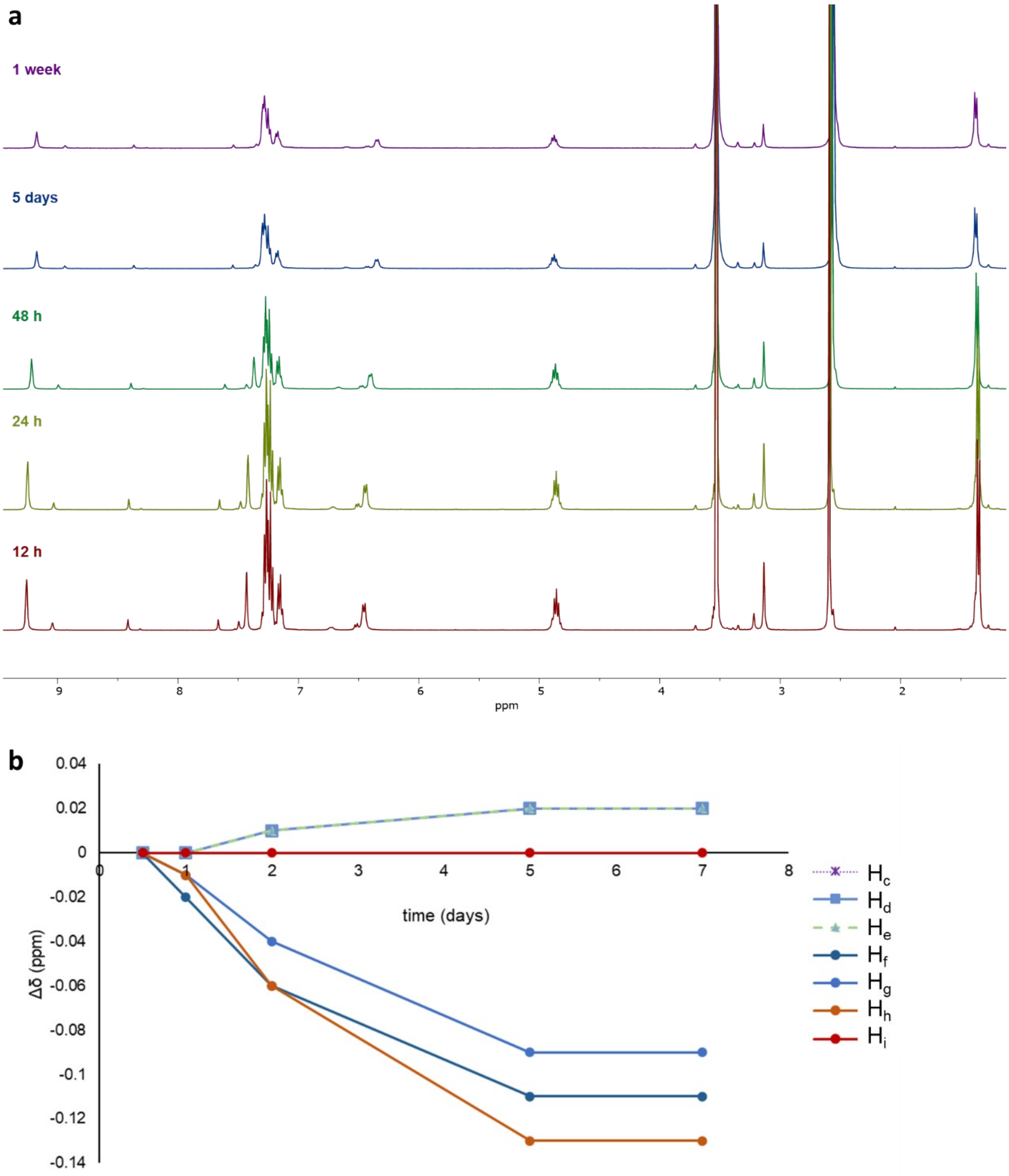

Figure S7. (a) 600 MHz ^{1}H of 1_{RR} in 1,4-dioxane-d_8 recorded at 25 ºC as a function of time. b, Chemical shift displacement ($\Delta\delta = \delta_{0.5days} - \delta_t$) plotted in function of time (days). Only the major conformer's peak changes are plotted.

Diffusion Ordered SpectroscopY (DOSY).

We used a double echo-stimulated, convection compensated pulse sequence, using bipolar pulsed gradients (the Varian' Dbppste_cc implementation) to estimate the diffusion coefficient of the molecules in solution. The non-uniformity of the field gradients was calibrated, and the results used to process the data.

Twenty pulse amplitudes ranging from 1.9 to 27.6 G cm^{-1} in equal steps of gradient squared were used. Sixteen transients, four steady state transients, and 32768 complex data points covering 9.6 kHz were used. The diffusion-encoding pulsed gradient duration was 2 ms. The diffusion time was 200 ms. The gradient stabilisation delay was 2 ms. The repetition time was 5.4 s, of which 3.4 s comprised the acquisition time. The results were analysed using mono-exponential fittings, although the first two points were discarded due to signal abnormalities. The unbalancing factor (alpha) was 0.15.

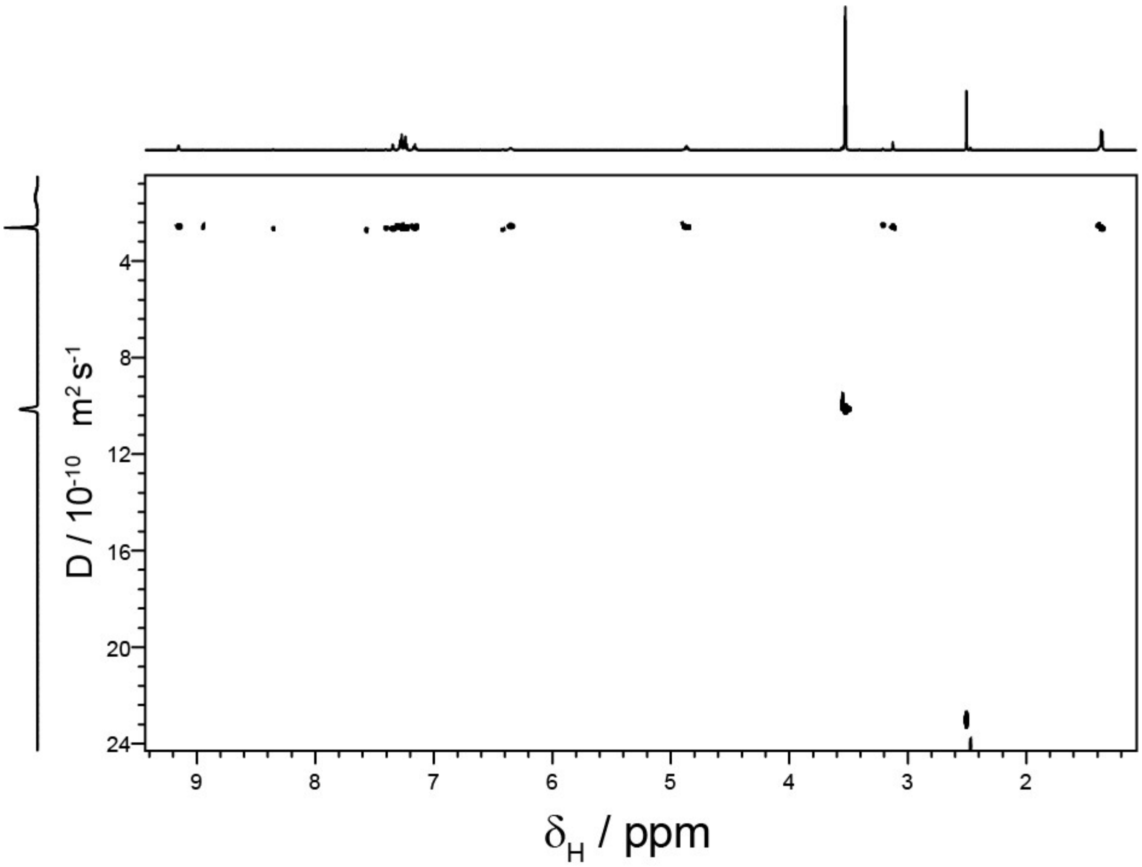

Figure S8. 600 MHz convection compensated ^{1}H-DOSY acquired at 25 ºC.

The analysis of the DOSY can be found on the main text.

<u>Supplementary Section 5. Additional figures</u>

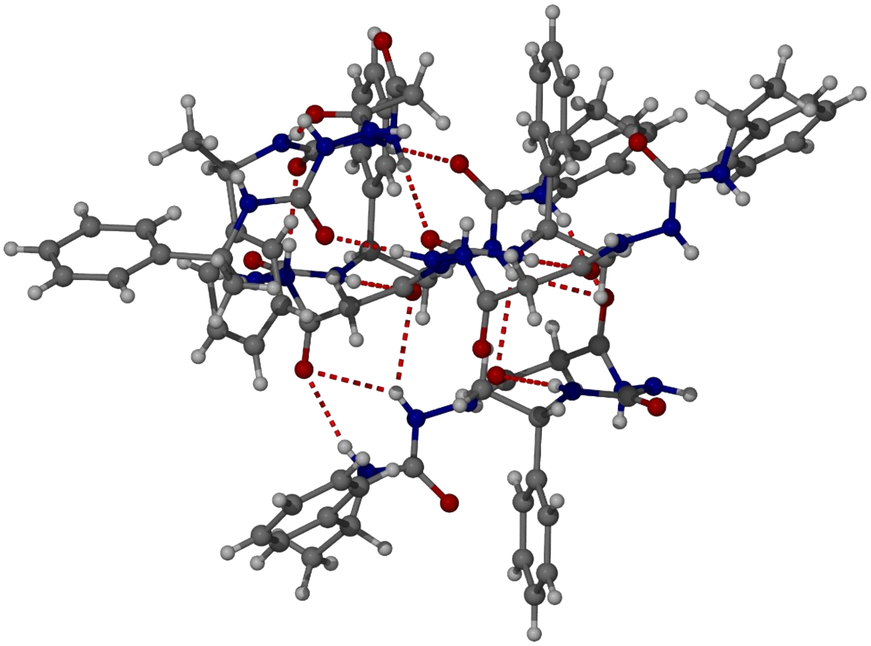

Figure S9. X-ray crystal structure of $\mathbf{1}_{\mathbf{RS}}$.

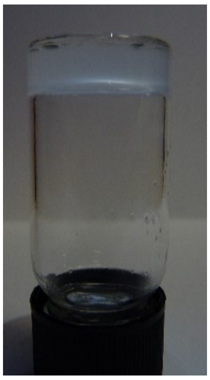

Figure S10. A 1 mL $\mathbf{1}_{\mathbf{RR}}$ hydrogel at 0.25 % w/v. Photograph taken 24 h after vial inversion.

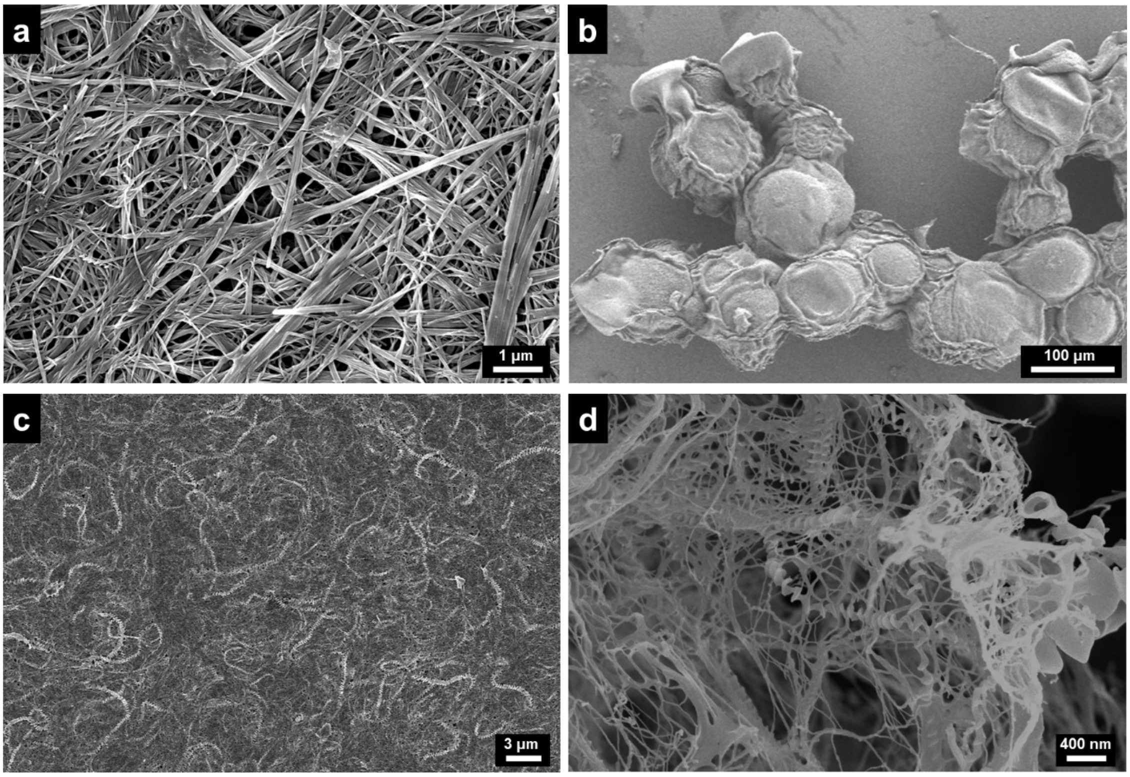

Figure S11. Complementary SEM pictures. (a) High magnification of **TF-gel**. (b) Liquid crystalline droplets. (c) Low magnification **HH-gel**. (d) Freeze dried **HH-gel**.

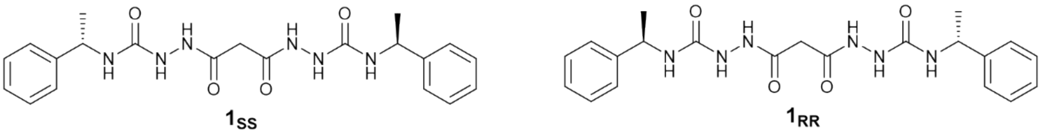

1_{SS} 1_{RR}

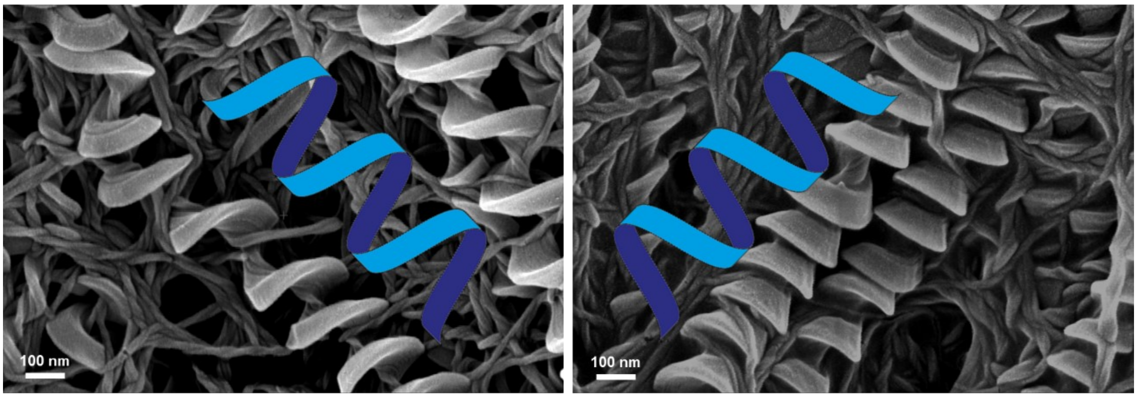

Figure S12. Handedness of hyper-helical fibres of HH-gel in function of gelator isomer.

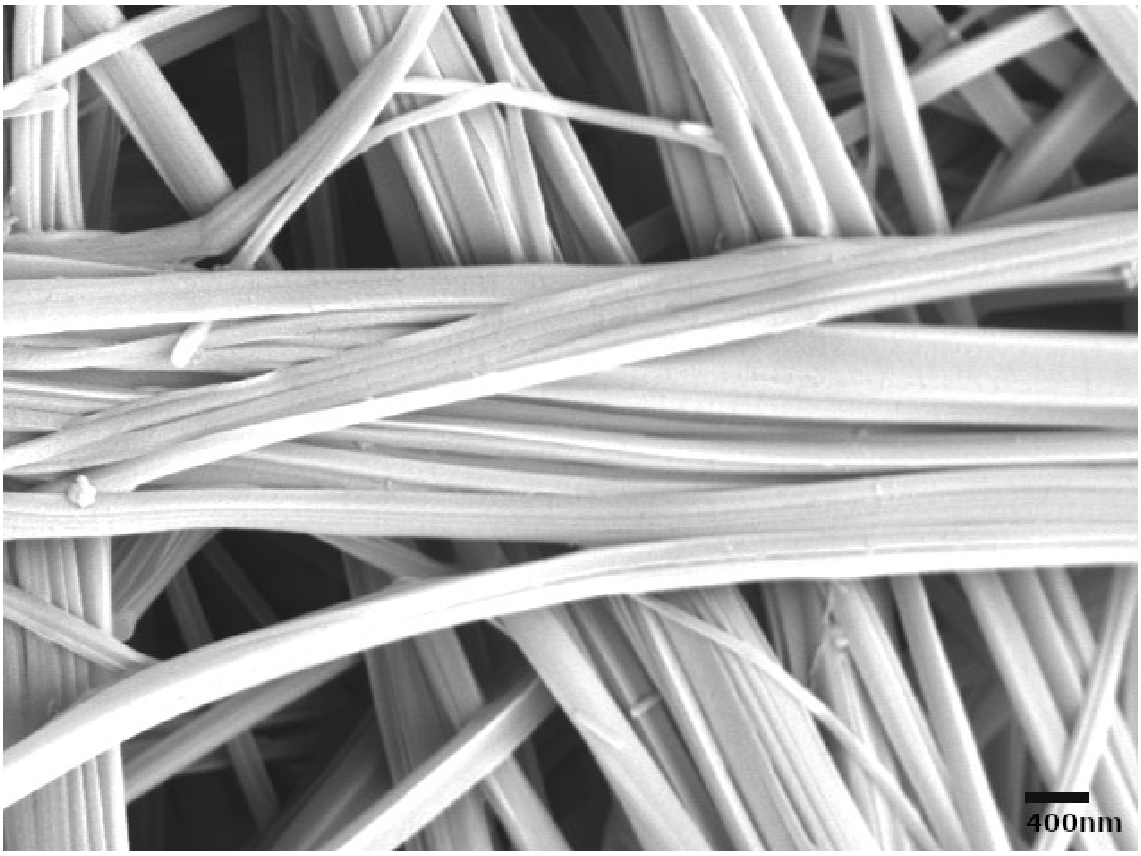

Figure S13. Zoomed SEM image of the TF-gel xerogel obtained from dioxane solution.

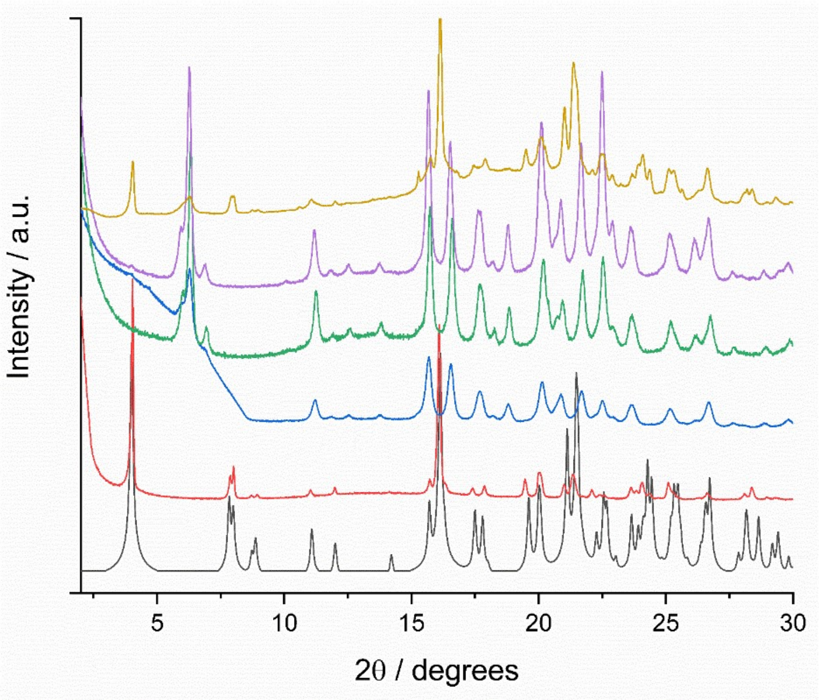

Figure S14. XRPD patterns of (top to bottom) air dried xerogels of $\mathbf{1}_{\mathbf{RR}}$ from nitrobenzene (yellow), acetonitrile (purple), propan-1-ol (green), chlorobenzene (blue) and 1,4-dioxane (red) and the calculated XRPD pattern from the $\mathbf{1}_{\mathbf{RR}}$ ethanol solvate (black). The 1,4-dioxane xerogel closely matches the ethanol solvate pattern, however this appears to be as a result of solid state conversion during the drying process. The acetonitrile, chlorobenzene and propan-1-ol xerogels match the xerogel phase obtained from freeze

drying while the nitrobenzene xerogel is a mixture of this phase and material corresponding to the isostructural ethanol and water solvate phases, apparently arising as a result of drying-induced conversion.

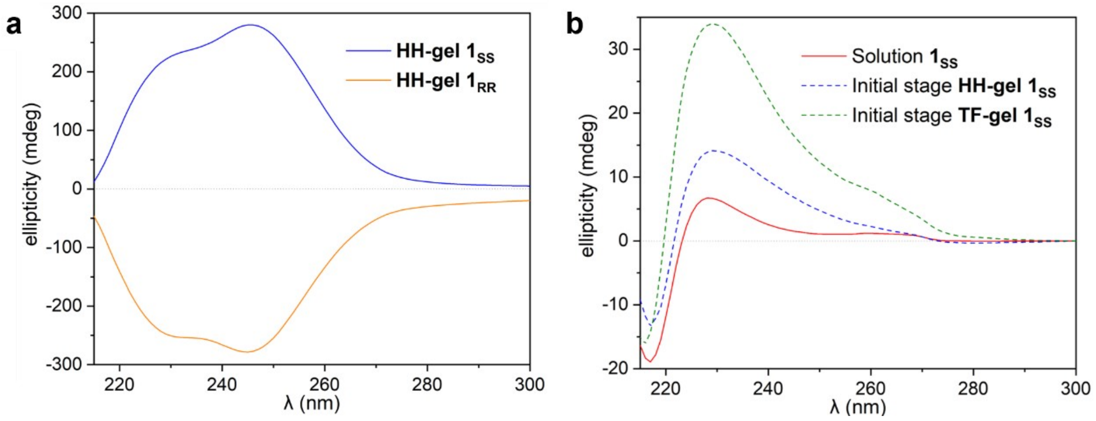

Figure S15. Additional CD spectra. (a) CD spectra **HH-gels** of $\mathbf{1_{SS}}$ and $\mathbf{1_{RR}}$. (b), CD of **HH-gel** and **TF-gel** at the initial stage of the gelation just after applying ultrasound in comparison with the $\mathbf{1_{SS}}$ 1,4-dioxane solution.

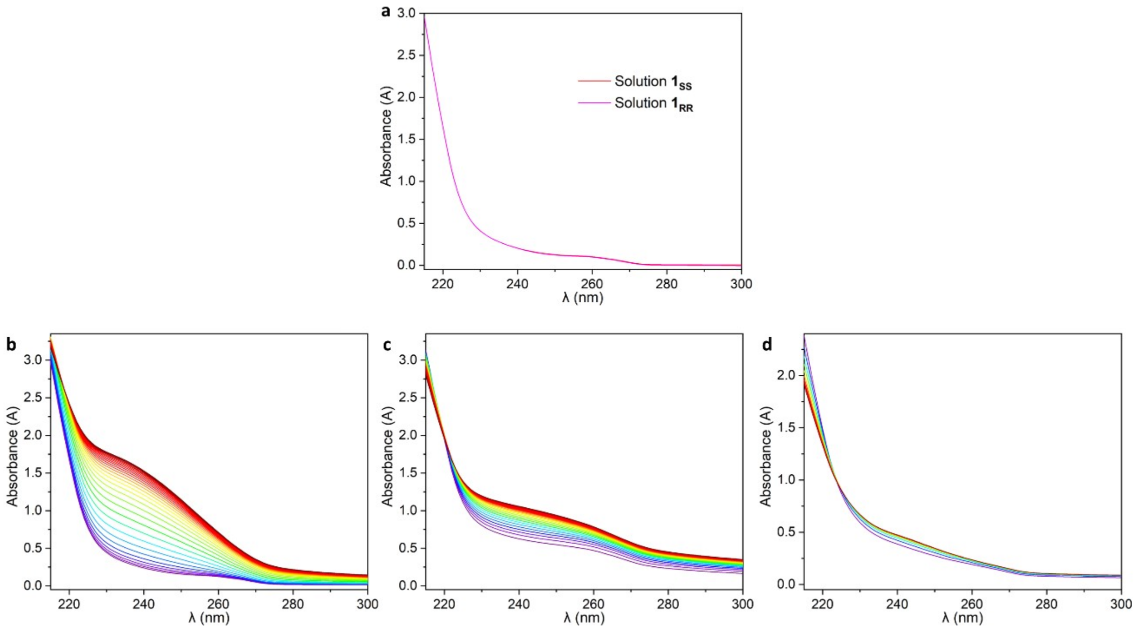

Figure S16. Absorbance spectra of (a) solutions $\mathbf{1_{SS}}$ and $\mathbf{1_{RR}}$. and gelation of (b) **HH-gel**, (c) **TF-gel** and (d) **LC-gel**.

a

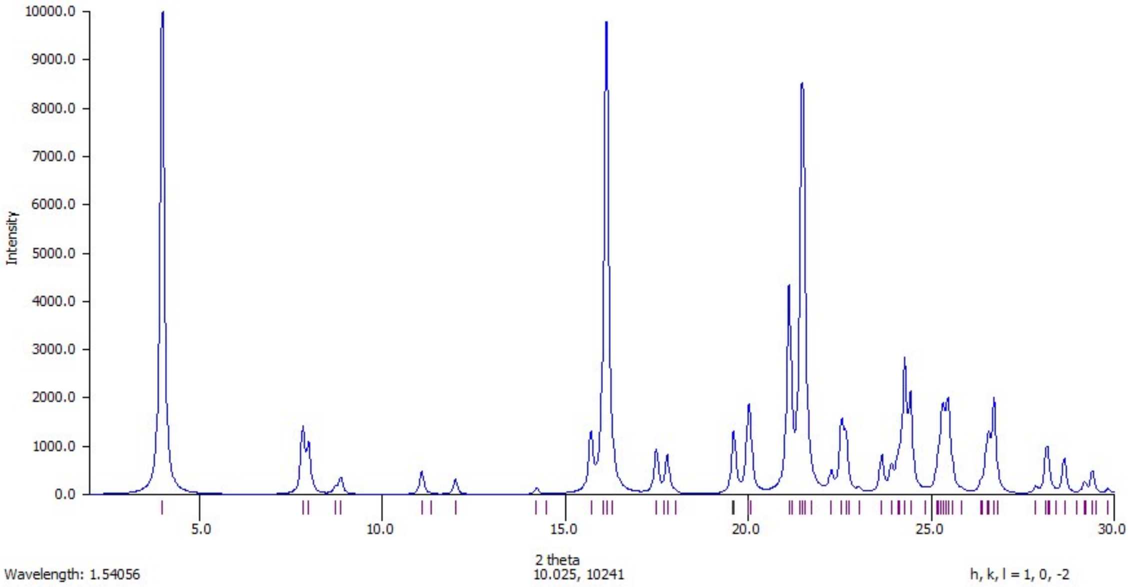

10000.0
9000.0
8000.0
7000.0
6000.0
5000.0
4000.0
3000.0
2000.0
1000.0
0.0
Intensity
5.0
10.0
15.0
20.0
25.0
30.0
2 theta
Wavelength: 1.54056
10.025, 10241
h, k, l = 1, 0, -2

b

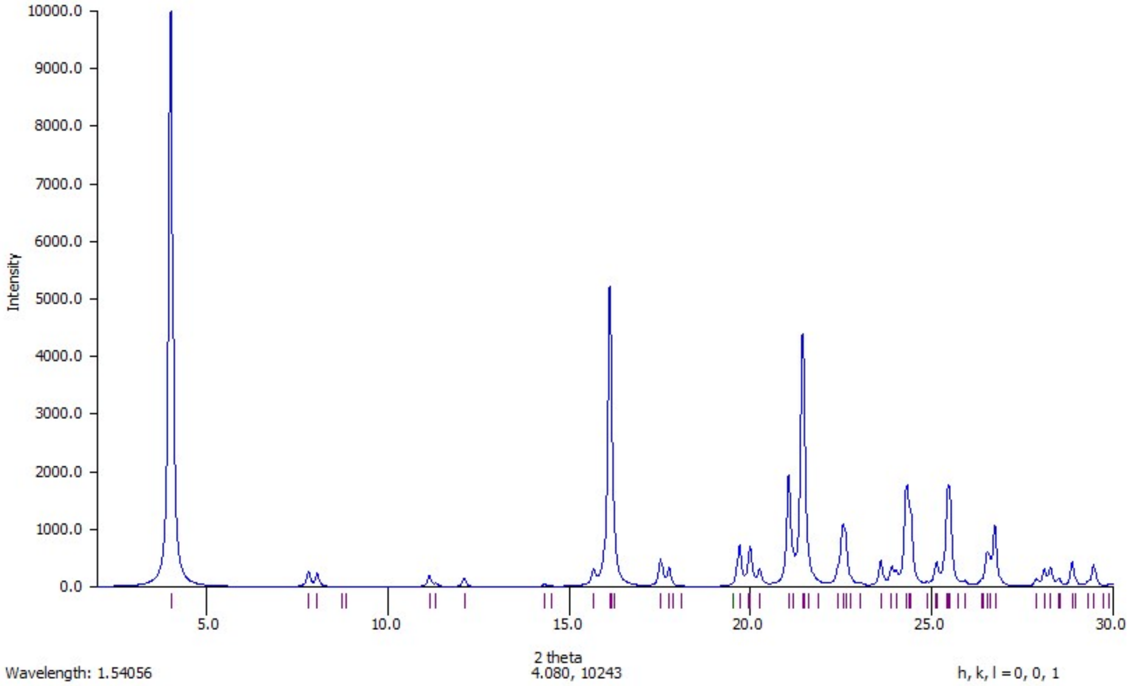

10000.0
9000.0
8000.0
7000.0
6000.0
5000.0
4000.0
3000.0
2000.0
1000.0
0.0
Intensity
5.0
10.0
15.0
20.0
25.0
30.0
2 theta
Wavelength: 1.54056
4.080, 10243
h, k, l = 0, 0, 1

c

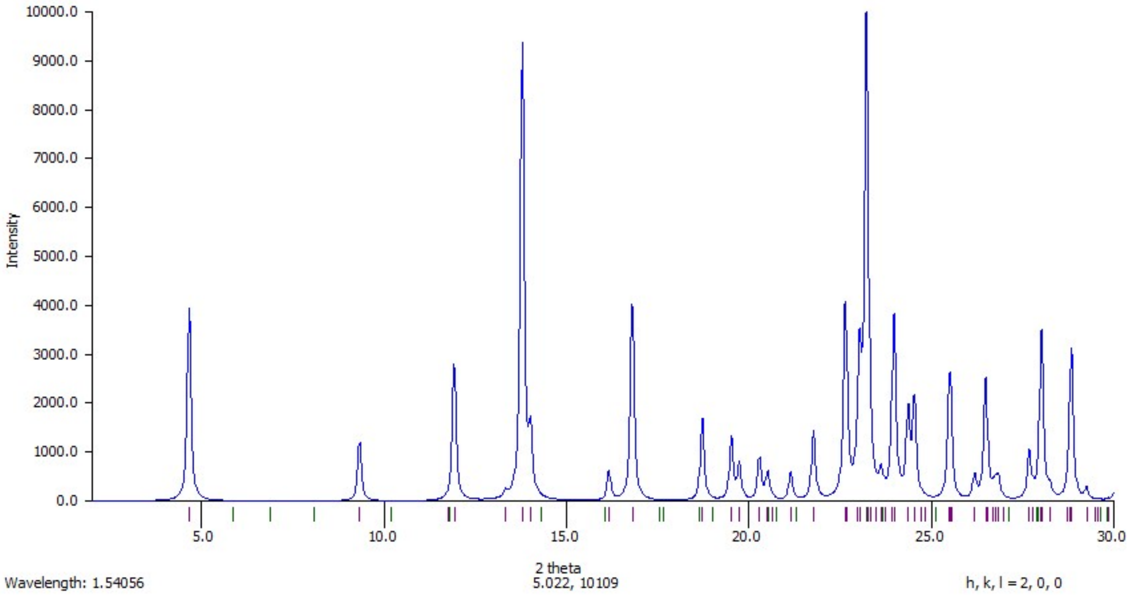

Intensity
2 theta
Wavelength: 1.54056
5.022, 10109
h, k, l = 2, 0, 0

d

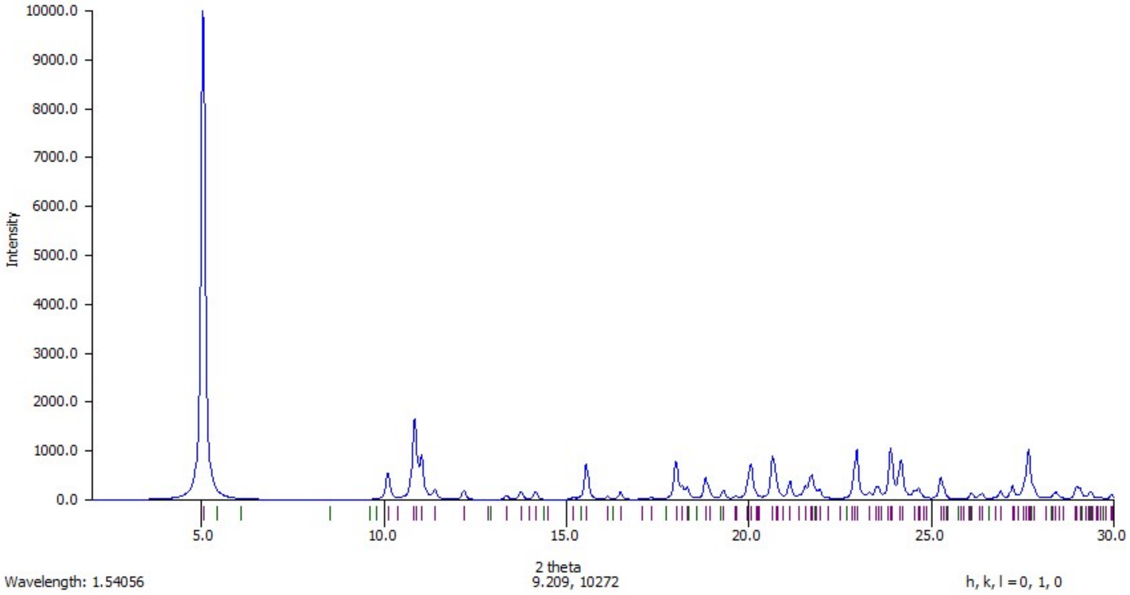

Intensity
2 theta
Wavelength: 1.54056
9.209, 10272
h, k, l = 0, 1, 0

e

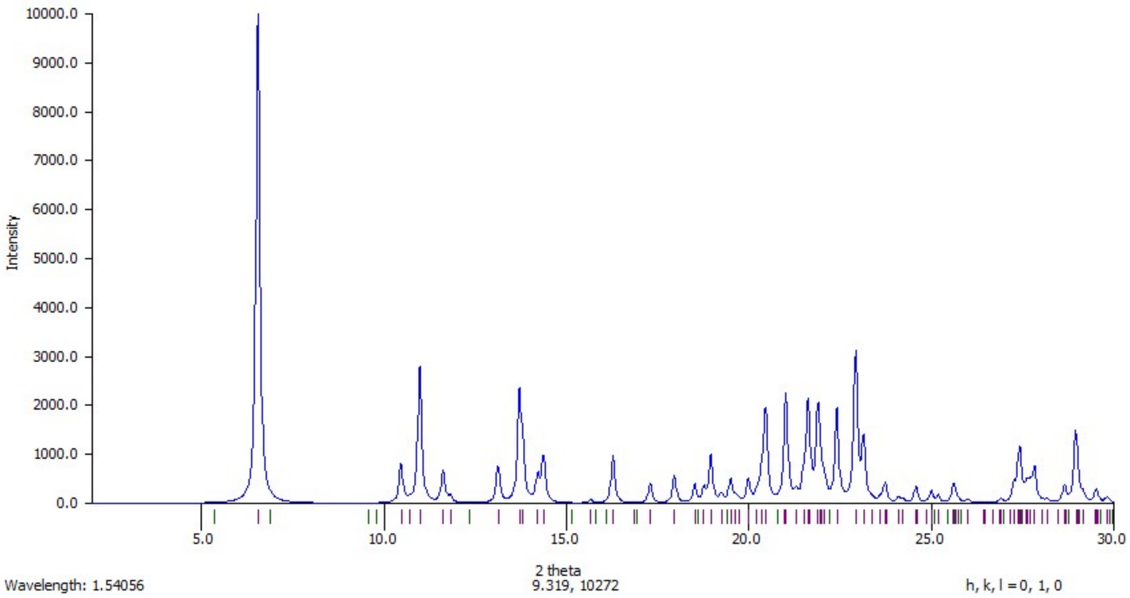

Figure S17. Calculated XRPD patterns using the CSD Mercury software.[11] (a) $\mathbf{1}_{\mathbf{RR}}$·EtOH; (b) $\mathbf{1}_{\mathbf{RR/SS}}$·2$H_2O$; (c) $\mathbf{1}_{\mathbf{RR/SS}}$·2MeCN (Form A); (d) $\mathbf{1}_{\mathbf{RR/SS}}$·2MeCN (Form B); (e) $\mathbf{1}_{\mathbf{RS}}$.

The sonication of the LC droplets was also tested at high temperature. A gel appeared almost instantly upon starting sonication to give highly heterogeneous gels. The resulting material was impossible to load into a syringe and transfer into the CD cuvette for further experiments. The morphology of the microstructure of the heterogeneous gel obtained was observed under SEM and same kind of fibrils similar to the LC-gel obtained sonicating at 25ºC, were observed, Figure S18.

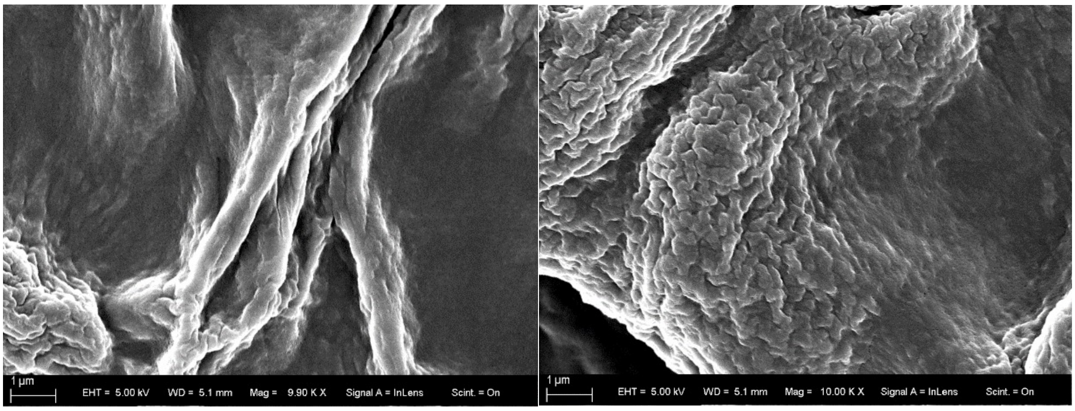

Figure S18. SEM images of the heterogeneous, difficult to handle gel from attempts to form LC-gel at elevated temperature.

Supplementary Section 6. SANS study

The SANS data was fitted to 1-level and 2-level Beaucage model. The generalized summed model used to fit the data is as follows:

$$I(Q) = Aq^{-power} + \sum_{i}^{N}\left[G_i \exp\left(-\frac{q^2 R_{g,i}^{\ 2}}{3}\right) + B_i \exp\left(-\frac{q^2 R_{g,(i+1)}^{\ 2}}{3}\right)\left(\frac{1}{q_i^*}\right)^{Pow_i}\right] + background$$

where

$$q_i^* = q\left[\operatorname{erf}\left(\frac{qR_{g,i}}{\sqrt{6}}\right)\right]^{-3}$$

- The first term is the power law model. The **TF**-gel analysis did not require this term.
- The second term is the unified exponential/R_g model for N structural levels.
    - For the **LC-** and **TF**-gels, $N = 2$.
    - For **HH**-gel up to 11 h 13 min, $N = 2$.
    - For **HH**-gel from 14 h 57 min to the end, $N = 1$.
- The third term is the incoherent background, accounting for noise.
- Parameters:
    - The scattering vector $q = (\frac{4\pi}{\lambda})\sin\frac{\theta}{2}$
        - Neutron wavelength λ
        - Scattering angle θ
    - A, G_i, and B_i are scaling factors.
    - R_g is the radius of gyration.
    - (-)Power and Pow_i are the exponential intensity decay

Table S5: Timeline of when each low-, mid- and high-q SANS measurement for each time point of each gel began after the gel samples were finished sonicating, in terms of h:min. A time point was considered complete after its high-q measurement was complete: i.e. **HH-gel** time point 1 was completed when the gel was 1 h 17 min old.

	HHGel			TFGel			LCGel		
**Time Point**	low q	middle q	high q	low q	middle q	high q	low q	middle q	high q
1	0:05	0:46	1:12	0:09	0:34	1:03	0:07	0:47	1:11
2	6:21	6:55	7:21	3:57	4:31	4:57	3:07	3:48	4:12
3	10:08	10:42	11:08	7:44	8:18	8:44	7:09	7:50	8:14
4	13:45	14:18	14:52	11:27	12:08	12:34	10:05	10:46	11:10
5	17:34	18:28	18:35	15:10	15:45	16:11	11:33	12:14	12:38
6	21:12	21:46	22:12	18:48	19:22	19:48	12:59	13:40	13:53
7	24:48	25:29	25:55						

Table S6: Fitting parameters of Power Law + 2 Level Beaucage model fitted to each time point for each gel. The gel age is the time after sonication at which the data was collected. The **HH-gel** only required a 1 Level Beaucage model for the last 4 time points, so the second level's set of parameters were not used. Likewise, the **TF-gel** did not require a Power Law term to be fitted, so those parameters were not used. The numbers in parentheses represent the uncertainty in the last significant figure of each value.

Gel	Age	Coefficient, A	(-) Power	G_1 (cm^{-1} sr^{-1})	$R_{g,1}$ (Å)	B_1 (cm^{-1} sr^{-1} A^{-Pow1})	Pow_1	G_2 (cm^{-1} sr^{-1})	$R_{g,2}$ (Å)	B_2 (cm^{-1} sr^{-1} A^{-Pow2})	Pow_2
**HH-gel**	1 hr 17 min	1.0(3)E-09	3.96(6)	1	450(30)	3(1)E-06	2.41(8)	0.0242(6)	21.4(3)	6.0(9)E-05	3.0(1)
	7 hr 26 min	2.9(7)E-09	3.84(4)	1.44(9)	190(7)	9(2)E-05	1.95(5)	0.023(1)	21.8(7)	7(1)E-05	2.7(1)
	11 hr 13 min	8(2)E-09	3.64(4)	1.26(6)	166(5)	9(1)E-05	2.00(4)	0.019(2)	20(1)	3(1)E-05	3.0(3)
	14 hr 57 min	1.9(4)E-08	3.50(3)	1.22(5)	146(3)	6.8(2)E-05	2.15(1)	--	--	--	--
	18 hr 40 min	1.4(2)E-07	3.15(3)	0.87(4)	125(3)	3.7(2)E-05	2.32(2)	--	--	--	--
	22 hr 17 min	2.8(5)E-07	3.02(3)	0.78(3)	119(3)	2.4(2)E-05	2.45(2)	--	--	--	--
	26 hr 0 min	2.7(5)E-07	3.01(3)	0.79(4)	124(3)	2.1(1)E-05	2.46(2)	--	--	--	--
**TF-gel**	1 hr 08 min	--	--	560(10)	860(10)	1.76(4)E-06	3.146(5)	0.0129(5)	26.2(4)	1.6(2)E-04	2.20(6)
	5 hr 02 min	--	--	2290(70)	1270(20)	2.25(2)E-05	2.720(2)	0.0104(5)	21.0(2)	3.3(6)E-05	3.3(1)
	8 hr 49 min	--	--	1980(40)	1150(10)	5.35(5)E-05	2.579(2)	0.0012(5)	19.8(2)	1.0(3)E-05	4.0(2)
	12 hr 39 min	--	--	2210(40)	1170(10)	7.09(6)E-05	2.542(2)	0.0010(9)	20.9(3)	5(2)E-06	4.0(3)
	16 hr 16 min	--	--	2420(40)	1180(10)	7.62(7)E-05	2.546(2)	0.001(1)	20.9(3)	4(2)E-06	4.0(3)
	19 hr 53 min	--	--	2460(50)	1190(10)	7.27(7)E-05	2.557(2)	0.001(1)	21.4(4)	3(2)E-06	4.0(4)
**LC-gel**	1 hr 16 min	2.8(8)E-05	2.24(5)	1.4(1)	128(4)	3(1)E-04	1.89(9)	0.10(1)	21.3(6)	7(1)E-05	2.5(1)
	4 hr 17 min	8(1)E-09	3.74(3)	6.9(2)	205(2)	0.0011(1)	1.71(2)	0.142(9)	22.4(3)	9(1)E-05	2.57(8)
	8 hr 19 min	5.7(6)E-09	3.88(2)	7.0(1)	198(2)	0.0013(1)	1.71(2)	0.143(8)	22.5(3)	2.2(2)E-4	2.04(7)
	11 hr 15 min	6.8(7)E-09	3.85(2)	6.7(1)	188(2)	0.0012(1)	1.74(2)	0.157(9)	22.9(2)	2.0(2)E-04	2.04(7)
	12 hr 43 min	6.4(7)E-09	3.86(2)	6.7(1)	189(2)	0.0013(1)	1.73(2)	0.156(9)	23.0(3)	1.5(2)E-04	2.15(8)
	13 hr 58 min	1.1(1)E-08	3.76(2)	6.6(1)	184(2)	0.0013(2)	1.72(3)	0.15(1)	22.7(4)	1.5(3)E-04	2.1(1)

HH-Gel 1 hr 17 min: Power Law + 2-Level Beaucage

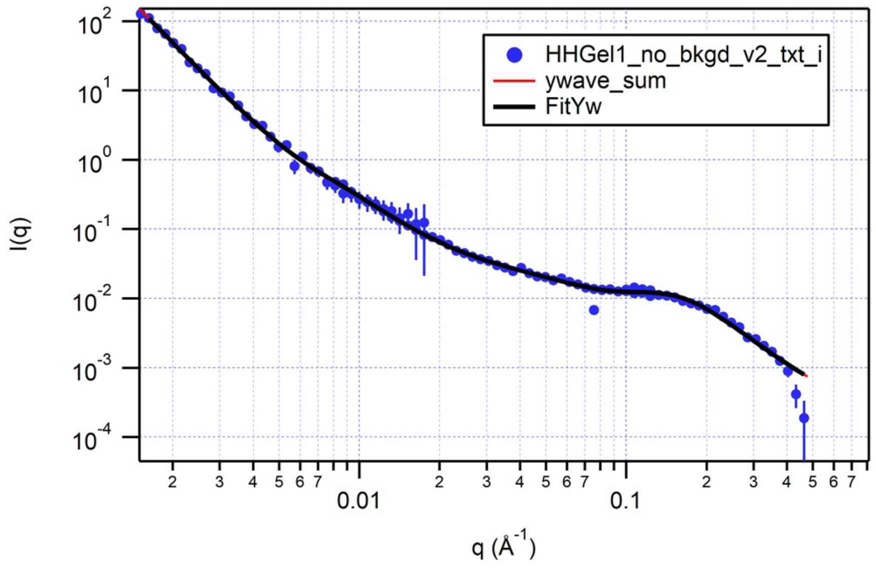

Coefficient, A	9.61E-10	±	3.49E-10
(-)Power	3.96432	±	0.05872
Incoherent Bgd (cm^{-1})	0	±	0
Scale	1	±	0
G_1 (cm^{-1} sr^{-1})	1	±	0
$R_{g,1}$ (Å)	445.691	±	34.1805
B_1 (cm^{-1} sr^{-1} A^{-Pow})	3.15E-06	±	1.10E-06
Pow_1	2.40843	±	0.083954
G_2 (cm^{-1} sr^{-1})	0.024167	±	0.000576
$R_{g,2}$ (Å)	21.3621	±	0.34268
B_2 (cm^{-1} sr^{-1} A^{-Pow})	5.99E-05	±	8.66E-06
Pow_2	3.01938	±	0.101653
bkg (cm^{-1} sr^{-1})	0.000188	±	0
fit range	0.0015<q<0.5		
χ^2	1.37187		

HH-Gel 7 hr 26 min: Power Law + 2-Level Beaucage

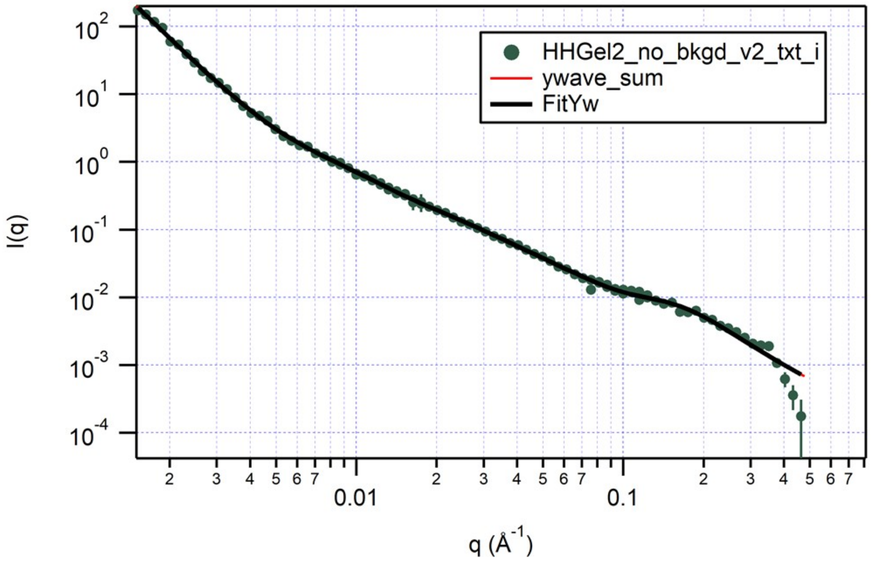

Coefficient, A	2.90E-09	±	6.58E-10
(-)Power	3.83634	±	0.036214
Incoherent Bgd (cm^{-1})	0	±	0
Scale	1	±	0
G_1 (cm^{-1} sr^{-1})	1.4397	±	0.095483
$R_{g,1}$ (Å)	190.048	±	6.75896
B_1 (cm^{-1} sr^{-1} A^{-Pow})	9.19E-05	±	1.69E-05
Pow_1	1.95198	±	0.047603
G_2 (cm^{-1} sr^{-1})	0.023205	±	0.001218
$R_{g,2}$ (Å)	21.8309	±	0.701869
B_2 (cm^{-1} sr^{-1} A^{-Pow})	6.87E-05	±	1.24E-05
Pow_2	2.71836	±	0.130349
bkg (cm^{-1} sr^{-1})	0.000176	±	0
fit range	0.0015<q<0.5		
χ^2	1.66434		

HH-Gel 11 hr 13 min: Power Law + 2-Level Beaucage

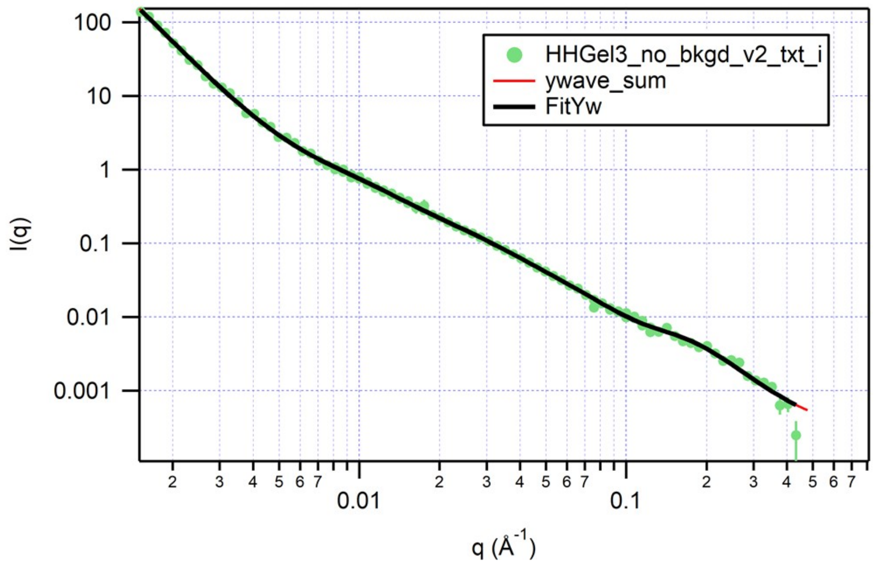

Coefficient, A	7.83E-09	±	1.74E-09
(-)Power	3.64362	±	0.035525
Incoherent Bgd (cm^{-1})	0	±	0
Scale	1	±	0
G_1 (cm^{-1} sr^{-1})	1.26139	±	0.064858
$R_{g,1}$ (Å)	165.637	±	4.9144
B_1 (cm^{-1} sr^{-1} A^{-Pow})	9.21E-05	±	1.49E-05
Pow_1	2.00124	±	0.043767
G_2 (cm^{-1} sr^{-1})	0.019122	±	0.002023
$R_{g,2}$ (Å)	20.238	±	1.09999
B_2 (cm^{-1} sr^{-1} A^{-Pow})	3.08E-05	±	1.05E-05
Pow_2	3.02396	±	0.256252
bkg (cm^{-1} sr^{-1})	0.000248	±	0
fit range	0.0015<q<0.5		
χ^2	1.21153		

<u>**HH-Gel 14 hr 57 min: Power Law + 1-Level Beaucage**</u>

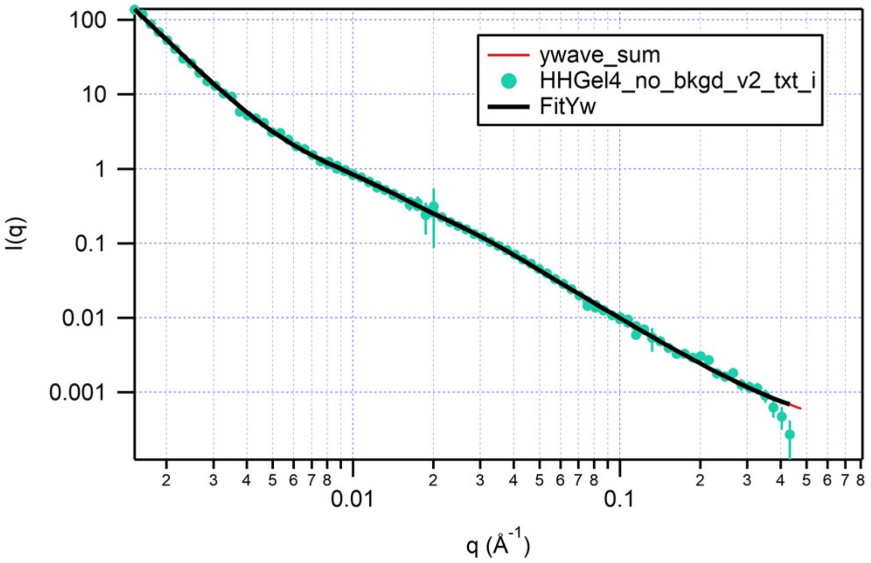

Coefficient, A	1.89E-08	±	3.82E-09
(-)Power	3.50102	±	0.032566
Incoherent Bgd (cm^{-1})	0	±	0
Scale	1	±	0
G_1 (cm^{-1} sr^{-1})	1.2246	±	0.047638
$R_{g,1}$ (Å)	146.392	±	3.45383
B_1 (cm^{-1} sr^{-1} A^{-Pow})	6.78E-05	±	2.53E-06
Pow_1	2.14877	±	0.012091
bkg (cm^{-1} sr^{-1})	0.00027	±	0
fit range	0.0015<q<0.5		
χ^2	1.28976		

HH-Gel 18 hr 40 min: Power Law + 1-Level Beaucage

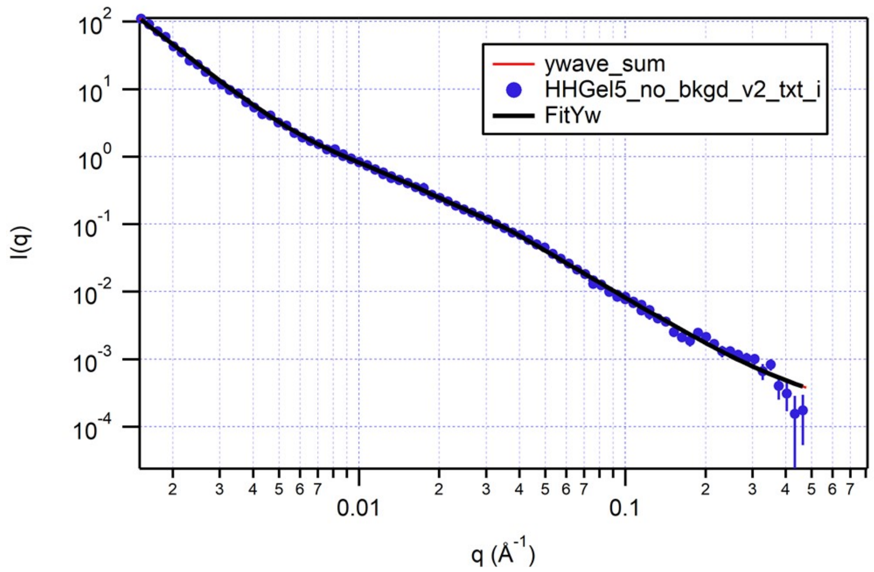

Coefficient, A	1.43E-07	±	2.40E-08
(-)Power	3.14805	±	0.027355
Incoherent Bgd (cm^{-1})	0	±	0
Scale	1	±	0
G_1 (cm^{-1} sr^{-1})	0.870179	±	0.036465
$R_{g,1}$ (Å)	125.235	±	3.09701
B_1 (cm^{-1} sr^{-1} A^{-Pow})	3.67E-05	±	1.93E-06
Pow_1	2.32531	±	0.017487
bkg (cm^{-1} sr^{-1})	0.000175	±	0
fit range	0.0015<q<0.5		
χ^2	1.40455		

HH-Gel 22 hr 17 min: Power Law + 1-Level Beaucage

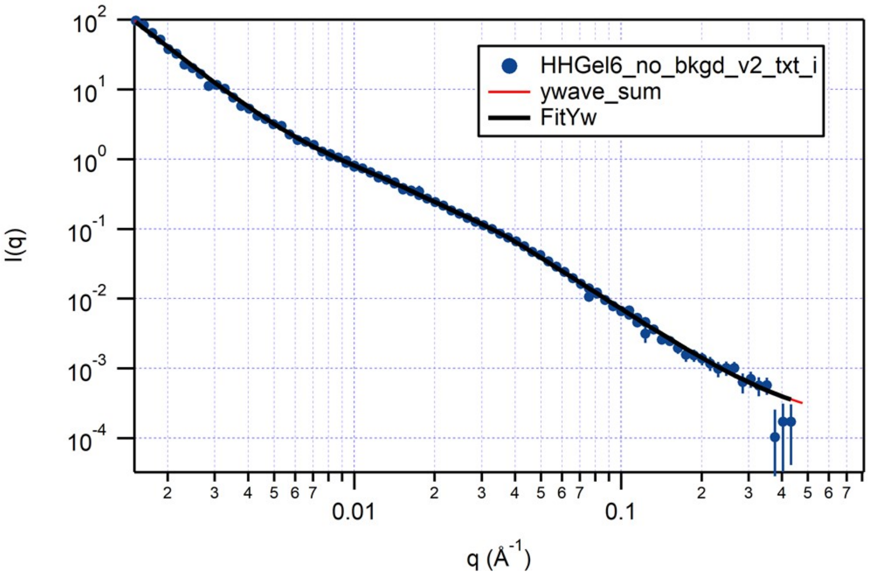

Coefficient, A	2.78E-07	±	4.58E-08
(-)Power	3.02329	±	0.026958
Incoherent Bgd (cm^{-1})	0	±	0
Scale	1	±	0
G_1 (cm^{-1} sr^{-1})	0.776008	±	0.03407
$R_{g,1}$ (Å)	119.202	±	3.03745
B_1 (cm^{-1} sr^{-1} A^{-Pow})	2.36E-05	±	1.58E-06
Pow_1	2.45088	±	0.022242
bkg (cm^{-1} sr^{-1})	0.000171	±	0
fit range	0.0015<q<0.5		
χ^2	1.44066		

HH-Gel 26 hr 0 min: Power Law + 1-Level Beaucage

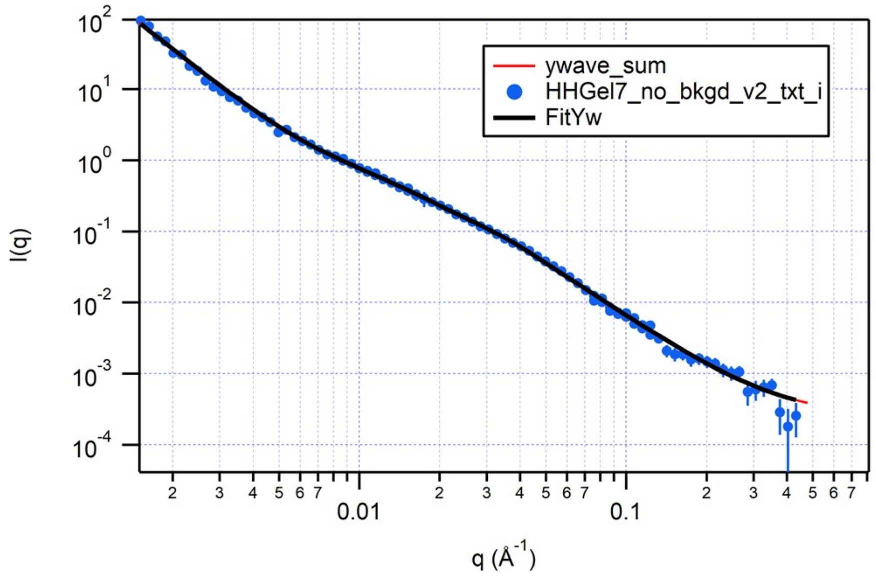

Coefficient, A	2.75E-07	±	5.17E-08
(-)Power	3.00707	±	0.030831
Incoherent Bgd (cm^{-1})	0	±	0
Scale	1	±	0
G_1 (cm^{-1} sr^{-1})	0.790035	±	0.037319
$R_{g,1}$ (Å)	123.62	±	3.2484
B_1 (cm^{-1} sr^{-1} A^{-Pow})	2.11E-05	±	1.45E-06
Pow_1	2.45894	±	0.022478
bkg (cm^{-1} sr^{-1})	0.000257	±	0
fit range	0.0015<q<0.5		
χ^2	1.64137		

TF-Gel 1 hr 08 min: 2-Level Beaucage

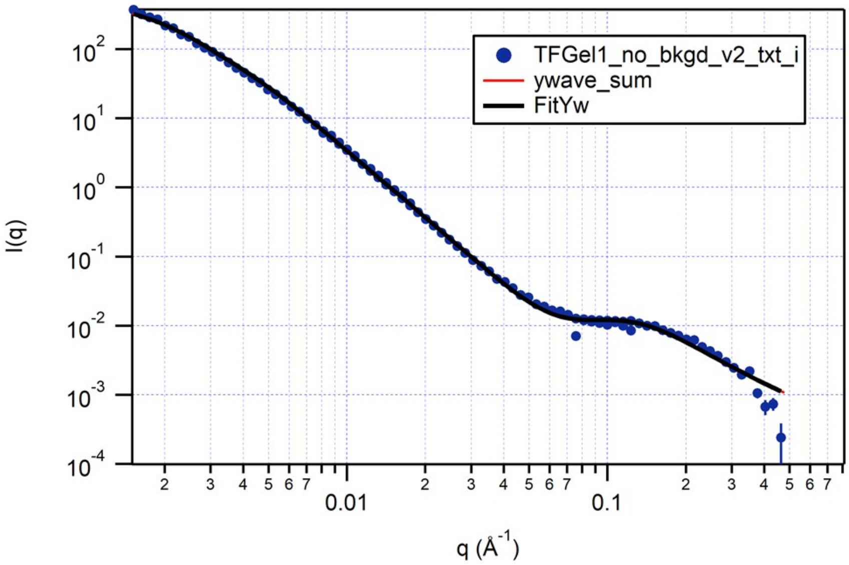

Scale	1	±	0
G_1 (cm^{-1} sr^{-1})	562.342	±	10.8486
$R_{g,1}$ (Å)	860.193	±	10.5681
B_1 (cm^{-1} sr^{-1} A^{-Pow})	1.76E-06	±	4.39E-08
Pow_1	3.14654	±	0.00541
G_2 (cm^{-1} sr^{-1})	0.012918	±	0.000535
$R_{g,2}$ (Å)	26.2437	±	0.383052
B_2 (cm^{-1} sr^{-1} A^{-Pow})	0.000164	±	1.57E-05
Pow_2	2.19849	±	0.062057
bkg (cm^{-1} sr^{-1})	0.00024	±	0
fit range	0.0015<q<0.5		
χ^2	3.84369		

<u>**TF-Gel 5 hr 02 min: 2-Level Beaucage**</u>

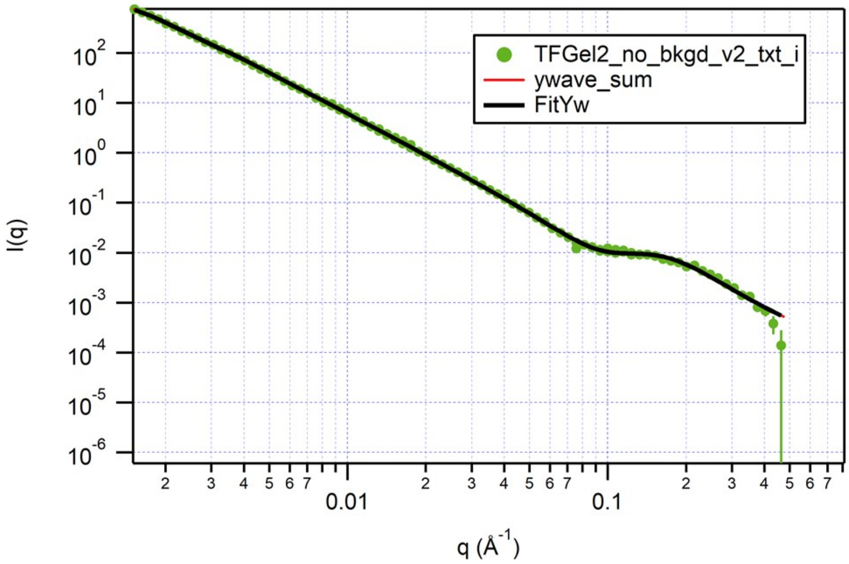

Scale	1	±	0
G_1 (cm^{-1} sr^{-1})	2289.08	±	71.8534
$R_{g,1}$ (Å)	1274.71	±	18.7898
B_1 (cm^{-1} sr^{-1} A^{-Pow})	2.25E-05	±	2.48E-07
Pow_1	2.71983	±	0.002398
G_2 (cm^{-1} sr^{-1})	0.010443	±	0.00052
$R_{g,2}$ (Å)	20.9685	±	0.235414
B_2 (cm^{-1} sr^{-1} A^{-Pow})	3.29E-05	±	5.68E-06
Pow_2	3.29148	±	0.116857
bkg (cm^{-1} sr^{-1})	0.00014	±	0
fit range	0.0015<q<0.5		
χ^2	3.45141		

TF-Gel 8 hr 49 min: 2-Level Beaucage

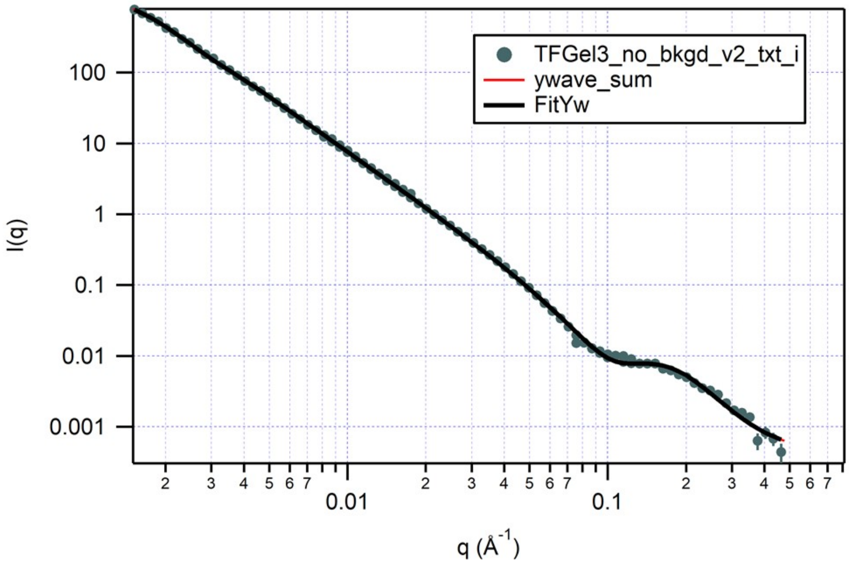

Scale	1	±	0
G_1 (cm^{-1} sr^{-1})	1985.43	±	36.5937
$R_{g,1}$ (Å)	1150.51	±	9.89293
B_1 (cm^{-1} sr^{-1} A^{-Pow})	5.35E-05	±	4.76E-07
Pow_1	2.57886	±	0.00194
G_2 (cm^{-1} sr^{-1})	0.001253	±	0.000543
$R_{g,2}$ (Å)	19.8218	±	0.196174
B_2 (cm^{-1} sr^{-1} A^{-Pow})	1.02E-05	±	2.59E-06
Pow_2	4	±	0.172371
bkg (cm^{-1} sr^{-1})	0.000442	±	0
fit range	0.0015<q<0.5		
χ^2	3.93199		

TF-Gel 12 hr 39 min: 2-Level Beaucage

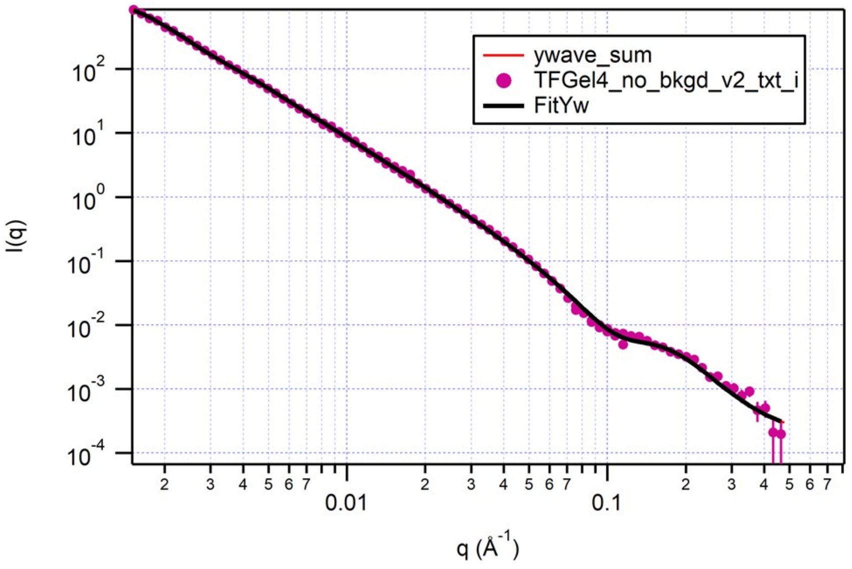

Scale	1	±	0
G_1 (cm^{-1} sr^{-1})	2210.4	±	40.5292
$R_{g,1}$ (Å)	1170.93	±	9.89753
B_1 (cm^{-1} sr^{-1} A^{-Pow})	7.09E-05	±	6.38E-07
Pow_1	2.54216	±	0.001937
G_2 (cm^{-1} sr^{-1})	0.001	±	0.000945
$R_{g,2}$ (Å)	20.8613	±	0.278373
B_2 (cm^{-1} sr^{-1} A^{-Pow})	5.35E-06	±	2.16E-06
Pow_2	4	±	0.261929
bkg (cm^{-1} sr^{-1})	0.000197	±	0
fit range	0.0015<q<0.5		
χ^2	4.63788		

TF-Gel 16 hr 16 min: 2-Level Beaucage

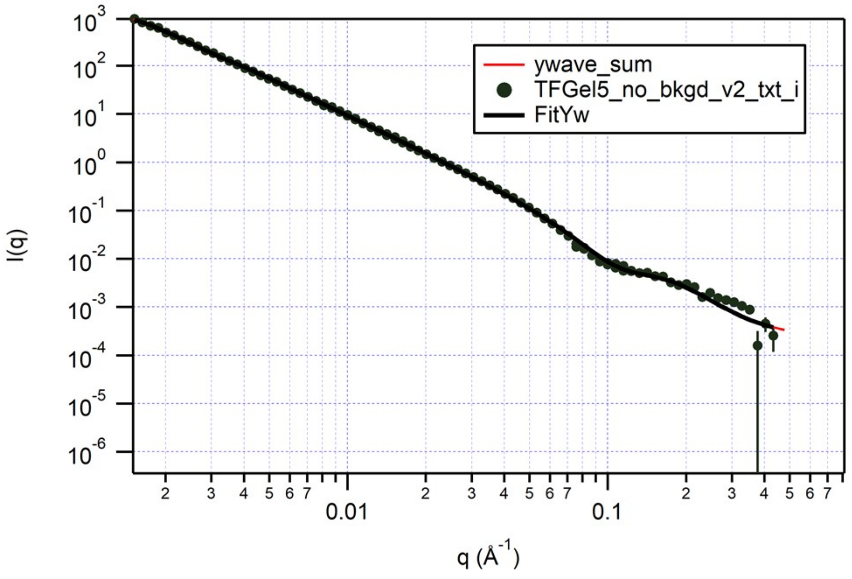

Scale	1	±	0
G_1 (cm^{-1} sr^{-1})	2419.6	±	44.8687
$R_{g,1}$ (Å)	1180.4	±	10.1709
B_1 (cm^{-1} sr^{-1} A^{-Pow})	7.62E-05	±	6.80E-07
Pow_1	2.54585	±	0.00191
G_2 (cm^{-1} sr^{-1})	0.001	±	0.001161
$R_{g,2}$ (Å)	20.9097	±	0.30931
B_2 (cm^{-1} sr^{-1} A^{-Pow})	4.42E-06	±	2.15E-06
Pow_2	4	±	0.313607
bkg (cm^{-1} sr^{-1})	0.000256	±	0
fit range	0.0015<q<0.5		
χ^2	4.87738		

TF-Gel 19 hr 53 min: 2-Level Beaucage

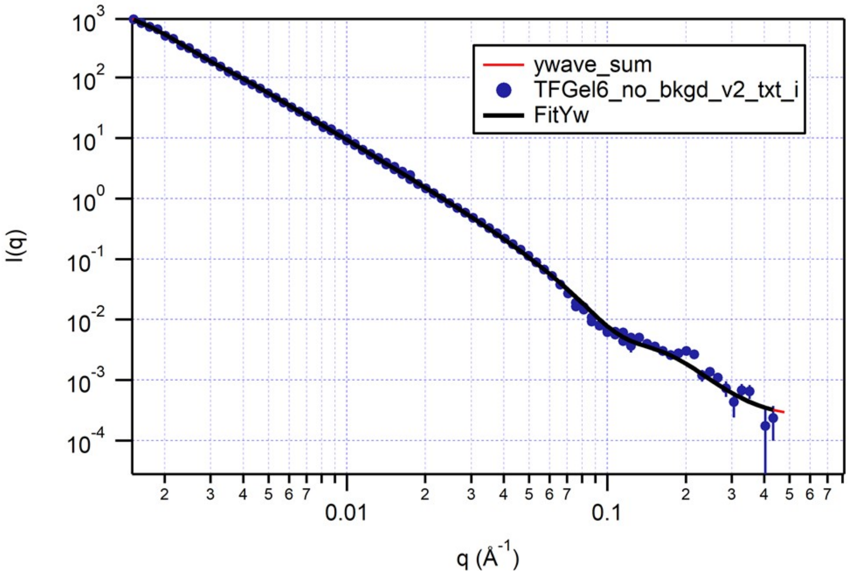

Scale	1	±	0
G_1 (cm^{-1} sr^{-1})	2461.83	±	46.6623
$R_{g,1}$ (Å)	1189.09	±	10.5537
B_1 (cm^{-1} sr^{-1} A^{-Pow})	7.27E-05	±	6.77E-07
Pow_1	2.55699	±	0.001975
G_2 (cm^{-1} sr^{-1})	0.001	±	0.001416
$R_{g,2}$ (Å)	21.4031	±	0.361715
B_2 (cm^{-1} sr^{-1} A^{-Pow})	3.06E-06	±	1.96E-06
Pow_2	4	±	0.405682
bkg (cm^{-1} sr^{-1})	0.000236	±	0
fit range	0.0015<q<0.5		
χ^2	5.33549		

<u>**LC-Gel 1 hr 16 min: Power Law + 2-Level Beaucage**</u>

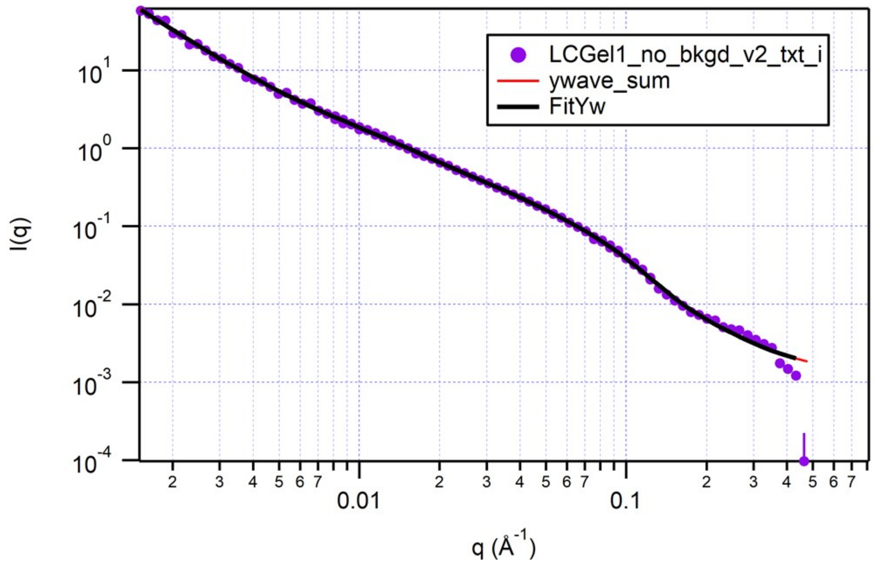

Coefficient, A	2.85E-05	±	8.21E-06
(-)Power	2.24	±	0.047587
Incoherent Bgd (cm^{-1})	0	±	0
Scale	1	±	0
G_1 (cm^{-1} sr^{-1})	1.42772	±	0.132387
$R_{g,1}$ (Å)	127.573	±	4.16393
B_1 (cm^{-1} sr^{-1} A^{-Pow})	0.000333	±	0.000123
Pow_1	1.88831	±	0.095455
G_2 (cm^{-1} sr^{-1})	0.104347	±	0.011539
$R_{g,2}$ (Å)	21.3399	±	0.566427
B_2 (cm^{-1} sr^{-1} A^{-Pow})	7.41E-05	±	1.40E-05
Pow_2	2.51988	±	0.114892
bkg (cm^{-1} sr^{-1})	0.001215	±	0
fit range	0.0015<q<0.5		
χ^2	1.91228		

LC-Gel 4 hr 17 min: Power Law + 2-Level Beaucage

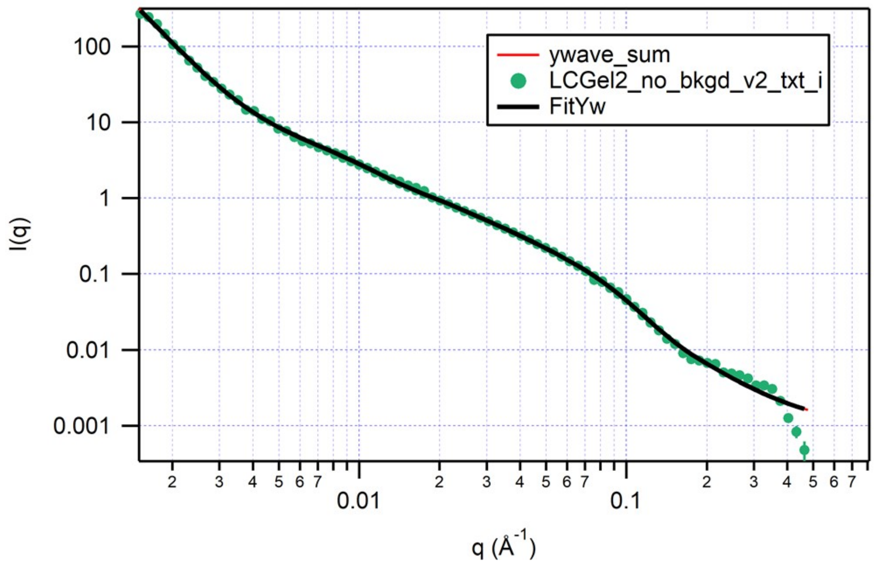

Coefficient, A	8.21E-09	±	1.41E-09
(-)Power	3.74431	±	0.027294
Incoherent Bgd (cm^{-1})	0	±	0
Scale	1	±	0
G_1 (cm^{-1} sr^{-1})	6.89984	±	0.155251
$R_{g,1}$ (Å)	204.842	±	2.39729
B_1 (cm^{-1} sr^{-1} A^{-Pow})	0.001103	±	1.02E-04
Pow_1	1.71012	±	0.021908
G_2 (cm^{-1} sr^{-1})	0.142192	±	0.008999
$R_{g,2}$ (Å)	22.4498	±	0.32521
B_2 (cm^{-1} sr^{-1} A^{-Pow})	9.28E-05	±	1.14E-05
Pow_2	2.56625	±	0.077921
bkg (cm^{-1} sr^{-1})	0.001	±	0
fit range	0.0015<q<0.5		
χ^2	2.66447		

LC-Gel 8 hr 19 min: Power Law + 2-Level Beaucage

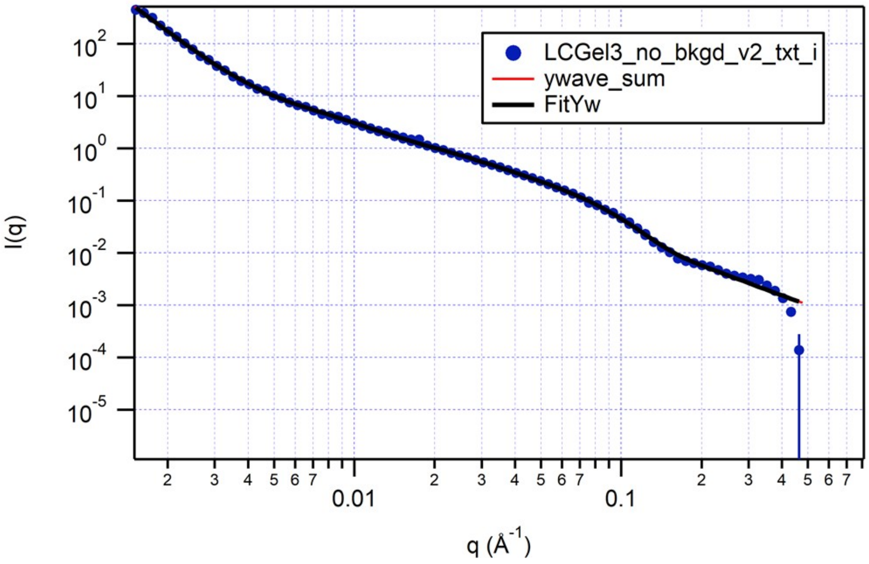

Coefficient, A	5.74E-09	±	6.44E-10
(-)Power	3.8776	±	0.017911
Incoherent Bgd (cm^{-1})	0	±	0
Scale	1	±	0
G_1 (cm^{-1} sr^{-1})	7.0146	±	0.135944
$R_{g,1}$ (Å)	197.821	±	2.17563
B_1 (cm^{-1} sr^{-1} A^{-Pow})	0.001269	±	0.000102
Pow_1	1.70581	±	0.019276
G_2 (cm^{-1} sr^{-1})	0.143323	±	0.008319
$R_{g,2}$ (Å)	22.455	±	0.271388
B_2 (cm^{-1} sr^{-1} A^{-Pow})	0.000216	±	2.11E-05
Pow_2	2.04256	±	0.065816
bkg (cm^{-1} sr^{-1})	0.000138	±	0
fit range	0.0015<q<0.5		
χ^2	2.5771		

<u>**LC-Gel 11 hr 15 min: Power Law + 2-Level Beaucage**</u>

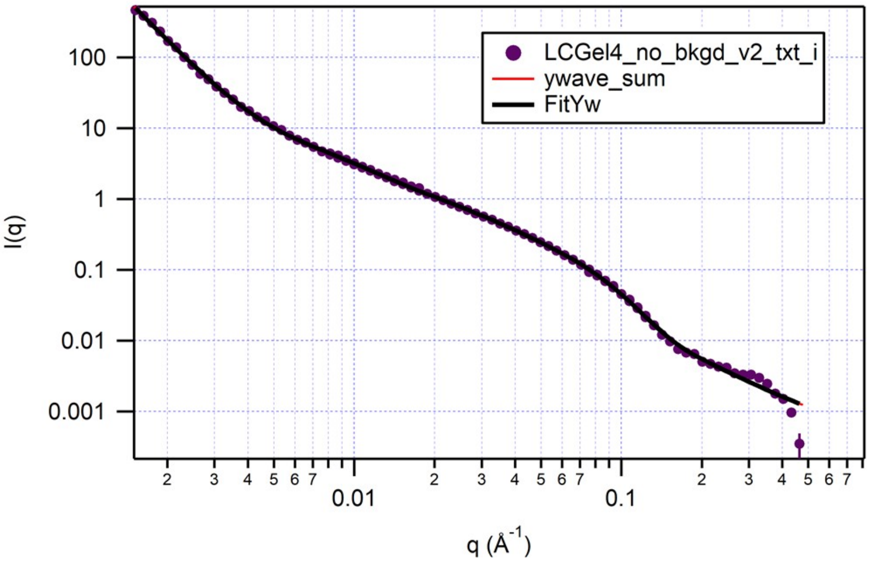

Coefficient, A	6.78E-09	±	7.15E-10
(-)Power	3.85307	±	0.016836
Incoherent Bgd (cm^{-1})	0	±	0
Scale	1	±	0
G_1 (cm^{-1} sr^{-1})	6.65732	±	0.116345
$R_{g,1}$ (Å)	187.789	±	1.9632
B_1 (cm^{-1} sr^{-1} A^{-Pow})	0.001194	±	0.0001
Pow_1	1.73568	±	0.020341
G_2 (cm^{-1} sr^{-1})	0.157514	±	0.008577
$R_{g,2}$ (Å)	22.8932	±	0.254438
B_2 (cm^{-1} sr^{-1} A^{-Pow})	0.000197	±	2.07E-05
Pow_2	2.03862	±	0.070545
bkg (cm^{-1} sr^{-1})	0.00035	±	0
fit range	0.0015<q<0.5		
χ^2	2.40019		

<u>**LC-Gel 12 hr 43 min: Power Law + 2-Level Beaucage**</u>

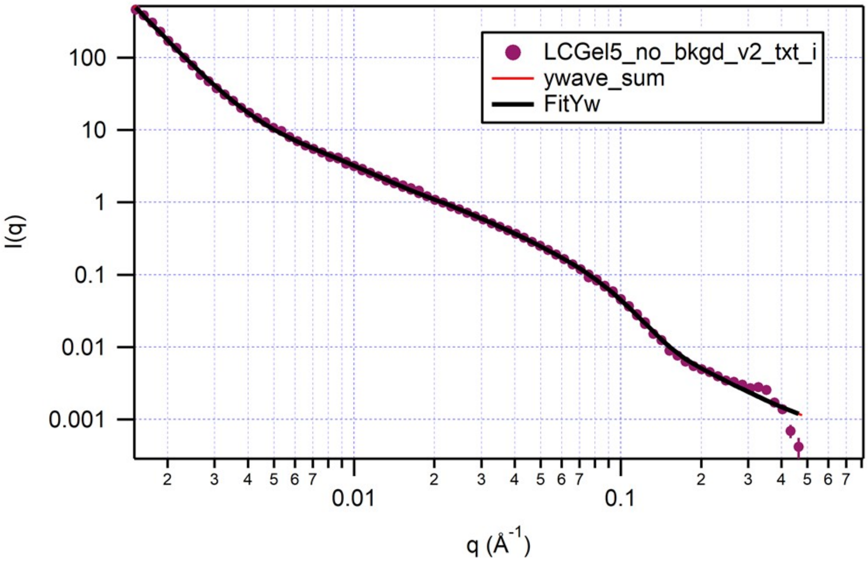

Coefficient, A	6.42E-09	±	6.85E-10
(-)Power	3.85888	±	0.017062
Incoherent Bgd (cm^{-1})	0	±	0
Scale	1	±	0
G_1 (cm^{-1} sr^{-1})	6.75336	±	0.11819
$R_{g,1}$ (Å)	188.577	±	1.99975
B_1 (cm^{-1} sr^{-1} A^{-Pow})	0.001261	±	0.000104
Pow_1	1.72802	±	0.020045
G_2 (cm^{-1} sr^{-1})	0.15626	±	0.008764
$R_{g,2}$ (Å)	22.9562	±	0.256082
B_2 (cm^{-1} sr^{-1} A^{-Pow})	0.000149	±	1.78E-05
Pow_2	2.15295	±	0.079224
bkg (cm^{-1} sr^{-1})	0.00042	±	0
fit range	0.0015<q<0.5		
χ^2	2.48849		

LC-Gel 13 hr 58 min: Power Law + 2-Level Beaucage

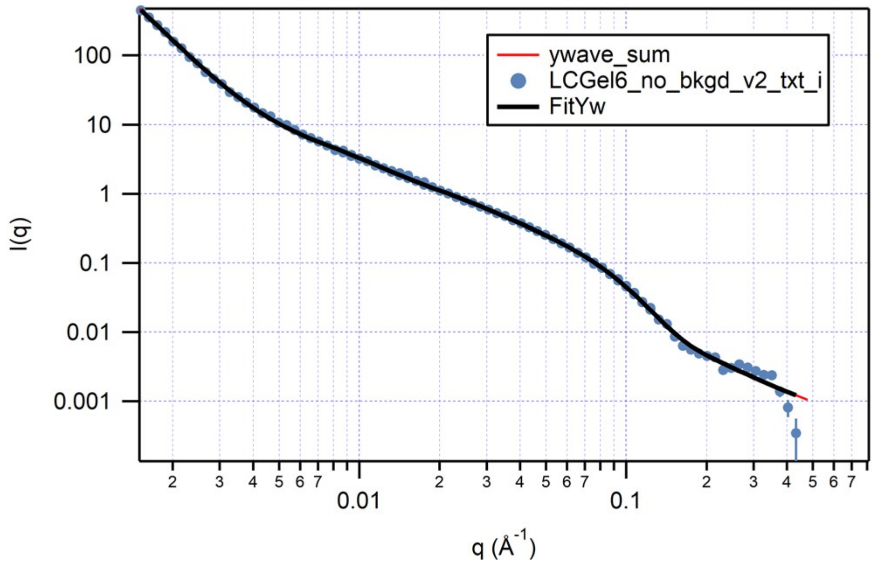

Coefficient, A	1.10E-08	±	1.24E-09
(-)Power	3.76445	±	0.018142
Incoherent Bgd (cm^{-1})	0	±	0
Scale	1	±	0
G_1 (cm^{-1} sr^{-1})	6.63386	±	0.142739
$R_{g,1}$ (Å)	184.467	±	2.49204
B_1 (cm^{-1} sr^{-1} A^{-Pow})	0.001341	±	0.000156
Pow_1	1.71837	±	0.02831
G_2 (cm^{-1} sr^{-1})	0.150781	±	0.012561
$R_{g,2}$ (Å)	22.7417	±	0.362216
B_2 (cm^{-1} sr^{-1} A^{-Pow})	0.000152	±	3.12E-05
Pow_2	2.07836	±	0.136864
bkg (cm^{-1} sr^{-1})	0.000348	±	0
fit range	0.0015<q<0.5		
χ^2	1.88858		

References

(1) Dolomanov, O. V.; Bourhis, L. J.; Gildea, R. J.; Howard, J. A. K.; Puschmann, H., OLEX2: a complete structure solution, refinement and analysis program. *J. Appl. Crystallogr.* **2009**, *42*, 339-341.

(2) Sheldrick, G. M., A short history of SHELX. *Acta Crystallogr. Sect. A* **2008**, *64*, 112-122.

(3) Mezger, T. G., *The Rheology Handbook*. 4th ed.; William Andrew Publishing: Norwich, NY, USA, 2012.

(4) Abrami, M.; D’Agostino, I.; Milcovich, G.; Fiorentino, S.; Farra, R.; Asaro, F.; Lapasin, R.; Grassi, G.; Grassi, M., Physical characterization of alginate–Pluronic F127 gel for endoluminal NABDs delivery. *Soft Matter* **2014**, *10*, 729-737.

(5) Boudara, V. A. H.; Read, D. J.; Ramírez, J., reptate rheology software: Toolkit for the analysis of theories and experiments. *J. Rheol.* **2020**, *64*, 709-722.

(6) Heller, W. T.; Hetrick, J.; Bilheux, J.; Calvo, J. M. B.; Chen, W.-R.; DeBeer-Schmitt, L.; Do, C.; Doucet, M.; Fitzsimmons, M. R.; Godoy, W. F.; Granroth, G. E.; Hahn, S.; He, L.; Islam, F.; Lin, J.; Littrell, K. C.; McDonnell, M.; McGaha, J.; Peterson, P. F.; Pingali, S.

V.; Qian, S.; Savici, A. T.; Shang, Y.; Stanley, C. B.; Urban, V. S.; Whitfield, R. E.; Zhang, C.; Zhou, W.; Billings, J. J.; Cuneo, M. J.; Leal, R. M. F.; Wang, T.; Wu, B., drtsans: The data reduction toolkit for small-angle neutron scattering at Oak Ridge National Laboratory. *SoftwareX* **2022**, *19*, 101101.

(7) Kline, S., Reduction and analysis of SANS and USANS data using IGOR Pro. *J. Appl. Crystallogr.* **2006**, *39*, 895-900.

(8) Thiele, C. M.; Petzold, K.; Schleucher, J., EASY ROESY: Reliable Cross-Peak Integration in Adiabatic Symmetrized ROESY. *Chem Eur. J.* **2009**, *15*, 585-588.

(9) Giessner-Prettre, C.; Pullman, B., Regions of negative values of intermolecular nuclear shielding for protons of purines. *J. Theor. Biol.* **1970**, *27*, 341-342.

(10) Nieuwenhuizen, M. M. L.; de Greef, T. F. A.; van der Bruggen, R. L. J.; Paulusse, J. M. J.; Appel, W. P. J.; Smulders, M. M. J.; Sijbesma, R. P.; Meijer, E. W., Self-Assembly of Ureido-Pyrimidinone Dimers into One-Dimensional Stacks by Lateral Hydrogen Bonding. *Chem Eur. J.* **2010**, *16*, 1601-1612.

(11) Macrae, C. F.; Bruno, I. J.; Chisholm, J. A.; Edgington, P. R.; McCabe, P.; Pidcock, E.; Rodriguez-Monge, L.; Taylor, R.; van de Streek, J.; Wood, P. A., Mercury CSD 2.0 - new features for the visualization and investigation of crystal structures. *J. Appl. Cryst.* **2008**, *41*, 466-470.
